# A DNA origami rotary ratchet motor

**DOI:** 10.1038/s41586-022-04910-y

**Published:** 2022-07-20

**Authors:** Anna-Katharina Pumm, Wouter Engelen, Enzo Kopperger, Jonas Isensee, Matthias Vogt, Viktorija Kozina, Massimo Kube, Maximilian N. Honemann, Eva Bertosin, Martin Langecker, Ramin Golestanian, Friedrich C. Simmel, Hendrik Dietz

**Affiliations:** 1 Lehrstuhl für Biomolekulare Nanotechnologie, Physik Department & Munich Institute of Biomedical Engineering, Technische Universität München, Garching near Munich, Germany; 2 Lehrstuhl für Physik Synthetischer Biosysteme, Physik Department, Technische Universität München, Garching near Munich, Germany; 3 Max Planck Institute for Dynamics and Self-Organization, Göttingen, Germany; 4 Rudolf Peierls Centre for Theoretical Physics, University of Oxford, Oxford, UK

**Keywords:** DNA nanotechnology, Biological physics, Single-molecule biophysics

## Abstract

To impart directionality to the motions of a molecular mechanism, one must overcome the random thermal forces that are ubiquitous on such small scales and in liquid solution at ambient temperature. In equilibrium without energy supply, directional motion cannot be sustained without violating the laws of thermodynamics. Under conditions away from thermodynamic equilibrium, directional motion may be achieved within the framework of Brownian ratchets, which are diffusive mechanisms that have broken inversion symmetry^1–5^. Ratcheting is thought to underpin the function of many natural biological motors, such as the F_1_F_0_-ATPase^6–8^, and it has been demonstrated experimentally in synthetic microscale systems (for example, to our knowledge, first in ref. ^3^) and also in artificial molecular motors created by organic chemical synthesis^9–12^. DNA nanotechnology^13^ has yielded a variety of nanoscale mechanisms, including pivots, hinges, crank sliders and rotary systems^14–17^, which can adopt different configurations, for example, triggered by strand-displacement reactions^18,19^ or by changing environmental parameters such as pH, ionic strength, temperature, external fields and by coupling their motions to those of natural motor proteins^20–26^. This previous work and considering low-Reynolds-number dynamics and inherent stochasticity^27,28^ led us to develop a nanoscale rotary motor built from DNA origami that is driven by ratcheting and whose mechanical capabilities approach those of biological motors such as F_1_F_0_-ATPase.

## Abstract (web summary)

A nanoscale rotary motor made of DNA origami, driven by ratcheting and powered by an external electric field, shows the ability to wind up a spring and has mechanical capabilities approaching those of biological motors.

## Main

**Fig. 1:**
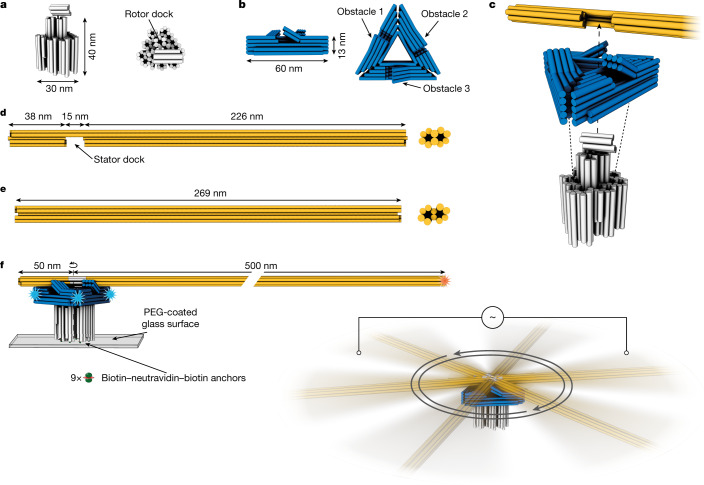
Motor design and experimental setup. **a**,**b**, Schematics of a pedestal and a triangular platform, respectively. Cylinders indicate DNA double helices. **c**, Schematic illustration of motor assembly steps. **d**,**e**, Rotor arm components. **f**, Left, schematic illustration of the experimental setup for observing motor dynamics in an inverted TIRF microscope. The pedestal is fixed through several biotin–neutravidin linkages to a microscope coverslip. Orange star, Cy5 dyes. Blue stars, labelling positions for DNA-PAINT imager strands. Right, two platinum electrodes are immersed in the liquid chamber from above and connected to a function generator generating a square-wave alternating current to create a fixed-axis energetic modulation that acts on all motors.

We used the methods of DNA origami^29,30^ to design and fabricate a 40 nm tall and 30 nm wide pedestal onto which we fixed an equilateral triangular platform with 60-nm-long edges and a thickness of 13 nm (Fig. 1a–c and Supplementary Figs. 1 and 2). A section of the pedestal that protrudes through the central cavity of the triangular platform includes a docking site for a rotor arm. The docking site is fixed by means of a pivot point consisting of three unpaired nucleotides near the midpoint of the triangular platform on the pedestal. The rotor arm in turn consists of two end-to-end joined rigid rod modules (each a separate DNA origami) (Fig. 1d,e and Supplementary Fig. 3) with a total length of 550 nm. The length of the rotor arm was chosen to enable tracking angular orientation changes of individual motors in real time in a diffraction-limited fluorescence microscope and to slow down angular motions through viscous friction with the solvent, inspired by the classic experiments by Kinosita and others that showed the rotation of individual F-actin-labelled F_1_-ATPase motors^8^. The rod modules consisted of ten DNA double helices arranged in a honeycomb lattice pattern (Fig. 1d,e). Such helical bundles have previously been shown to have persistence lengths in the several-micrometre regime^31^. The rotor arm can thus be regarded as a rigid but elastic rod. The rotor arm protrudes on either side of the pivot point beyond the confines of the triangular platform. With this design, the rotor arm is constrained sterically to uniaxial rotations around the pivot point within the plane of the triangle. We also installed physical obstacles on the three edges of the triangular platform (Fig. 1c). The obstacles consist of 18-nm-long rectangular plates that protrude with an inclination of about 50° from the surface of the triangular platform. The plates were held rigidly at this angle with a set of double-helical spacers. To overcome the obstacles when sweeping over the triangular platform, the rotor arm must bend upwards. The bending constitutes an energetic barrier that can trap the rotor in between obstacles in a Boltzmann-weighted fashion. The motor also includes functional modifications such as biotin moieties and fluorescent dyes (Fig. 1f) to enable experimental observation of the motion of individual motor particles. With the biotin moieties, the stators can be rigidly attached to microscope glass coverslips through several biotin–neutravidin bonds per stator, and the multiple fluorescent dyes at the tips of the rotary arm allow determining its orientation using centroid tracking^32^ relative to the position of the separately labelled triangular platform (Fig. 1f).

**Extended Data Fig. 1:**
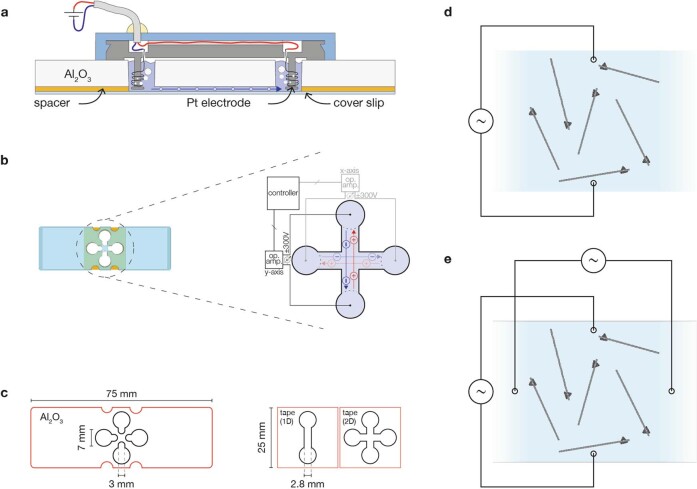
Experimental setup featuring electrodes for the application of AC fields. **a**, Scheme of the cross section of the sample chamber with inserted plug. **b**, Illustration of assembled sample chamber in top view and schematic of the resulting channel shape and electrode wiring (main electrode pair in black, second optional pair in grey). **c**, Dimensions of top component of sample chamber (left) and adhesive tape for one (middle) and two (right) electrode pairs in use. **d**, Schematic top view of rotors attached to the microscope coverslip with one electrode pair for a uniaxial AC field. **e**, Schematic top view of rotors attached to the microscope coverslip with two electrode pairs for the possibility of rotating the AC field axis.

**Extended Data Fig. 2:**
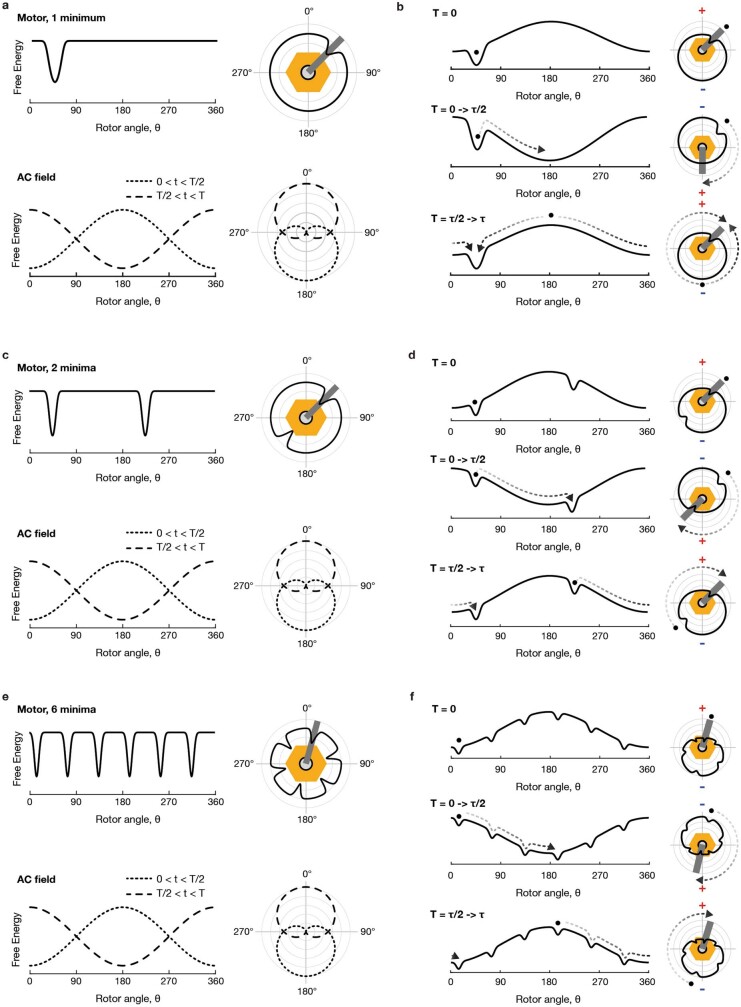
Illustration of motor mechanism. **a**,**c**,**e**, Solid line, schematic internal energy landscape for a simplified model motor featuring one (**a**), two (**c**) and six (**e**) minima. Dashed and dotted lines, the energetic contribution of an external AC field applied along the 0°–180° axis. The insets on the right give the energy functions in polar coordinates. **b**,**d**,**f**, Solid lines, snapshots of the effective energy landscape (that is, the sum of the internal motor energy plus field contribution) for a motor with one (**b**), two (**d**) and six (**f**) minima. Dashed lines give the expected trajectory of the rotor arm on field direction inversion. Initial position per field half-cycle is indicated by a dot.

In thermal equilibrium, any motion in any direction will be counterbalanced by the opposite motion such that the system will not be biased in the long-time limit; otherwise, we would have a perpetuum mobile. Our motor is thus designed to be operated as a ratchet under out-of-thermal-equilibrium conditions. To elicit an asymmetric, or biased, ratcheting effect, we apply a non-rotating electric alternating current (AC) field using electrodes immersed in the liquid chamber (Fig. 1f and see also Extended Data Fig. 1). The field causes an alternating ion current flowing through the sample chamber along a fixed axis. The time-averaged net force created by this external modulation is zero. There is no information supplied by the external modulation that could dictate a rotation of the motor. Instead, depending on the nature and location of energetic minima in the motor relative to the axis of the electric field, the modulation can produce a kinetic asymmetry per field cycle that causes the motor to move with a preferred rotation direction (Extended Data Fig. 2).

**Fig. 2:**
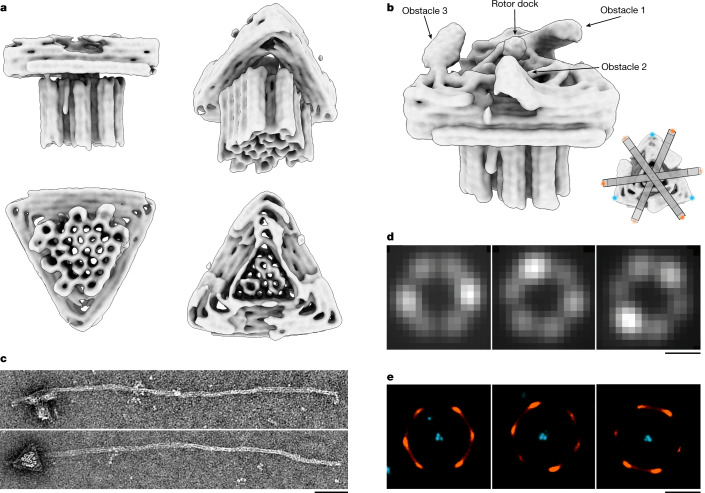
Structural analysis of the DNA origami motor. **a**, Different views of a 3D electron density map of the motor block determined by means of single-particle cryo-EM (see also Extended Data Fig. 4 and in the Electron Microscopy Data Bank (EMDB) under code EMD-14358). **b**, Motor block cryo-EM map detail depicted at different density thresholds at which the three obstacles and the rotor dock can be discerned. Inset, schematic showing the six preferred dwelling sites of the rotor arm. **c**, Exemplary negative-staining TEM images of a motor variant with long rotor arm attached. Scale bar, 50 nm. **d**, Exemplary single-particle fluorescence images. Scale bar, 500 nm. The images show the standard deviation of the mean intensity per pixel computed over all the frames from recorded TIRF videos. **e**, DNA-PAINT images showing rotor arm tip positions relative to the triangle platform. Scale bar, 500 nm.

**Extended Data Fig. 3:**
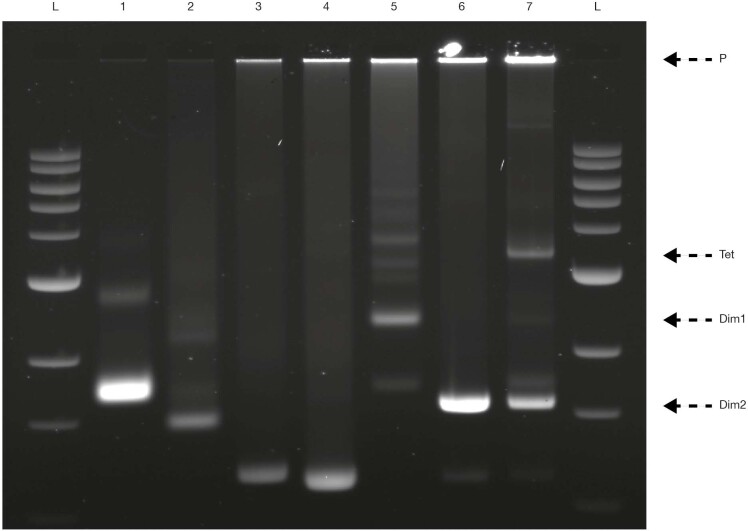
Gel-electrophoretic analysis of motor assembly. Laser-scanned image of a 1.5% agarose gel with 5.5 mM MgCl_2_ run on a water bath at 100 V for 165 min on which the following samples were electrophoresed: L, 1-kb ladder; 1, triangular platform; 2, pedestal; 3, rotor arm first part; 4, rotor arm second part (extension); 5, triangular platform and pedestal dimer; 6, rotor arm dimer; 7, fully assembled tetramer. P, pockets; Tet, tetramer; Dim1, dimer of triangular platform and pedestal; Dim2, dimer of rotor arm.

**Extended Data Fig. 4:**
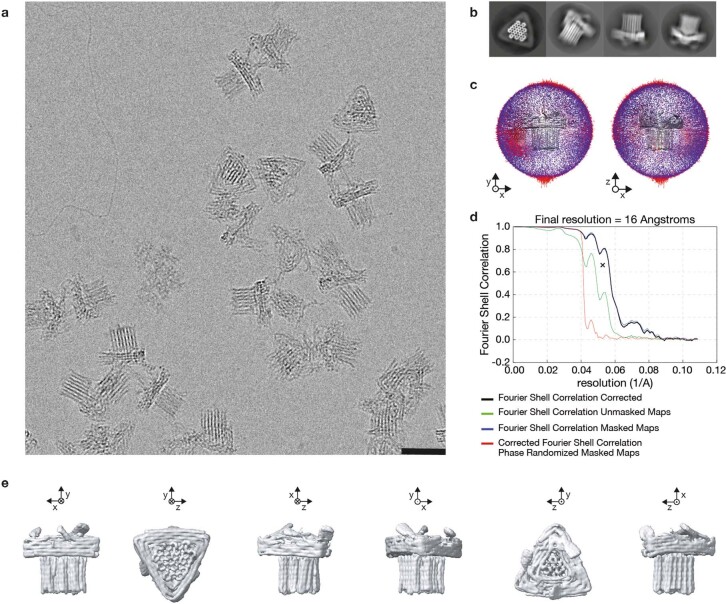
Cryo-EM reconstruction of the motor block. **a**, Exemplary micrograph. Scale bar, 50 nm. **b**, Representative 2D class averages. **c**, Histogram representing the orientational distribution of particles. **d**, Fourier shell correlation plot. **e**, Six different views of the electron density map.

We encoded our motor design in DNA sequences^29^ (Supplementary Datasets 1–5) and self-assembled the motors in one-pot reaction mixtures using previously described procedures^33^. We assessed the quality of self-assembly using gel-electrophoretic mobility analysis (Extended Data Fig. 3) and validated the 3D shape of the motor complex, including the pedestal, the triangular platform and the rotor dock, with a 3D electron density map that we determined using single-particle cryo-electron microscopy (cryo-EM) (Fig. 2a,b and Extended Data Fig. 4). Within the resolution, the electron density map showed all the desired main structural features, including the obstacles and the rotor dock. We also validated the correct assembly of the full motor complex featuring the full-length rotor arm by imaging with negative-staining transmission electron microscopy (TEM) (Fig. 2c).

**Extended Data Fig. 5:**
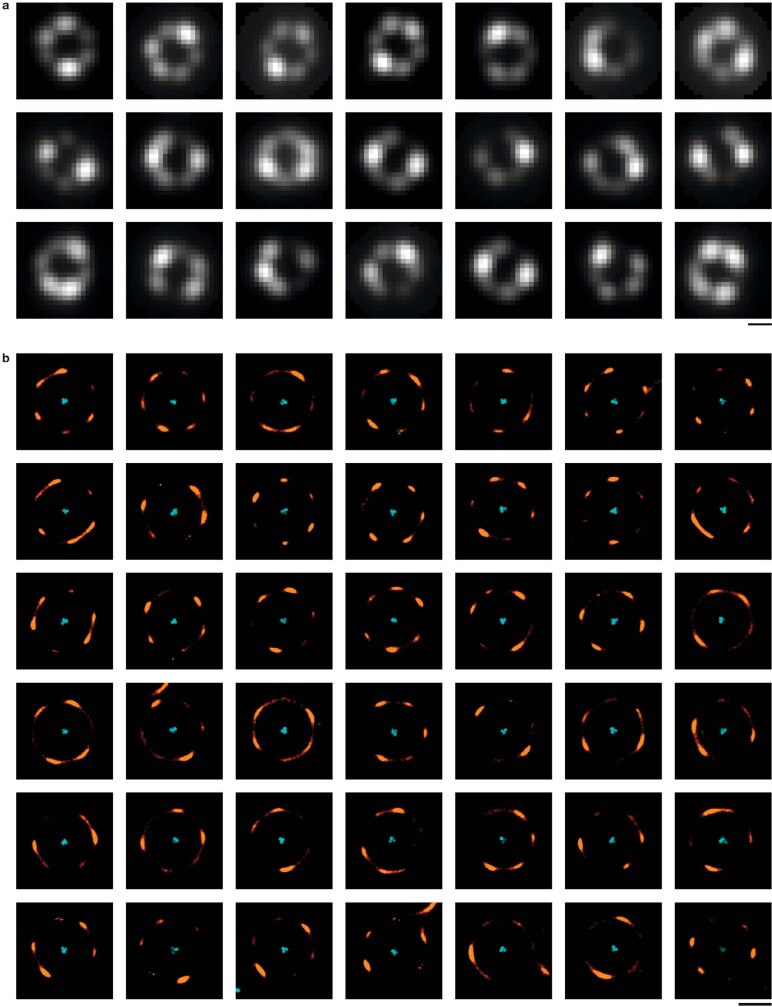
Exemplary single images of standard deviation and DNA-PAINT images. **a**, Standard deviation of fluorescence intensity from single-particle TIRF videos. Scale bar, 500 nm. The images show the standard deviation of the mean intensity per pixel computed over all the frames from recorded TIRF videos. **b**, DNA-PAINT images showing rotor arm tip positions (orange) relative to the triangular platform (blue). Scale bar, 500 nm.

**Extended Data Fig. 6:**
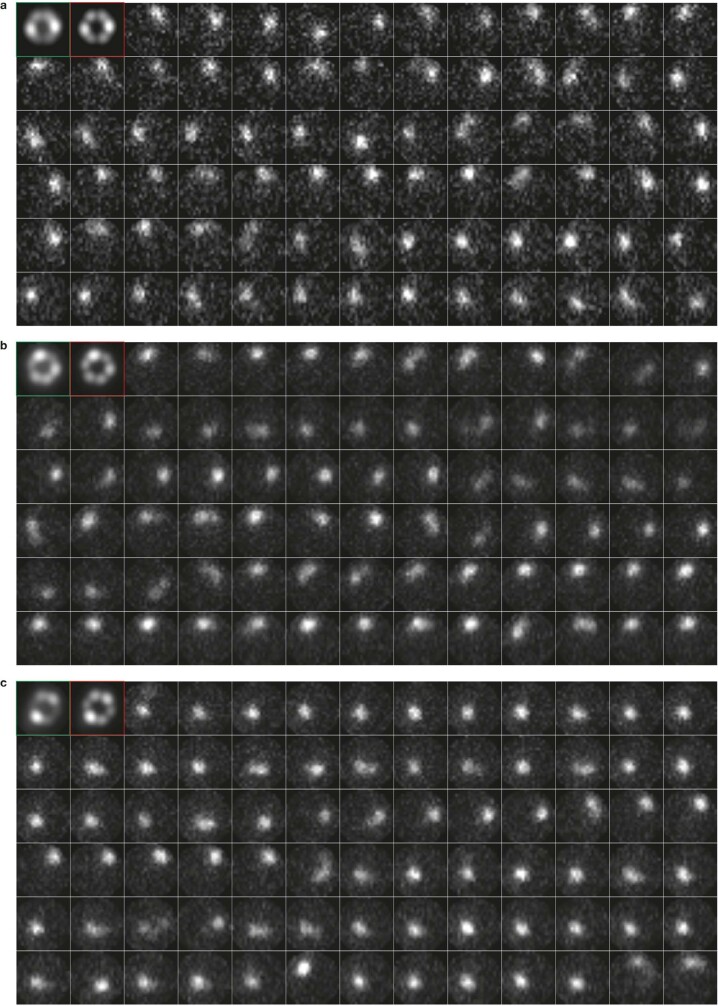
Single-frame TIRF images. **a**–**c**, Montage of first 76 individual frame images from the particles given in Fig. 2 (left (**a**), middle (**b**) and right (**c**) particles). Green box, average intensity of the entire video. Red box, standard deviation of the mean intensity per pixel computed from the entire video.

**Extended Data Fig. 7:**
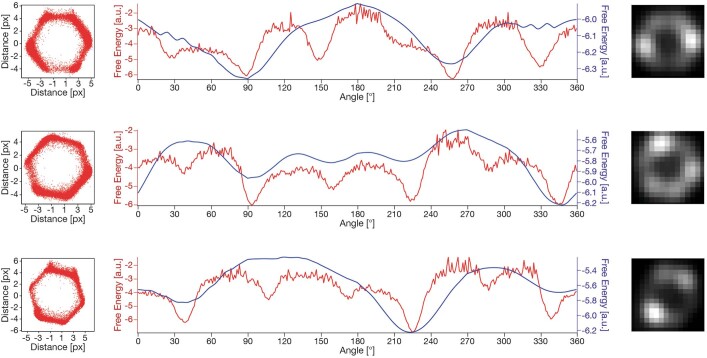
Exemplary free-energy surfaces with six minima (two dominant). Left, scatter plot of rotor arm tip position using a virtual window centre of mass (vwcm) approach. Middle, free energy computed from centroid tracking (red) and from radial pixel intensities (blue). Right, average of the mean intensity per pixel computed over all frames.

Total internal reflection fluorescence (TIRF) microscopy of surface-immobilized motor particles in equilibrium showed rotating particles in which the rotor arm preferentially dwelled in six discrete positions (Fig. 2d and Extended Data Figs. 5a and 6). These positions corresponded to orientations with the rotor arm trapped on either side of the protruding obstacles, which we established using DNA-PAINT imaging^34^ (Fig. 2e and Extended Data Fig. 5b). The DNA-PAINT images also provide a compelling illustration of the relative dimensions of the triangular platform versus the much longer rotor arm. Our motor complex thus realizes a diffusive rotary mechanism featuring several energetic minima (Extended Data Fig. 7).

**Fig. 3:**
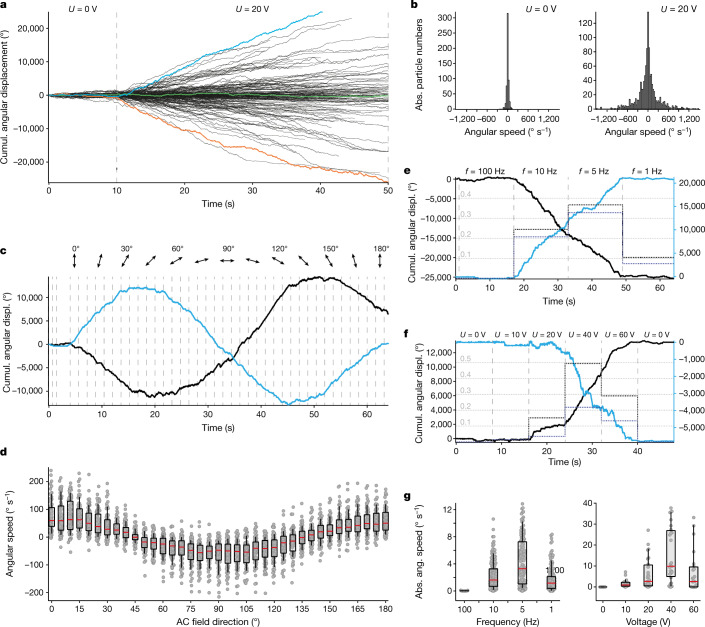
Motor dynamics. **a**, Exemplary single-particle traces showing cumulative angular displacement of rotor arm tips, with the AC field off during the first 10 s. Blue and orange, exemplary motors rotating CW and CCW, respectively; green, a particle that continued wiggling without apparent bias even when the field was on. The AC field was a 5-Hz square wave with amplitude 20 V, unless otherwise specified. A close-up of the first 10 s with the AC field off can be found in Extended Data Fig. 8. **b**, Histograms of the angular speed of single particles with field off (left, *N* = 557) versus field on (right, *N* = 1,078). **c**, Exemplary single motor traces showing the influence of AC field axis orientation on motor speed. The AC field axis was rotated stepwise in 5° increments. Dashed lines indicate time points when the field direction was updated. **d**, Scatter plot of phase-corrected angular speeds for different AC field axes (*N* = 75). The box plots show the 25th and 75th percentiles, with the whiskers indicating the 10th and 90th percentiles. Red lines within the boxes mark the median. **e**, Solid lines, exemplary single-particle traces seen during an AC frequency sweep, showing a CCW and a CW rotating motor. Dotted lines give the effective angular speed as turns/field cycles. **f**, Solid lines, exemplary single-particle traces seen during a voltage sweep, showing a CCW and a CW rotating motor. Dotted lines give the directional bias efficiency as in **e**. **g**, Scatter plot of absolute angular speeds per AC field cycle for different frequencies (left, *N* = 156) and different voltages (right, *N* = 28). Box and whisker plots as in **d**. See also Supplementary Videos 1–8. Source data

**Extended Data Fig. 8:**
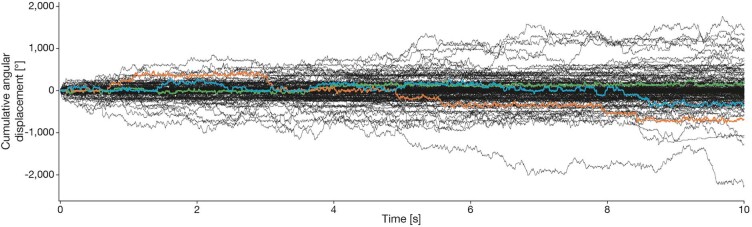
Single-particle angle–time traces in the absence of an AC field. Zoom-in of exemplary single-particle traces showing cumulative angular displacement of rotor arm tips as shown in Fig. 3a with the AC field off during the first 10 s. Coloured traces are the same as in Fig. 3a.

We used centroid tracking^32^ to more accurately determine the rotor arm orientation per frame from the single-particle TIRF videos. In equilibrium, that is, when the external field was off, the motor particles showed unbiased random rotary movements with vanishing cumulative angular displacements, as expected from equilibrium fluctuations in an energy landscape (Fig. 3a and Extended Data Fig. 8). By contrast, when we turned on the AC field, a fraction of the motor particles (32.3%) immediately changed from random, undirected rotation to processive rotation with a directional bias (Figs. 3a and Supplementary Videos 1 and 2). The maximum angular velocity we recorded was approximately 250 full turns per minute and approximately equal numbers of motors rotated processively in clockwise (CW) and counterclockwise (CCW) directions when the field was turned on (Fig. 3b).

**Extended Data Fig. 9:**
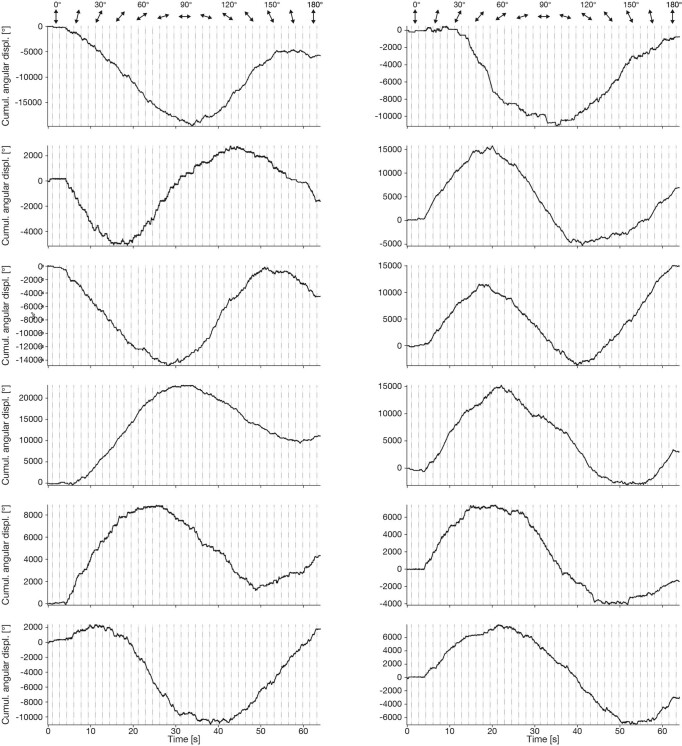
Exemplary single-particle traces showing the influence of AC field axis orientation on motor rotation. The AC field axis was rotated stepwise in 5° increments every 1.6 s. Dashed lines indicate time points when the field direction was updated Source data

The direction of rotation and the effective angular speed of each motor particle could be controlled by the orientation of the AC field axis relative to the motor particles (which are fixed on the substrate). To demonstrate this property, we used a four-electrode setup that allowed us to tune the effective direction of the AC field axis by a superposition of electric fields applied in the *x* and *y* directions of the glass coverslip plane. In one set of experiments, we rotated the AC field axis in increments of 5° every 1.6 s while recording and tracking single motor motions. As a result, we obtained single-particle trajectories that give cumulative angular displacement per single particle as a function of time (Fig. 3c and Supplementary Videos 3 and 4). From these data, we computed the effective angular speed of each motor and plotted these as a function of the orientation that the field axis had in each 1.6-s increment (Extended Data Fig. 9). For most motors, we observe a sinusoidal dependency of motor speed on field direction, including stalling and direction reversal. Each motor has a specific AC field orientation for which the motor shows maximum and minimum speed. We attribute these 'phase shifts' between motor speeds to the fact that the motors are fixed with random orientations on the microscopy coverslip. We aligned the angular speed versus field orientation data and computed the average and standard deviation of angular speed over an ensemble of 75 motor particles, which provides an impression of the motor-to-motor speed variability (Fig. 3d). We also characterized the dynamics of individual motors as a function of AC field frequency and amplitude (Fig. 3e–g and Supplementary Videos 5–8). The effective angular velocity of the motors depended on AC frequency, with an optimum at 5-Hz driving frequency. The directional bias of rotation was absent when applying DC fields and it also vanished at high AC frequencies (100 Hz). Similarly, the angular velocity of the motors could also be controlled by the AC field amplitude, showing an optimal effective angular velocity in the amplitude band between 20 and 60 V.

On the basis of the data recorded, we can estimate the torque and the work done on the environment by the motors, which—in our experiments for Fig. 3—was dissipated in the form of frictional drag of the long rotor arm with the solvent. The rotational friction coefficient for the rotor arm $({\zeta }_{{\rm{r}}}=\pi \eta {L}^{3})$ is approximately 4 × 10^−22^ N m s. On the basis of the maximum observed angular velocity of 25 radians s^−1^ (1,500° s^−1^), we arrive at a maximum torque of approximately 10 pN nm, which may be compared with the 50 pN nm that F_1_F_0_-ATPase can generate^35^. The estimated maximum power of our motors dissipated in friction was 250 pN nm s^−1^ (62 k_B_T s^−1^), which corresponds to the equivalent of the free energy delivered by the hydrolysis of approximately 2.5 ATP molecules per second at cellular conditions.

**Fig. 4:**
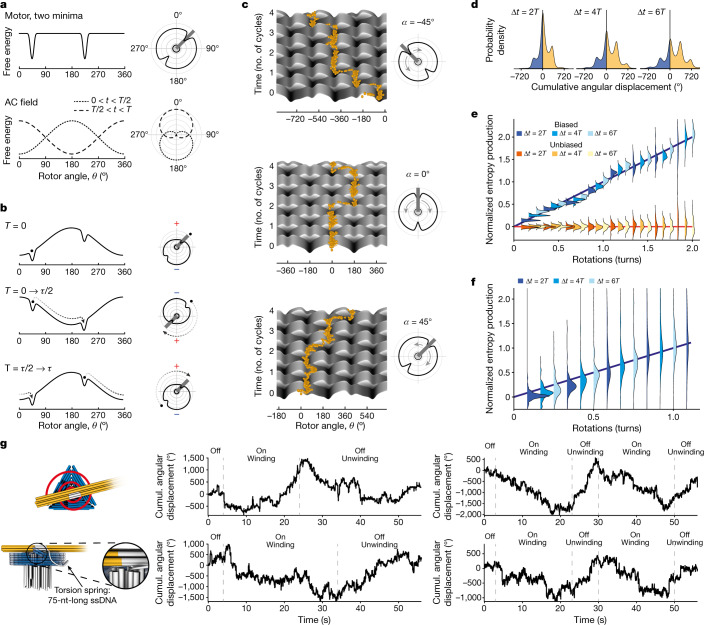
Motor mechanism, fluctuation analysis and winding up a spindle. **a**, Solid line, schematic internal energy landscape for a simplified two-minima motor. Dashed and dotted lines, energetic contribution of external AC field applied along the 0°–180° axis. Insets, energy functions in polar coordinates. **b**, Solid lines, snapshots of the sum of internal motor energy plus field contribution. Dashed lines, expected trajectory of the rotor arm on field direction inversion. Initial position per field half-cycle is indicated by a dot. **c**, Energy landscapes for the hypothetical two-minima motor from **a** and **b** plotted as 2D space-time surfaces and computed for three exemplary orientations of motor relative to field axis (see insets). Field axis is along the 0°–180° direction. Yellow dots, exemplary single-particle trajectories as simulated by Langevin dynamics. **d**, Distribution of cumulative angular displacements after two, four and six AC field cycles aggregated from a simulated Langevin dynamics trajectory. The prominent peaks at 180° intervals are due to the twofold symmetry in the simulated energy landscape. **e**, Irreversibility analysis for simulated motors. Shown is an ensemble average over 20 simulated motors evaluated at different displacement values and for time intervals of two, four and six AC cycles, as indicated by colour. The motors contain a twofold symmetry as illustrated in panel **a**, in which in the blue scheme motor orientations with 45° relative to the field were used and in the red scheme symmetric orientations (0°) were used. Solid lines, guides to the eye to emphasize the trend or the lack thereof. **f**, Irreversibility analysis of experimentally observed motor particles. Colouring of the distribution indicates the time interval as in panel **d**. To account for variations in rotation speed, the linear trend in each rotor was renormalized to a slope of unity before computing the distributions shown. A line with unit slope was added to guide the eye. See Extended Data Fig. 10b for the irreversibility analysis of experimentally observed particles. **g**, Left, schematics of a motor variant that includes a ssDNA torsional spring at the pivot point. Right, exemplary experimentally observed cumulative angular displacement of single motor particles. First phase: AC field off, spring relaxed. Second phase: AC field on, spring is wound up. Third phase: AC field off, spring is unwinding and driving the motion. See also Supplementary Videos 12 and 13. Source data

We can gain insight into the mechanism of our motors by considering the effective energy landscapes in Fig. 4a–c, computed exemplarily for different stator-field orientations of a simplified two-minima rotary motor (see also Extended Data Fig. 2). The asymmetry needed for net directionality selection (bias or ratcheting) is created by the interplay between the background static potential landscape provided by the motor body and the external modulating field. The extent of asymmetry depends on the orientation of the motor energy landscape relative to the field (Fig. 4c), as illustrated exemplarily by Langevin dynamics simulations in these energy landscapes that lead to rotation trajectories with CW, CCW and absence of directional bias, respectively. Because we randomly deposited motor particles on microscopy cover slides in our experiments, we obtained an approximately uniform sampling of motor orientations relative to the field axis, and thus a sampling of motors rotating CW or CCW at various speeds (Fig. 3c,d). Supplementary Videos 9–11 summarize schematically how the expected motor speed depends on field orientation, frequency and amplitude.

**Extended Data Fig. 10:**
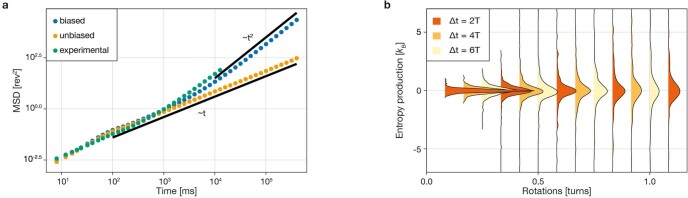
Mean square displacement and irreversibility analysis of motors. **a**, Mean square angular displacement of experimentally observed and simulated motors. We compare experimental data (green dots) with simulations performed in a symmetric landscape (yellow) and in a biased one (blue). The unbiased simulation behaves diffusively at long times. The biased simulation and experimental data both behave diffusively at short times but transition to ballistic motion at long times. **b**, Irreversibility analysis of experimentally observed motor particles without an external electric field. Colouring of the distribution indicates the time interval with $T=\frac{1}{f}=0.2\,{\rm{s}}$. Different from Fig. 4e, no renormalization was applied, as a clear trend is lacking.

To test the irreversibility of our mechanism at the level of individual motors, we analysed the properties of their fluctuations in the context of stochastic thermodynamics^36^. If we compile the distribution of displacement angles as a function of time (at multiples of the AC field period *T*), we expect that the rotors will naturally take stochastic steps along the direction of the bias and also against it (Fig. 4d and see also Fig. 3a). Stochastic thermodynamics allows us to investigate the degree of entropy production in a non-equilibrium system with a finite trajectory, from the ratio between the upstream and downstream transition rates. Denoting ${\rm{P}}\left({\theta }_{0}+\Delta \theta ,t| {\theta }_{0,}0\right)$ as the probability of rotation by angle Δ*θ* over the period *t* from the initial position of *θ*_0_, we expect a fluctuation relation that explores irreversibility by extracting the average entropy production Δ*s* in the nanomotors over the period of time *t* = *nT* (an integer multiple of the period of the AC field), in the form

1 $$\frac{\Delta s}{{k}_{{\rm{B}}}}\equiv {\left\langle {\rm{ln}}\left[\frac{{\rm{P}}\left({\theta }_{0}+\Delta \theta ,t=nT| {\theta }_{0,}0\right)}{{\rm{P}}\left({\theta }_{0},t=nT| {\theta }_{0}+\Delta \theta ,0\right)}\right]\right\rangle }_{{\theta }_{0}}=\frac{{\omega }_{{\rm{eff}}}}{{D}_{{\rm{eff}}}}\,\Delta \theta $$

in which *ω*_eff_ and *D*_eff_ represent the effective angular velocity and diffusion coefficient, respectively, of the nanomotors throughout the stochastic ratcheting dynamics. The effective diffusion coefficient is related to an effective friction coefficient *ζ*_eff_, which depends on the microscopic mechanism of the non-equilibrium drive. Note that equation (1) is independent of time *t* = *nT*. This relation holds in the simulations that we performed (Fig. 4e) and it also holds in the experimental data of processive rotating motors (Fig. 4f), as seen by the collapse of all the plots to a line of slope unity in the non-equilibrium cases through a rescaling of the slopes. The non-equilibrium drive can also be investigated in the mean squared displacement that shows a crossover from diffusive to ballistic behaviour (Extended Data Fig. 10a). We can present a rough theoretical estimate of the efficiency of the motor. When the system operates against an external torque *τ*, its net angular velocity will be ${\omega }_{{\rm{eff}}}\left(\tau \right)={\omega }_{{\rm{eff}}}-\tau /{\zeta }_{{\rm{r}}}$, from which the useful work per unit time of ${\omega }_{{\rm{eff}}}\left(\tau \right)\times \tau =({\omega }_{{\rm{eff}}}-\tau /{\zeta }_{r})\times \tau$ can be extracted, with a nominal efficiency estimate of $\epsilon \equiv \frac{({\omega }_{{\rm{eff}}}-\tau /{\zeta }_{{\rm{r}}})\times \tau \,}{{{\rm{\zeta }}}_{{\rm{eff}}}{\times {\rm{\omega }}}_{{\rm{eff}}}^{2}}$ . Therefore, the maximum work can be extracted when $\tau ={\zeta }_{{\rm{r}}}{\omega }_{{\rm{eff}}}/2$, leading to a bound on the nominal efficiency of $\epsilon \le \frac{{\zeta }_{{\rm{r}}}\,}{{4{\rm{\zeta }}}_{{\rm{eff}}}}$.

**Extended Data Fig. 11:**
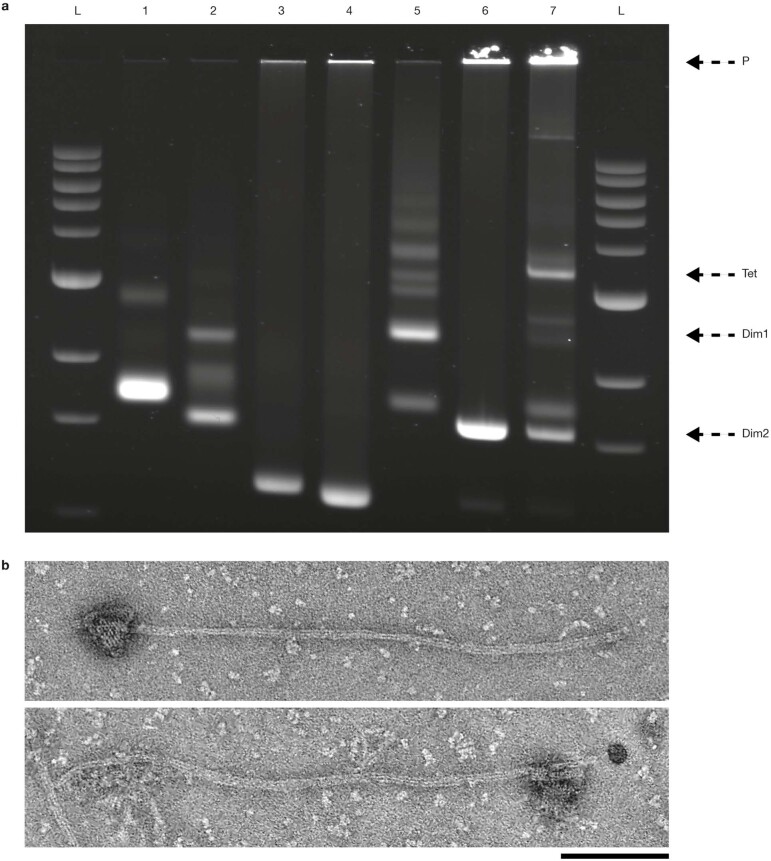
Assembly of the rotary apparatus with torsional spring. **a**, Laser-scanned image of a 1.5% agarose gel with 5.5 mM MgCl_2_ run on a water bath at 100 V for 165 min on which the following samples were electrophoresed: L, 1-kb ladder; 1, triangular platform; 2, pedestal; 3, rotor arm first part; 4, rotor arm second part (extension); 5, triangular platform and pedestal dimer; 6, rotor arm dimer; 7, fully assembled tetramer. P, pockets; Tet, tetramer; Dim1, dimer of triangular platform and pedestal; Dim2, dimer of rotor arm. **b**, Exemplary negative staining TEM images of the full motor assembly with attached rotor arm imaged with a Philips CM100 microscope. Scale bar, 100 nm.

We also tested whether our motors could generate torque against a further load. To this end, we designed motor variants that included a torsional spring at the pivot point (Fig. 4g, Extended Data Fig. 11 and Supplementary Fig. 4). The torsional spring consisted of a single-stranded DNA loop with one end fixed on the pedestal and the other on the rotor arm. Rotor rotations will wind the loop as an entropic spring around the rotor pivot connection. The winding produces a restoring force that eventually makes the motor stall once the torque created by the wound-up spring balances the maximum torque delivered by the motor. The thus tensioned spring then serves as an energy reservoir that can drive the rotor in the opposite direction when the external energy supply is shut off, until the spring is relaxed again. This predicted behaviour corresponds to what we observed: individual particles featuring the torsion spring showed processive rotation when the AC field was on until stalling, and then immediately began to rotate processively into the opposite direction once the AC field was shut off (Fig. 4g and Supplementary Videos 12 and 13).

In conclusion, our macromolecular rotary motors can perform work, as evidenced by their sustained rotation against viscous drag in solution and by their capability to wind up a molecular torsion spring. With angular speeds up to 250 revolutions per minute and torques up to 10 pN nm, the motors achieve rotational speeds and torques that are approaching those known from powerful natural molecular machines, such as the ATP synthase. The motors move directionally owing to intrinsic mechanistic properties, powered by a simple external energy modulation that does not need any feedback or information supplied by the user to direct the motors. Our motors also afford control options that one is familiar with from macroscale motors: the user can turn them on and off at will, they respond quickly and the speed and direction of rotation can be regulated. The motors can be produced and operated by anyone having access to standard wet-lab equipment. It suffices to transmit the sequence information to enable other users to replicate and build their own motors using DNA molecules obtained, for example, from commercial sources. The production of the required DNA molecules can be scaled to mass quantities^37^. Owing to the modularity of the DNA origami components, we expect that the motors can also be modified, adapted and integrated into other contexts. Gopinath et al. have recently described how to place and orient DNA origami objects on patterned solid-state surfaces in a programmable fashion^38,39^. These methods could be used to build arrays of motors with controlled stator orientation relative to the field axis, to achieve synchronized rotation^40^. Our motor design and operating concept might also be applicable to other systems beyond DNA origami. For example, protein-based rotary assemblies featuring charged residues could presumably be designed de novo and could be driven to rotate with directional bias by AC fields. Furthermore, instead of electric fields, other energy supplies that alternate in directionality, such as alternating fluid flows, could presumably be used as well. A natural next frontier would be to explore causing a barrier modulation through a chemical reaction and to exploit the directed motor motion to drive uphill chemical synthesis using a more elaborate synthetic mechanism featuring coordinated reciprocal motion—much like F_1_F0-ATP synthase mechanically synthesizes ATP driven by rotary motion.

## Methods

### Design of the DNA origami nanostructures

All structures were designed using cadnano 0.2 (ref. ^41^). The pedestal was folded from a 7,585-bases-long linearized custom scaffold, whereas the pedestal with torsional spring was folded from an 8,064-bases-long scaffold, as well as both rotor arm parts. The triangular platform was folded from a 9,072-bases-long scaffold^42^.

### Custom scaffold preparation

The circular scaffold with a length of 8,064 bases was prepared as described previously^43^ from a 2 l stirred bioreactor. The circular scaffold of length 9,072 bases, as well as the circular precursor of the linear scaffold of length 7,585 bases, were prepared from shaking flask cultures as previously described^42^. To linearize the scaffold, it was zinc digested as described previously^37^.

### Folding of the DNA origami nanostructures

All folding reaction mixtures contained a final scaffold concentration of 50 nM and oligonucleotide strands (Integrated DNA Technologies (IDT)) of 500 nM each (for the triangular platform) or 200 nM each (for the other structures). The folding reaction buffers contained 5 mM tris(hydroxymethyl)aminomethane hydrochloride (TRIS-HCl), 1 mM ethylenediaminetetraacetic acid (EDTA), 5 mM NaCl and 10 mM (rotor arms), 15 mM (both pedestal variants) or 20 mM (triangular platform) MgCl_2_. The folding solutions were thermally annealed using Tetrad (MJ Research, now Bio-Rad) thermal cycling devices. The reactions were left at 65 °C for 15 min and subsequently subjected to a thermal annealing ramp from 60 °C to 44 °C (1 °C h^−1^). The folded structures were stored at room temperature until further sample preparation steps. All DNA sequences are available in Supplementary Data Tables 1–5.

### Purification and concentration of the DNA origami nanostructures

All folded structures were purified from excess oligonucleotides either by polyethylene glycol (PEG) precipitation (rotor arms and pedestal variants) or by physical extraction from agarose gels (triangular platform). Gel-purified monomers were concentrated using ultracentrifugation. The PEG-purified rotor arm extension was further incubated with a set of connecting oligonucleotide strands at a MgCl_2_ concentration of 10 mM for 1 h at 30 °C and subsequently PEG precipitated again. All procedures were performed as previously described^33^.

### Assembly of the rotary apparatus

As a first step, the two dimers, triangular platform and pedestal (dimer 1), as well as the two rotor arm parts (dimer 2), were assembled by mixing a 1:1 solution of the respective monomers at a final MgCl_2_ concentration of 40 mM (dimer 1) and 5 mM (dimer 2), and left at 40 °C for at least 16 h. Dimer 1 was then PEG precipitated to exchange the buffer to a final MgCl_2_ concentration of 5 mM. Both dimers were mixed and incubated at 10 mM MgCl_2_ for a minimum of 16 h.

### Agarose gel analysis of the DNA origami nanostructures

Folded and assembled DNA nanostructures were electrophoresed on 1.5% or 2% agarose gels containing 0.5× tris-borate-EDTA and 5.5 mM MgCl_2_ for 1.5–3 h at 90 or 100 V bias voltage in a water-cooled gel box. The electrophoresed agarose gels were stained with ethidium bromide and scanned using a Typhoon FLA 9500 laser scanner (GE Healthcare) at a resolution of 50 μm per pixel.

### Negative-staining TEM

A quantity of sample of 5 μl was adsorbed onto glow-discharged Cu grids with carbon support (in-house production and Science Services, Munich) and stained with a 2% aqueous uranyl formate solution containing 25 mM NaOH. Samples were incubated for different lengths of time depending on the concentration. In general, structures with concentrations on the order of tens of nM were incubated for 30 s, whereas lower concentrated samples (5 nM or below) were incubated for 5 to 10 min. Images were acquired using a Philips CM100 microscope operating at 100 kV.

### Cryo-EM sample preparation

The purified and concentrated sample was applied to glow-discharged C-Flat 2/1-4C (EMS) grids (Protochips) and plunge frozen using a Vitrobot Mark V (FEI, now Thermo Scientific) at the following settings: temperature of 22 °C, humidity of 90%, 0 s wait time, 3 s blot time, −1 blot force, 0 s drain time.

### Cryo-EM image acquisition

The data were acquired on a Titan Krios G2 electron microscope operated at 300 kV equipped with a Falcon 3 direct detector using the EPU software (Thermo Scientific). A total exposure of 3.3 s with a dose of 44 e Angstrom^−2^ split in 11 fractions was used.

### Cryo-EM image processing

The image processing was performed in RELION 3.0 (refs. ^44,45^). The micrographs were motion corrected and contrast transfer function estimated using MotionCor2 (ref. ^46^) and CTFFIND4.1 (ref. ^47^), respectively. The particles were picked using crYOLO^48^. The auto-picked particles were extracted from the micrographs and binned by 2, subjected to one round of 2D and 3D classification to remove falsely picked grid contaminations and damaged particles, and to address structural heterogeneity. A refined 3D map was reconstructed using a low-resolution initial model created in RELION. A total number of 38,649 particles was used for the final reconstruction. The map was post-processed using a low-pass-filtered mask to calculate the Fourier shell correlations and estimate the global resolution of 16 Angstroms with a manually set B-factor of −500.

### Sample preparation for fluorescence measurements

Monomers were folded, purified and assembled as described before. Biotinylated oligos were incubated with a 32× excess of neutravidin (Thermo Fisher Scientific) and then added to the polymers in a roughly 10× excess to binding site for 1–2 h at room temperature. The resulting reaction mixture was gel purified by extracting only the tetrameric species. Sample concentrations were about 100 pM. If needed, a set of two spacer oligonucleotide strands was added in an approximately 100× excess to the sample to mount the obstacles on the triangular platform. All samples were stored at room temperature until imaged at the microscope up to several weeks.

### TIRF microscopy video acquisition with AC field

Biotin–PEG cover glass slide preparation, flow chamber production and TIRF microscopy setup are as previously described in ref. ^25^. The samples were diluted to below 100 pM in an imaging buffer (FMB 500) containing 500 mM NaCl, 100 mM TRIS-HCl and 2 mM EDTA, added to the sample chamber and immobilized on the glass surface through biotin–streptavidin–biotin linkage. Unbound structures were removed by flushing with FMB 500 after about 5 min. The sample chamber was then flushed twice with the final imaging buffer (FMB 1.5) containing 150 mM TRIS-HCl, 1 mM EDTA, 1.5 M NaCl and including an oxygen scavenging system with 2 mM Trolox (6-hydroxy-2,5,7,8-tetramethylchroman-2-carboxylic acid), 0.8% D-glucose, 2,000 U ml^−1^ catalase and 165 U ml^−1^ glucose oxidase. For the torsional spring measurements, 30% sucrose was added and the final NaCl concentration was lowered to 1 M. Enzymes, Trolox and glucose were purchased from Sigma-Aldrich. Finally, the sample chamber was filled completely with FMB 1.5 and a custom-made plug that secures 0.2-mm-thick platinum wires, to which the operating voltage is applied, was attached on the top of the flow chamber. The applied voltage was controlled by a custom-built LabVIEW routine that supplied control voltages to a custom-built operational amplifier to generate the final output voltage. Videos were acquired for 40 to 64 s at a frame rate of 250 frames s^−1^ with an applied uniaxial AC field of 0–60 V and frequencies of 1–100 Hz.

### TIRF microscopy video processing

Moving particles were manually localized, Gauss fitted and picked with the Picasso software^49^. All successive steps were performed using a custom MATLAB script. From the tracking of the position of the rotor arm tips, the cumulative angular displacement was obtained. Furthermore, angular velocities (*Ω*) were calculated according to:

$$\varOmega =\,\frac{{{\vartheta }}_{{\rm{last}}}-{{\vartheta }}_{{\rm{first}}}}{\triangle t}$$

in which *ϑ* is the angle at the respective frame (first and last frame of a period with or without the external AC field) and ∆*t* indicates the time difference between those two frames. A histogram of the angular velocities was calculated.

### TIRF microscopy experiments for DNA-PAINT

For DNA-PAINT super-resolution imaging, all three corners of the triangular platform were labelled with three transient DNA-PAINT binding sites. After rotor diffusion data were acquired, the FMB 1.5 imaging buffer including oxygen scavenging system was exchanged with DNA-PAINT imaging solution consisting of 1×TAE, 12 mM MgCl_2_, 0.05% TWEEN20 and 20 nM P1 imager strands. Before acquisition of DNA-PAINT data, rotor fluorophores were bleached by increased exposure to the 642-nm excitation. Videos were recorded for 7,000 frames with 400-ms exposure and a 642-nm excitation laser output of 70 mW. The spot detection of imager binding events and Gauss fitting of point spread functions was performed with the ‘Localize’ function of the Picasso software package. Subsequently, the ‘Render’ function was used to visualize the resulting event list and correlate the DNA-PAINT super-resolution data with the data of rotor diffusion measurements.

### Langevin dynamics simulation

For the simulation, we view the rotor arm as a Brownian particle in a time-dependent 1D energy landscape *U*(*ϑ*, *t)*. This allows us to write the first-order equation

$$\lambda \frac{{\rm{d}}{\vartheta }}{{\rm{d}}t}=-\frac{{\partial }U({\vartheta })}{{\partial }{\vartheta }}+\,\eta (t)$$

with damping constant *λ* and noise term *η* that satisfies $\left\langle \eta \left(t\right)\eta \left({t}^{{\prime} }\right)\right\rangle =$ $2{k}_{{\rm{B}}}T\lambda \delta \left(t-{t}^{{\prime} }\right)$. The energy landscape consists of a time-independent rotor-intrinsic contribution and an alternating external electric field

$$U\left({\vartheta },t\right)=\,\frac{4a}{\pi }{\rm{\cos }}\left({\vartheta }\right)E\left(t\right)-b\sum _{n}{\rm{\exp }}\left(-c{\left({\vartheta }-{{\vartheta }}_{0}-n\triangle {\vartheta }\right)}^{2}\right)$$

in which

$$E\left(t\right)=\left\{\begin{array}{cc}1, & \left(t\,{\rm{mod}}\,T\right) < \frac{T}{2}\\ -1, & \left(t\,{\rm{mod}}\,T\right)\ge \frac{T}{2}\end{array}\right.$$

represents the alternating external electric field with oscillation period *T*. Parameters *a*, *b* and *c* denote the relative strengths of the electric field, the intrinsic rotor landscape and the width of the local energy minima, respectively. Furthermore, *ϑ*_0_ describes the angle enclosed between the rotor and the field axis and the energy minima are placed apart by Δ*ϑ*. To ensure the link back to the rotational dynamics, Δ*ϑ* must be a simple fraction of 2π. To enhance the numerical stability, we work with a differentiable energy landscape and, hence, we approximate for some large *N*:

$$E\left(t\right)\approx \,\mathop{\sum }\limits_{n=1}^{N}\frac{{\rm{\sin }}\left(\frac{nt}{T}\right)}{n}$$

### Statistical drift analysis

The irreversibility analysis described in the main text and illustrated in Fig. 4 is conducted on data in the following manner. Given a time interval Δ*t*, one computes all pairs $\triangle {{\vartheta }}_{t}={{\vartheta }}_{n+t}-{{\vartheta }}_{n}$ from a time series {*ϑ*_*n*_}, in which this *ϑ*_*n*_ measures the angular position of the rotor including previous full rotations.

From these data, we use a kernel density estimation with Gaussian kernels (https://github.com/JuliaStats/KernelDensity.jl) of the probability distribution $p\left({{\vartheta }}_{0}\,{\rm{mod}}\,360,\triangle {\vartheta }\right)$, representing a jump from position *ϑ*_0_ to *ϑ*_0_ + Δ*ϑ*. Using this distribution, we compute

$$\frac{\triangle s}{{k}_{{\rm{B}}}}=\,{\left\langle {\rm{\log }}\left[\frac{p\left({{\vartheta }}_{0}+\Delta {\vartheta },t=nT| {{\vartheta }}_{0,}0\right)}{p\left({{\vartheta }}_{0},t| {{\vartheta }}_{0}+\Delta {\vartheta },0\right)}\right]\right\rangle }_{{\vartheta }}$$

by averaging over initial positions ${{\vartheta }}_{0}\in [0,360)$. Analysis of each rotor *k* yields a function ${\left(\frac{\triangle s}{{k}_{{\rm{B}}}}\right)}_{k}$ that follows an approximately linear trend independent of *t*. Estimated distributions over these functions are shown in Fig. 4 for simulated and experimental rotors. The average slope of the resulting trend is proportional to the rotational bias. To account for the notable variation in rotational bias in different experimental motors, all trends were renormalized to a unit slope. This renormalization step becomes ill-defined for unbiased experimental motors which were thus excluded.

## Online content

Any methods, additional references, Nature Research reporting summaries, source data, extended data, supplementary information, acknowledgements, peer review information; details of author contributions and competing interests; and statements of data and code availability are available at 10.1038/s41586-022-04910-y.

## Supplementary information

Supplementary Information This file contains Supplementary Figs 1–4, the uncropped gel scans, uncropped TEM micrographs, the custom scaffold sequence used for the pedestal and Supplementary Tables 1–5.

Video 1 Exemplary single motor particle trajectory, AC field first off, then on (blue trace, Fig. 3a). Left, raw video of an exemplary single particle. Yellow cross indicates tracked position of rotor arm tip. The particle effectively rotates in CW direction when switching the AC field on. Right, cumulative angular displacement of rotor tip. The original video was acquired with 250 frames per second. Total frames collected: 10,082; (real) video time: 40.328 s. Every second frame of original image stack was exported.

Video 2 Exemplary single motor particle trajectory, AC field first off, then on (orange trace, Fig. 3a). Left, raw video of an exemplary single particle. Yellow cross indicates tracked position of rotor arm tip. The particle effectively rotates in CCW direction when switching the AC field on. Right, cumulative angular displacement of rotor tip. The original video was acquired with 250 frames per second. Total frames collected: 13,538; (real) video time: 54.152 s. Every second frame of original image stack was exported.

Video 3 Exemplary single motor particle trajectory seen during stepwise AC field axis rotation (black trace, Fig. 3c). Left, raw video of an exemplary single particle. Yellow cross indicates tracked position of rotor arm tip. The particle’s angular velocity depends on the direction of the AC field axis relative to the motor body. The AC field axis was rotated from 0° to 180° in 5° increments. Right, cumulative angular displacement. The original video was acquired with 250 frames per second. Total frames collected: 16,000; (real) video time: 64 s. Every second frame of original image stack was exported.

Video 4 Exemplary single motor particle trajectory seen during stepwise AC field axis rotation (blue trace, Fig. 3c). Left, raw video of an exemplary single particle. Yellow cross indicates tracked position of rotor arm tip. The particle’s angular velocity depends on the direction of the AC field axis relative to the motor body. The AC field axis was rotated from 0° to 180° in 5° increments. Right, cumulative angular displacement. The original video was acquired with 250 frames per second. Total frames collected: 16,000; (real) video time: 64 s. Every second frame of original image stack was exported.

Video 5 Exemplary single motor particle trajectory seen during AC frequency sweep (black trace, Fig. 3e). Left, raw video of an exemplary single particle. Yellow cross indicates tracked position of rotor arm tip. The AC frequency was reduced in a stepwise fashion (100 Hz, 10 Hz, 5 Hz, 1 Hz). Right, cumulative angular displacement. The original video was acquired with 250 frames per second. Total frames collected: 16,000; (real) video time: 64 s. Every second frame of original image stack was exported.

Video 6 Exemplary single motor particle trajectory seen during AC frequency sweep (blue trace, Fig. 3e). Left, raw video of an exemplary single particle. Yellow cross indicates tracked position of rotor arm tip. The AC frequency was reduced in a stepwise fashion (100 Hz, 10 Hz, 5 Hz, 1 Hz). Right, cumulative angular displacement. The original video was acquired with 250 frames per second. Total frames collected: 16,000 (real); video time: 64 s. Every second frame of original image stack was exported.

Video 7 Exemplary single motor particle trajectory seen during AC voltage sweep (black trace, Fig. 3f). Left, raw video of an exemplary single particle. Yellow cross indicates tracked position of rotor arm tip. The AC voltage was increased stepwise from 0 V to 60 V and then shut off again. Right, cumulative angular displacement. The original video was acquired with 250 frames per second. Total frames collected: 12,000; (real) video time: 48 s. Every second frame of original image stack was exported.

Video 8 Exemplary single motor particle trajectory seen during AC voltage sweep (blue trace, Fig. 3f). Left, raw video of an exemplary single particle. Yellow cross indicates tracked position of rotor arm tip. The AC voltage was increased stepwise from 0 V to 60 V and then shut off again. Right, cumulative angular displacement. The original video was acquired with 250 frames per second. Total frames collected: 12,000; (real) video time: 48 s. Every second frame of original image stack was exported.

Video 9 Illustration of expected motor behaviour as a function of AC field axis orientation. Left: top, assumed free energy profile of an idealized motor featuring two energetic minima; middle, the energetic contribution of the external AC field; bottom, effective free-energy profile (plotted in polar coordinates). Green line gives rotor arm orientation. Middle, free-energy profiles plotted for Cartesian coordinates. Green dot gives rotor arm orientation. Right, cumulative angular displacement as seen during a Langevin simulation. In this simulation, the AC field axis was held stationary for a fixed number of frames and the particle dynamics was recorded; then the AC field axis was updated to a different orientation as indicated by arrows.

Video 10 Illustration of expected motor behaviour as a function of AC field frequency. Left: top, assumed free-energy profile of an idealized motor featuring two energetic minima; middle, the energetic contribution of the external AC field; bottom, effective free-energy profile (plotted in polar coordinates). Green line gives rotor arm orientation. Middle, free-energy profiles plotted for Cartesian coordinates. Green dot gives rotor arm orientation. Right, cumulative angular displacement as seen during a Langevin simulation. In this simulation, the AC field frequency was held stationary for a fixed number of frames and the particle dynamics was recorded; then the AC field frequency was increased by the AC field frequency factor indicated.

Video 11 Illustration of expected motor behaviour as a function of AC field amplitude. Left: top, assumed free-energy profile of an idealized motor featuring two energetic minima; middle, the energetic contribution of the external AC field; bottom, effective free-energy profile (plotted in polar coordinates). Green line gives rotor arm orientation. Middle, free-energy profiles plotted for Cartesian coordinates. Green dot gives rotor arm orientation. Right, cumulative angular displacement as seen during a Langevin simulation. In this simulation, the AC field amplitude was held stationary for a fixed number of frames and the particle dynamics was recorded; then the AC field amplitude was increased by the AC field strength factor indicated.

Video 12 Exemplary single motor particle trajectory with a torsion spring during one winding cycle (bottom-left trace, Fig. 4g). Left, raw video of an exemplary rotor with torsional spring during one cycle of winding (AC field on) and unwinding (AC field off) with superimposed tracking position of the rotor arm (yellow cross). Right, corresponding cumulative angular displacement. The original video was acquired with 250 frames per second. Total frames collected: 14,000; (real) video time: 56 s. Every second frame of original image stack was exported.

Video 13 Exemplary single motor particle trajectory with a torsion spring during two winding cycles (top-right trace, Fig. 4g). Left, raw video of an exemplary rotor with torsional spring during two cycles of winding (AC field on) and unwinding (AC field off) with superimposed tracking position of the rotor arm (yellow cross). Right, corresponding cumulative angular displacement. The original video was acquired with 250 frames per second. Total frames collected: 14,000; (real) video time: 56 s. Every second frame of original image stack was exported.

## Data Availability

The cryo-EM map that is shown in Fig. 2 is available in the Electron Microscopy Data Bank (EMDB) under the accession code EMD-14358. Cryo-EM and real-time fluorescence video raw data are available from the corresponding author on request. Sequences of oligos and scaffolds as well as source data are available in the Supplementary Information file. Source data are provided with this paper.

## Source data

Source Data Fig. 3
Source Data Fig. 4
Source Data Extended Data Fig. 9

## Code availability

The MATLAB scripts for TIRF microscopy data analysis can be downloaded from https://github.com/DietzlabTUM/matlab_tirfm_movies^50^.
