# Supplementary material for: A DNA origami rotary ratchet motor
Source: Nature. 2022 Jul 20;607(7919):492–8. doi: 10.1038/s41586-022-04910-y (PMC9300469; doi:10.1038/s41586-022-04910-y)
Supplement: Supplementary file 1 — This file contains Supplementary Figs 1–4, the uncropped gel scans, uncropped TEM micrographs, the custom scaffold sequence used for the pedestal and Supplementary Tables 1–5. [file 41586_2022_4910_MOESM1_ESM.pdf]

nature portfolio

https://doi.org/10.1038/s41586-022-04910-y

Supplementary information

A DNA origami rotary ratchet motor

In the format provided by the authors and unedited

A DNA origami rotary ratchet motor

Anna-Katharina Pumm[1], Wouter Engelen[1], Enzo Kopperger[2], Jonas Isensee[3], Matthias Vogt[2], Viktorija Kozina[1], Massimo Kube[1], Maximilian N. Honemann[1], Eva Bertosin[1], Martin Langecker[2], Ramin Golestanian[3, 4], Friedrich C. Simmel[2], Hendrik Dietz[1]

[1]Lehrstuhl für Biomolekulare Nanotechnologie, Physik Department, Technische Universität München, Garching near Munich, Germany

[2]Lehrstuhl für Physik Synthetischer Biosysteme, Physik Department, Technische Universität München, Garching near Munich, Germany

[3]Max Planck Institute for Dynamics and Self-Organization, Göttingen, Germany

[4]Rudolf Peierls Centre for Theoretical Physics, University of Oxford, OX1 3PU, Oxford, UK

Supplementary Information

Contents:

Supplementary Figures S1-S4

Supplementary Data

- Uncropped gel scans
- Uncropped TEM micrographs
- Custom scaffold sequence used for the pedestal
- Table 1-5

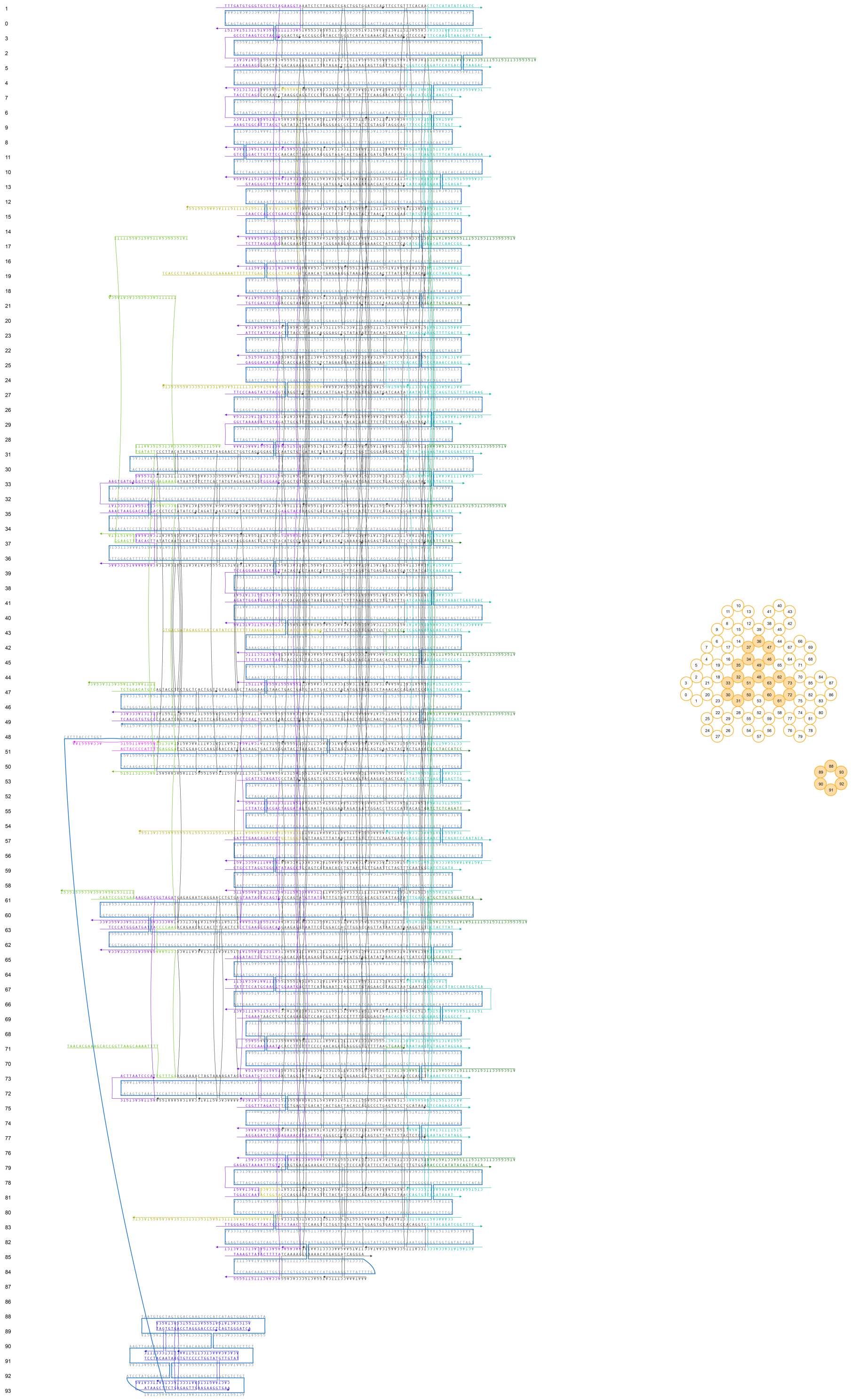

Figure S 1 Strand diagram (left) and bottom cross section (right) of the pedestal. Made with caDNAno v0.2. Connecting oligos to the triangular platform are depicted in light green. Oligos for Biotin anchors are colored dark green

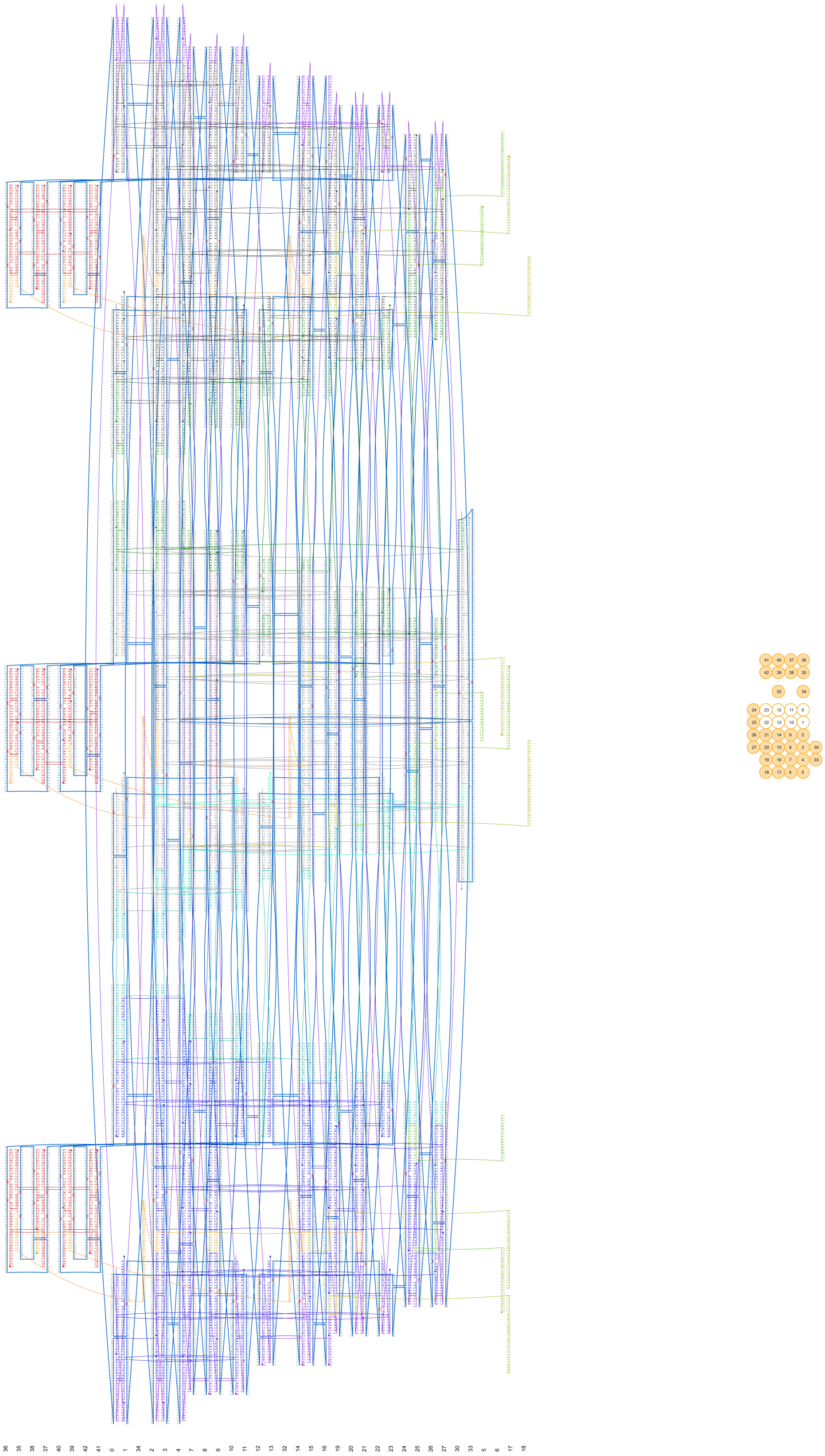

Figure S 2 Strand diagram (left) and bottom cross section (right) of the triangular platform. Made with caDNAno v0.2. Connecting oligos to the pedestal are depicted in light green.

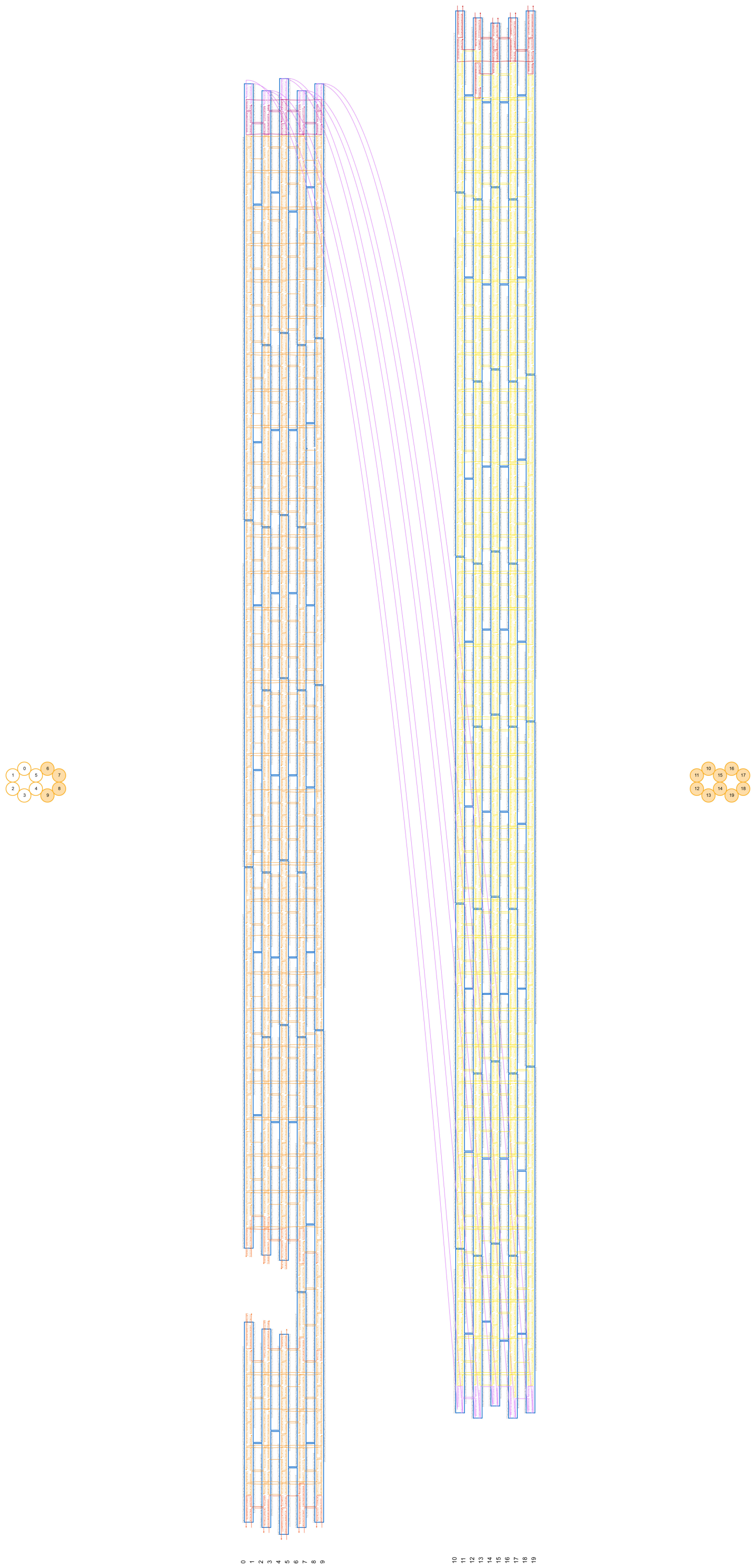

Figure S 3 Strand diagrams (middle) and bottom cross sections (outside) of the rotor arm dimer (left: first part attached to the stator, right: rotor arm extension). Made with caDNAno v0.2.

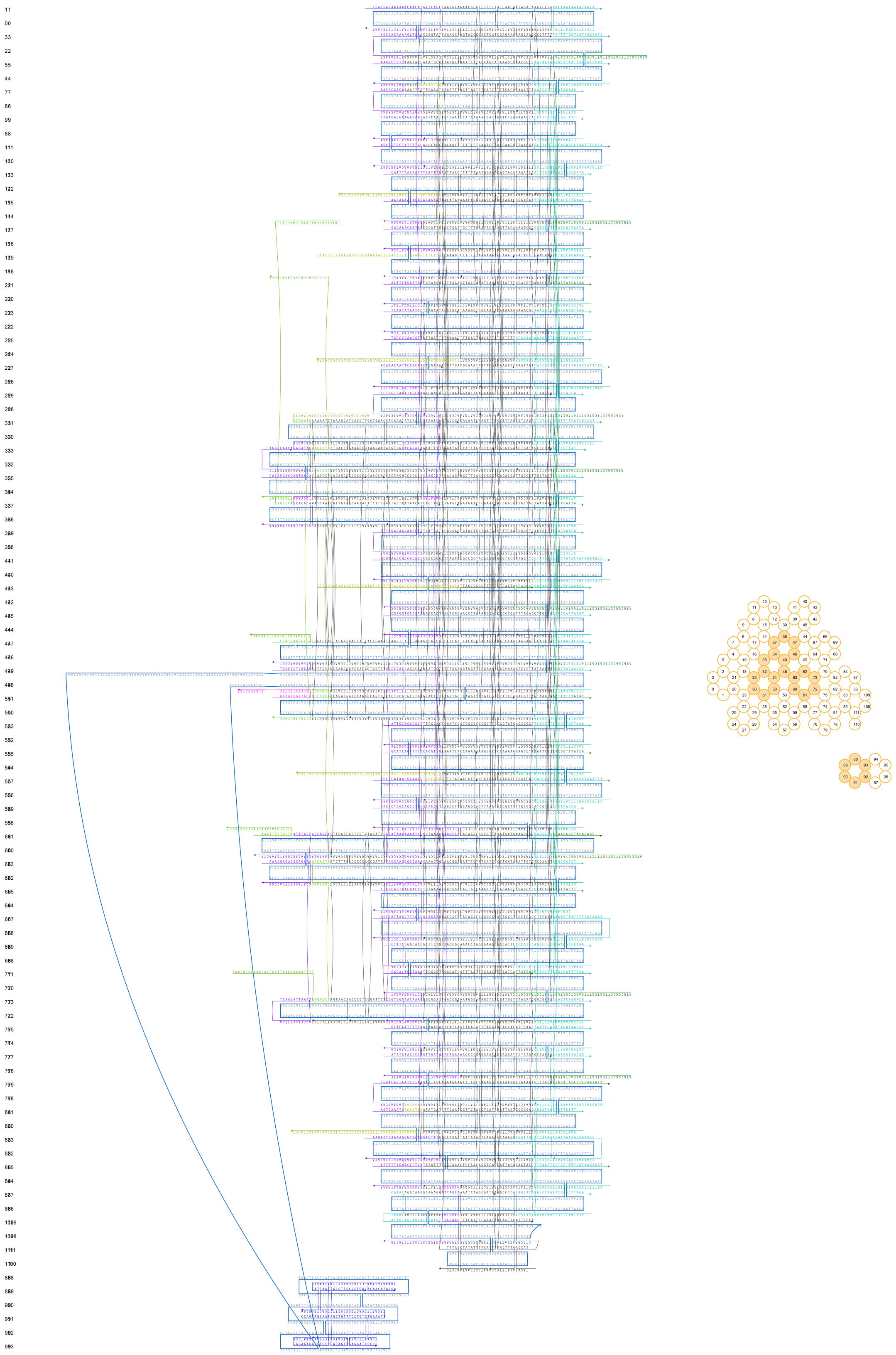

Figure S 4 Strand diagram of the pedestal (left) and bottom cross section (right) with torsional spring. Made with caDNAno v0.2.

Supplementary Data

Uncropped gel scans

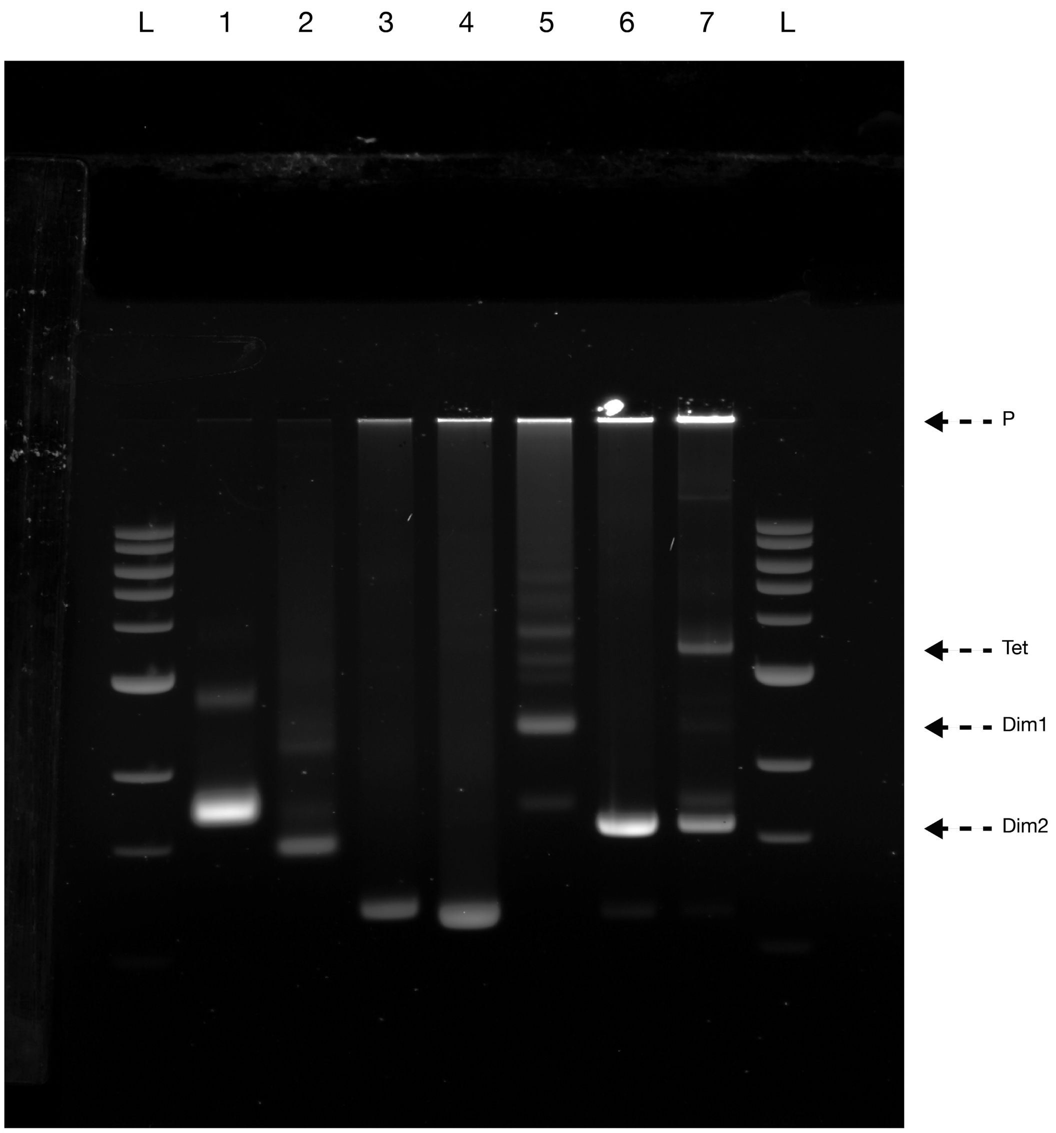

Supplementary Data Figure 1 Uncropped gel scan corresponding to Extended Data Figure 3

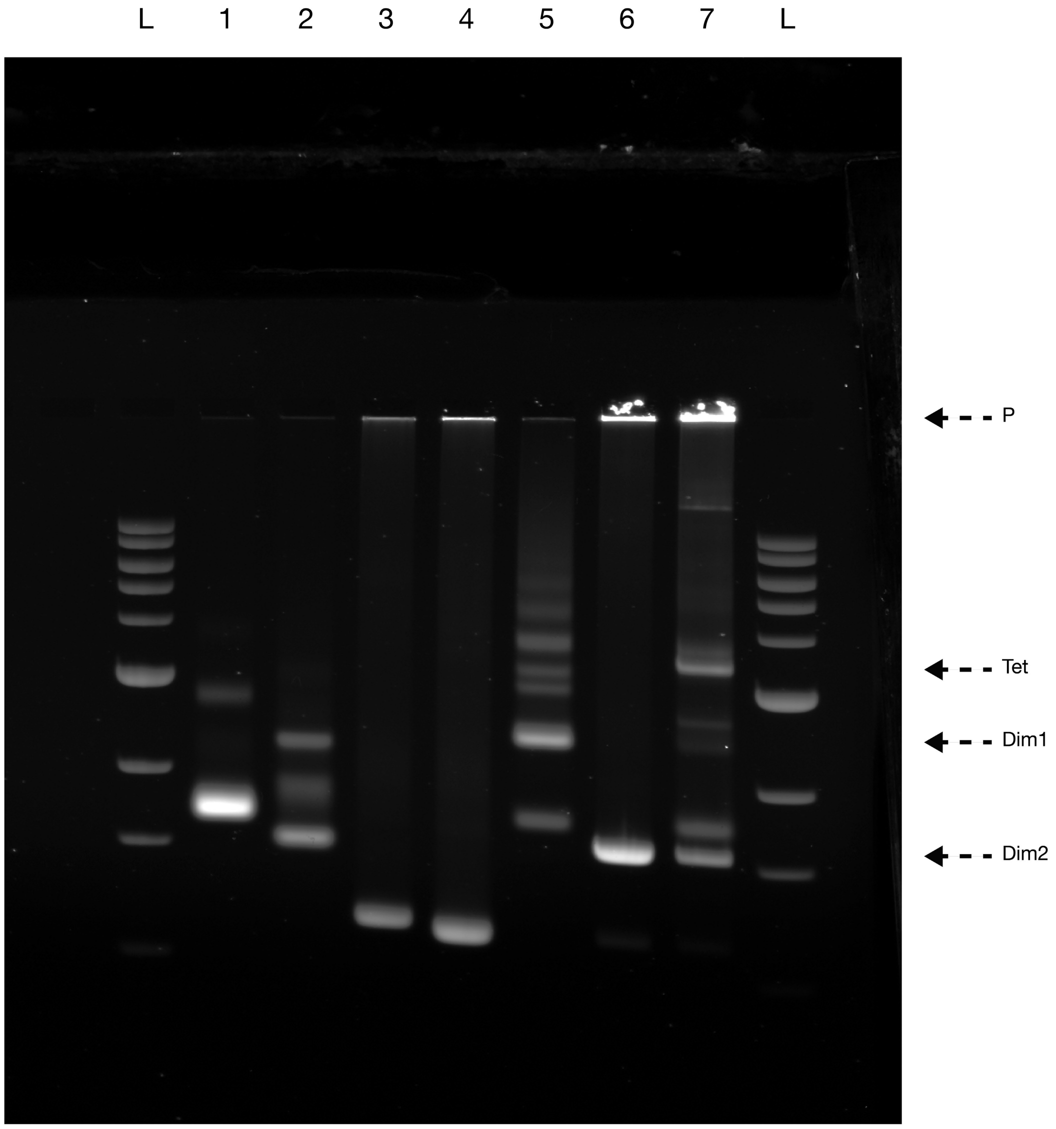

Supplementary Data Figure 2 Uncropped gel scan corresponding to Extended Data Figure 12

Uncropped TEM micrographs

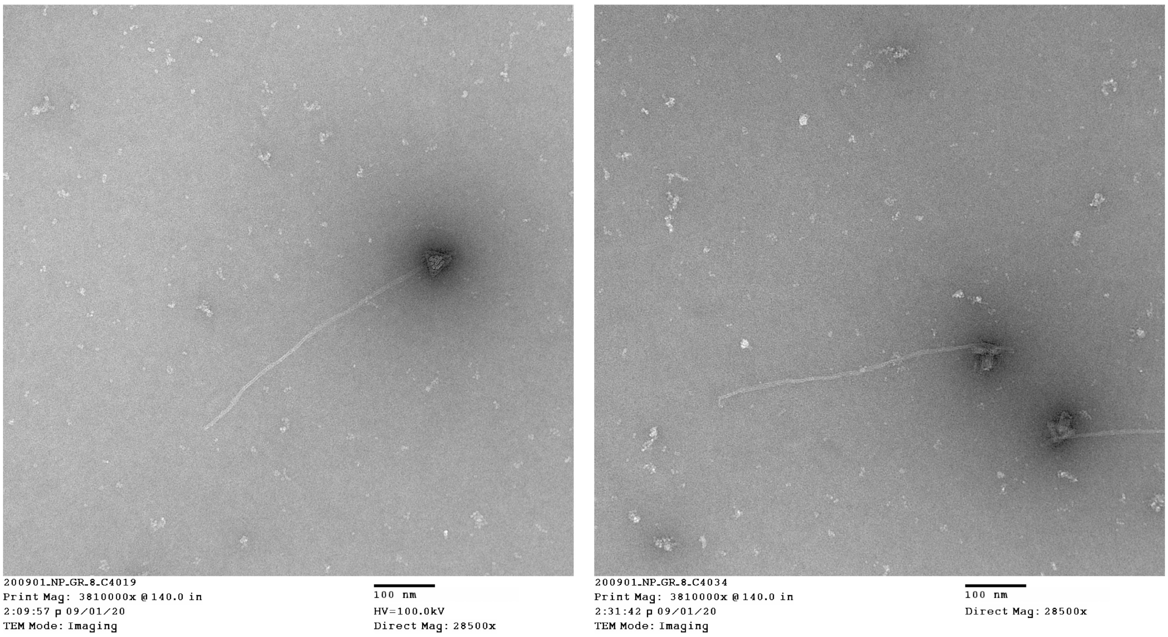

Supplementary Data Figure 3 Uncropped TEM micrographs corresponding to Figure 2

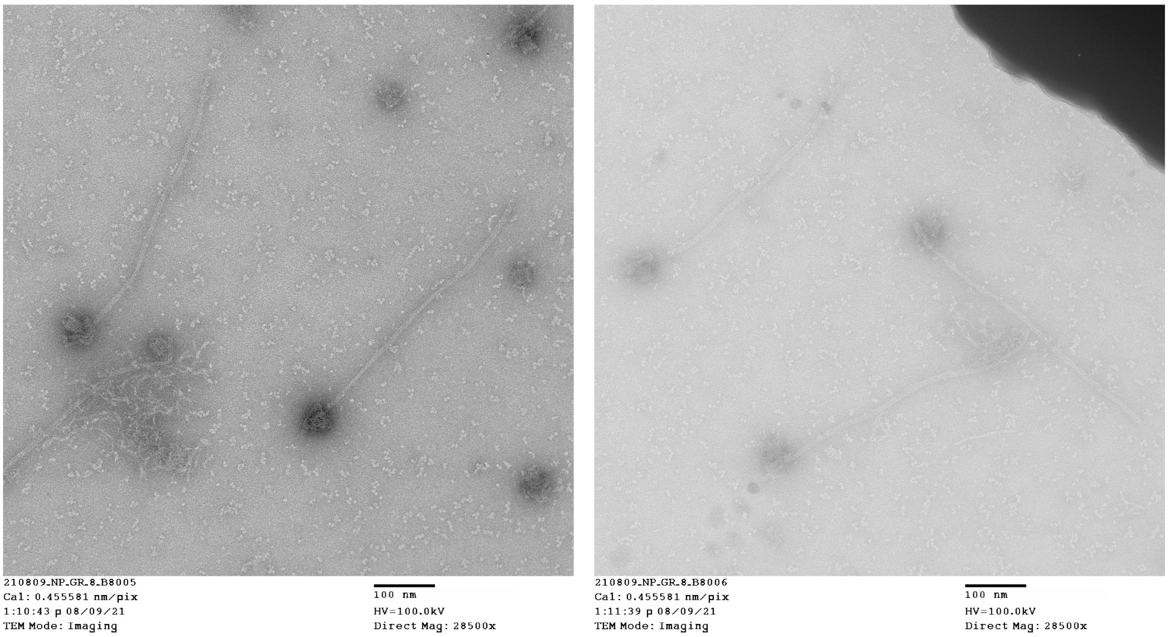

Supplementary Data Figure 4 Uncropped TEM micrographs corresponding to Extended Data Figure 12

Custom scaffold sequence used for the pedestal

AGCTTATATCCTATGGAATGAGATTGTAGGACTCAAAAGTTGAAAGGGAGAGGT
TAACAAGGAGGGGTCCCTAGGTCACACTAGGGTATAATGTGCTAGTGGACCAAG
TCCCATCATAGTGGAGTATGTAGGGGATGATCCCACTGAGTTTGTGTGTCTTCTC
CTGAATACAACATACCAGGGGACACTTGCAGGGATTGAGACTTAGTGTCTGTACT
GGTTCACCTTCTTCAACTCTCAGACATTTACCCTGGTGACCAATTCCCTAGAGAT
AGACTGTTAAATGATGAATCAGTCTATGATAAGACCTTCCCAAGAGAAGGTTGA
TAGTCTTAGGTATCCCTAGTCACTTGTGGATGGTTGCTTGGGTTCCACATCCCTCA
AAATGGGGTAGTCACAAGAGGGTTACTCTTGTCTAAACCCCACTCAAACCTTAAA
GGAAGATTTCCTAGACTACCAGATATTACGTATTTTCTGAGTTCTTACCCTTACCA
GTCCCAAACCCTCAATGATATGTGAGTTGTCTTGTACTTGGTCAGGACCGACTCC
TATATAGGGATCTACAATGCCCTAGAAGGATAAGTCTCTGGTACTCACCTAGAAA
CTAATTCTGAACTCAACTCCCGTTCTTTGGTTTGTGGGGTTCATTAGTTTGGTCGT
CTATCACTTGAGAAGACAAGAGTTATAAACTTAACCACCCAGCAGGATCTGTTCA
AATCACTAGGCTAAATTCCCCACCTAAGGCAGGAATCCTTGACAGAACTGAACT
GGTTTGAGAATTGCATTGGGAAAAGAATTTTAACCTAATGACGTGTGGAAAACT
ACAAATCATAACATACTGGACACTGTAGTATTACTCACAGGTTCCTGATTCTCTC
ATCTACCCATCCTTTTCACCGGATTGGGTGCCTGGTCAAGGGGATCATCCCATGG
GATGGTGAGGGATGCTTCTTTCAGGGGTAATGTTAGAAATGTACACATACCTATA
GAATGAGTCTGAAATTCAGAAGGAAGTTATCAGTTAAAACCCCTGATATTTAGTT
ATGAGGATGAGTTGGTTAATATACTACATCAAGTGTCACCTCTGACTGTGTCTGA
ACAGGAGTATCCTAAGATGGTATTAAACTTGCATGGAAATAAGTGAAATCAACA
TCAGGTAGTAGTGAACTAAACCAGACTTCATCAATTATCAATACTCGTACAGGGA
CAACTTCCCAGGACATGTGTTTACTCCCCAAAAGGGTAACCGTTGGACCTTCTGG
ACAGGTTATTTCACCTCCTTCTTGGAGTCATCTGACTCAGTGCAGATGTAGGTAG
GAACAATGACCACGTCCGAAAGGAGTGTTCACTGGATTGTACAATCACACCGTT
CTGATACAGAGTCTAATACCTAGTTGGAGGACATTCATGTATCTTTTACTAGTTTT
CCTTCAAACAATGGGATTAAGTGACAGTGTAACTTCCATTTCTGATTGGATAACT
GTGTTTTGTGTATGGGAAACACAGAAGATCTAaaccGGTTTGTTACCCCTGTACCA
CACTTCATGATGGTTTGGGGAGAGTTTACTAACCAGTCTGGAGAGTAGAATTAAC
ACTGTGAGCGAGGGCCCTGTAGTTAGGTTTCTCCTAGATCTCCTACTGGTGGTGG
GGTGGAACAAATTTTACTCTCACTTAGTAAGGTGGACATCGAAACCACTGGCAGT
CTAAGCCCCACGTCTGTTTGACTGTCTGGGGAACAACACTGGTTAGACTTATGGT
CTGGTGGATAGTAGAACTAATCTCCTGGGTACCAGTATTGGTCCAGTGTCCTCTG
TTAGGAGTAAGCTACTCCCAAAGAGTGAGAGTGTCAGTCTGTCTGTTGTCCCTTT
TGATAAAAGTATAACTTTACCCCAACAAAGTTGGTCCTGTGGGCAGTCCATGAAA
GGTTTATTTTGTTCCCTGATCCTCATGTTTTATTGAGGGGTTCTTCATAAGGTATT
GACTTGGCAAATGGAGGGTGGTGATACTAGTGAAACCGATCTGTAAGGACCTGT
GGAACTCACACTCCATAAGTCAACCAGAACTTGAAAGTTAGAGGTCTGGGACGA
GTGGGGACAGGGCACACCGGTTTTCAGTGTGTAGGGAGTAAACTGTTTGGATTTA
TCCTCTATTTTATCCACAGTGTGACTGTATATGGGGTTTCCACAAAGTCAGTAGG
AATGATGGGAGACCAAGGTCTTCTGTCACTTTATAGGTCCTTTGTTCACCGATTA
CAGGAATGTACCAAGGGGTACATCTACTAGGTCCCCTATAGTATTCCCAGATTAG
AAAACACATGGCTCTGGACTTTATGCAGACACTCAGGGCCTGGTGTAGTCAGTG
ATGTCAACTCAGGCCCTTGTACACTGTTGATCAGGAACCTCCATGTTCTGAGGTT
CTCTTGCACCAGGGTTTAAGGGAGTTTAAGAATGAACTCCTGACTTCCTATCTAC
ACTTATTTACTTCACTTAAAACACCCCTCATCTGTTGGGGAAAGAAGGTGTATTT
TGAGGCCTTCTCCAAAGAAATCTAAGAAAATAGGATGCCCAGTGAAGTGTGAGG
TCTCAGATACCCAGGCCCAATCTTCTCAAGACATCACCATTGGTAAGTGTGGGGA

TTCATTACCTAGGTTCTACAAACCTAGATTCTATGAAAGTCATTCCACCCCACCC
ATGATCAGATAATTGATAGAATCTGAAAGGATAGATACCATTACATGGTACTAA
GTTGGCAATACCAATAAGTAGGACACCTTTATGATTATAACTGGTCCAAGTGGTC
CGGGAATTATCTCTTCTTGTCCCCTTCAGGAGAGTGAAAGTGGTGTTCTGTCTTG
GGGTACTGGGGTTATGGAGGTATGACTCAATCAGATTTCACATCCATATTGTGAT
ATGGGAGCCTAGACAATTACCCTACACCAGAGGAGATCATGACAACTAGACCAA
TATCATGAATCCCACAAGCATGGTCAAATATGATGACAGTCCCTATACTATCAGA
TCCCATTGAAACtagaATTCAACAGTTACAGGTGTTATGACTGCAGGCTATATCATC
TGGTAGGTGGTGTACTTCTGTATTGATGTTGGTAGGGTACTCTCCATCAGTGGGT
CCTTATTACTATGTATTGGGTCTGGACCCTGAAATCTGAGAGATCACTGTAATGG
GAAGGTCCAATCATCTATTCCCCTAATTCACTATCCTAGTCGTCAGAGGAAATCA
GTGACACTACTGATTCAGCCTGTTCACGGTCGGTCAGAGTCGTCTGAGAACCCAC
AACTCACTTGGAAGGATGGTAGGAGGTTCAGTAGTACATTCACTGTGTACTCCCT
ACCTGATTTCATTCCTATGTACATTAGACCCCATGAGACAACTGGAGGATTGAAA
AGCAGTGGGGTCATGATCTTGTTGGGTCCAACTGGGATTCTGGTGTTTAGACCAG
ACCATGTAGGAGTCAATACATCAGTCAGTTACTCCTACCCTCCAAGTCTTGGTGA
TGGACTGAATAAGGTGATTTCTAGTGATCTCCATACAGATGCGAGGGGAACCTAT
TAAGAGTAAATCCATAGACAGTACTCCATCTCCGAGGGAACAGGGATCCAACGA
CAAAGGAGACCTGCAAAGATACCTCATTCTATCCTCGGTTGTATCCCCTGTGAAA
GGCACTATATCCATCTTGGACAGTCAGGTTTTGTCACTCAGTTTAGGTCAGTTGG
GGTGTCTGGATGATAGATCATCTCTCTCACACCTGAAGCCCTGAATGGTTAGGAC
TGTAACACCTAGTGACCACCTCTAGGGAATTGGTTAGTAGTATAGGACCTTAAGT
TGTATACCTTACTACAATACACTCTGAGTATGCCCACAATCCCAGGTCTGAAGAG
ATGGAGTCTAGTGGTCACCTTGGTACTTCAGGTAAGGAGATAGGGACATTAATCT
GTGGATATAGGAGGGTCAGCCTAGGTAACTAGTACCCTATCTGTCTCCGTGTCCC
TGAGGTGTCAAAAGCTCCAACTTGGGGATCCCAACATAACCCTTAGGGGTCTGA
GGGTCCACAATAGACATAATTGTAAAATTCCAGGACTCCCATTACTTCTATAACA
TGACCTCTCCCAACCACAAACCATATTAAGTATGACACATTGGGTTACACATGAT
CCACAAGTGAGTCAAGTTCACTAATTTCAAGAGACTCAAGTTGTCAGGTATCAGA
CACATCTTAGTCTGACCCTTGTCAAACCACCTGGAACATATTATATTGATCATCA
CAACTATAGTTCAATGGGTAAAGAACCTGGAGGGGACCCTTTTCTGTACCCATGT
GGTTACCTACTCTTAAGGGTACAGGTCTAGTTCCTTGGTTTTGGACCCCACAGGT
AGATTTAGTCAAAACTTTCCTGTAATCCTACTTGTAAATATATACAGCTCCCTGGT
TAAGGTAAGTCTGGTATGATGGAAACCAGGTAAACCCAAAGGACTCTTTGATCA
CATCAGAACCTTTTACCTCACAATCTTGATAATATAATACCCCtactTAGGGTCTGT
AGTGGATAAAGTGGGTATCTTACCTTTCTCAATGTTGACCAGTAAGGGGTGAGAT
AGTTTGATTTTCGGATTCCCTCCCCAGATCAAACCCATATTCTATAGGGTCAACC
CTTTAACCGGTTGATGTCCTAGGACATATCTCCCATAGAAAATCCACACATAGTT
CTGAAGGTTAAGTACTTAACATAGGTTCCCTCTAAGGGTTCAGGCATATAGGTGT
TGGTTCTGTGGTCAGAATCACTCCAAGAAATGGATCTAAAGTGATCGAAAGGGT
TAATCTCACAACACCACCCCTGGTCCCTGTGTCATGAAACACTGAACCCCAATGT
TACATCATGTCAGTGTCTACCCTTGCTTTAAGTGTTGGAAACAAGTCCAGTCACA
TAATGTACTCGGAAGTCCAAAGTGAGGAAACTTTAGAAAGTTTCTCTTCAATTTA
ACAATGTCACCAAGAACTACTTGGACTTGAGTAACTTATGCCTTGAGTCTTAATG
TAGGCATGAGTCGTTACCTTGGAAATGGGAGGTCATTGTGTTTCATATGACCCAG
GTATGGCCGGTGCAGTCCCCGAAAAGGTCATCCGGTCTCAAGTGGGGCCATGAC
TTAGAGTAAGAGTCCTGGTGGGATTGGAACCCAGACTGATATATGAGAGTTGTG
AAACAGGAACTGGATCCACCAGTCGACCTAAGAGATTTACCTTCTACAGACACC
CACATCAAAACAGTACAGAACATGCTCTAGGACTTAGGGCAGTGTATCCACCCT
CGTCCCCACACAAAGGGATAACAGACAATCTCCACCTTCCACTTATCTGTAGGAT

CAGAGATTGGTCATGGATCCGGGACCCACACCAATCAACTGTTACCGAAGTCTAT
AGATCCTCTCTGTCATAGTCCCCTCTTGTGTGAGAGAAATTCCTCATCCCTTGTTC
CATTGGGGTCTATATGTTCATATTACTGACTCCTCAGTTGTTGCATGTTTGGGATG
TTCTTGAAATAAATGACTCTCAAGGGACCTGCCTTAGGTTGGGGCTGAGGTAGGT
AATGATCTCATATCTTGTCAGTTCATCTAATTGTGTCTCAACCATGAATATGTGTT
CCGTGACTAAGGGGAAACTGCCTACCTACAGATAAGGGGCTCCCTCTGATCAAT
ATATCACCTAAATGCCACTTTTGTTGGACTCTCTAACATGGTCCTTGATGGGTGG
GAACTGTGACCATAGTGTTAAGGAACCAAAAGTACCATATGGTGATTCCTTTGAT
GGATTGGTGTCGTCTTCTTCCCGTCCATCCACTAGTGTAATAATAGAACCCCTAC
CACCAAATGGGTTGTTTCTTCAGGCCTCTATCCCACCCTTGATCCCCATAATTTT
AGAGGACAAACTCTGGCTCATGTGAAGATAGGTTTTCTGGGTCTTTCCCATATAA
GACTTCGTTCCTTCCTAAAGAAAACTGTACTCAAATAATCCACCGACAGGAAATT
GGGTACAAGGAAATACTCTgtcgGATATCAATGAGTACATAAATACCTCTTAGAGG
GAATCAATTCTTAAGATAGATGTCTACGGTCCAGACTCGACATGATGTCTTGACT
GAGTGTGAATAGAATACACGTAACAGGGGTCAATTAGAGTTCACCCCAGAGTTG
GGTTGACTGGATGTGGAATGGGTGTCAGAGACTTCTCTCTGGATTTGTTCTAGAC
AGAGGTCGGTGGTTTATGTCCCTCAGATCTACTTTGGTCACGTAGATACTTGGGA
ATCGAGGTAGACAGGGGATATGGTTACATAGGAAGTGACCTCAACCTCTTGGTT
CCATGGGGAATTCCCATCTACATCTGGGAGGAGAAGATTGTGTAGTTCTACTTCC
AAAACGCAATCTACAGTGTTTTAGCCTTTAGTTTACCCAAACTCCCCTGACCAGG
TTCTTATAACACTCATATGTAAGGGAATATCACTCACTCCGAGAAGAGAAAAGA
CTCCTTGGGGCCCTTTATTCAGAGAGAGGACTCGATCATGTTATCTGGGGTCCTA
TTGACAGGGTGGGGAAGGAACATTCTACTGTATCCTGGGAGTCAGGAATTCATA
CTTTAAGGTCCGGTGGGACAGCTGGTTCCCACCATTCTCTACATAGTGAAGAGGA
TTATCTTTCTTCCAGACCTCATCACTTATAGGGGAATCCACGGTGTCCTTAGTTTT
AGACATCCTTCTGTGAATGTCTGGTTGTGAGAATCTCAGTTCCCCATCCAAATAC
CACATCTCATCAACTATTCAGTAAGTTAGTTCATAGGGTAAGAACTTAATATGGT
CACAGGGAATGGTCCACTCTCTCTTTTCATGTGTACGGACTTGGGCATGTACAGT
GAGTTCCCTATGTTCTCAGGGGAAGTGGATTGATATAAGTGTAAACTTCCTGGTT
GGACATTTTCTTGTGAGTGATCAATGTATATCCCCAAGATACAATGGAAGATAAG
ATATTTCCTGGAATCATAGACCACATGTGGGTAAGGGTCCCATTCTGGATCTCGA
TCCTCCATTACCATGTCACAATAATCTCTTGATCAAATACAAGATGGGTTAAAGA
ATCCCCTTTACCTGTGTGGTGTGGTTCATCCAATCTCTAGATGAACATACAGGGT
ACCCCTCCCTTAAGAAAGGAACTCTACTTGAAGTTGTTCCTTACTTAGGTGGGAG
TTGTTTGATATGACAAGAAAGTAAACAGTGTCAATGGTATCCGTAAGGCATCAGT
AGAGGGTGAGTAATGAAAGAAACAAATGGTTCCTAAGTTCCTACAACCAGTGAG
CAGAAGGTACTGACATGTCCAGAGGTGGGTAGAGAGTACCAAATCCTCAGACCC
CTGCACCTGGTCTAAATTTCCTCCCCTTGGTCTTAGTAGGAGAGACTACTCCTTAT
GCAACCTAGGGGTGGGTGTGGATTCTAGTTGTGGAAGTTCTAACCCTCCCAAGTG
AAGGGTTGGATAGAGTGGAGGAGTCCACTGAAATGGTAACTGATGTGGGGCACA
CGTTGA

Table 1 Oligo Sequences used for the pedestal

core_01	GGCATTCCACATCCGTAGGATCCTTCCCGACTCCCCCCTAAG
core_02	GTGGATCGTGAATTATTAGTTGGTTAAGTTTATAA
core_03	AGAACCTTAAAGGGCCCCAAGTCTTCACATAGGGTCAGATTA
core_04	TTTACCCATTGAATCACTTCGTAGAACCTCACTTAATATGG
core_05	AGACCCCTGAGAGTACAATTAAGGGAGCCTCACTT
core_06	GGAGTCCGGAAATCGACTAGGGGTCTTACTATCCA
core_07	GACCCCAGACCTTACCCCAAGCCACTAGTACTGAATACACAT
core_08	ACCTCAGTTACCTGTGGTATTCACTGTAGTTATCTTCCATTG
core_09	GACAGAGAGGATCTCCTTTGTACATCTAATCATACCAGACCA
core_10	TATCTCCGGACACGCACAAGTTTCCTTTAAGGTTT
core_11	ATAATCCGAGTCTTATATGAGTGTTATAGAGTGGG
core_12	AGATTCTCCCCTGAGGGATATACATTGATCTGCTCTGAGGAT
core_13	TAGTTGAACTTGGGCCTTCTCTAATTCCGCTCCCA
core_14	ACTAGTTTGTGGAAGTTTAGACAAGAGTCCCTTACTTCTCTT
core_15	GAGACAGTATGTAGAGAATGGCTCTGAAGGTCAGG
core_16	TTCCCTATTCAGGGTCCAGAAGGGGATTAACCGAG
core_17	AACTCTATTTGGAACTATGTAACCATATTGAGGTTC
core_18	TGTGAGAATACTACTGGTCTAGCATAAGGTATATT
core_19	CATGGTAGTATTTGAGTGCCTTTCACAGGGATCCCAACAACT
core_20	GTGATCATTCACAACAGGACTCTTACTCCAATGAC
core_21	CTTTAACCCATCTTATGGAGGCATTGACTCACCTT
core_22	GATAGAATGAGGCCACCACACAGGTAAATGGGACCCTACTGACAA GACT
core_23	CTTTGGGTTTACAAAGTCAACTCTTCTCCCAAGAGGTTGAGG
core_24	GACCTGGTTCTTACGACCATTAGGTCCTGAGATGA
core_25	AACACTTAAAGCAATGGACTTAGAGGGAAGGGTGGAAGTCCG
core_26	CCCACCTCGGATACATCGAGACTTCAGGGAGTGAT
core_27	AAGTGGATTGGTGTGAGTCAGACATCCCACACATA
core_28	TTTACCTACTGGTGTGGCCCCACTTGAGATACCTGTGTTATCATAGA CT
core_29	CCATCACTGCCTTAAAGTAAGTCTCCTTTGTCGTTGGGATAC
core_30	AATTATGGACCCAGTTGAGACCATTTATTTCAAGATAATATGTACC CAC
core_31	GAGGTGGTGTATTGACTAAGATCAGAGGCTGAATTATTAGAC
core_32	AGTACCTTCACTCAATATCAATCCACTTCACAACCAGTTACC
core_33	GTAGGAATATCTTGGAACATAGGGAACTTGGATGG
core_34	CTTAGGATTTAGACCAGGTGCTGGACTCGGAACTGATGTCCC
core_35	TTGGTACCCACATCAGACATTCCCTCCTATATCCA
core_36	GAGGAAAACGTAACTGACTGATCACTAGCATGCCC
core_37	GAGTAGTGAACTTCACTAACTACTCCATTCTGGGG
core_38	TAGGCAGAGAGAAAAACATTGCTTTTGGTTCCTTAAGACGACTTTC TTG
core_39	AACAGTCACAGAACACCTCCAGAGAGAATCAGGAAAGTTATC

core_40	GTCTAATAGAATCCACACCCATATTAAGGATTGTGCCCTATAACTACAG
core_41	TCTGTACTCATTGATTTATCCGAATATGATCTTCA
core_42	ATTGCGTATTGACCCCACCGACCTCTGT
core_43	TATCCGAAATTCCTCACCCTGTTGGGAGATTAGTG
core_44	CCCAAGCATCATTTATTTCAGAGGGGTCACTGGTT
core_45	GATACCTTTTTGACCAGCTGTATTTCCTACCGTAGGTGGGGAGGACTGC
core_46	AAGACTATATCTGGAGGAGTCGTGTCACGCAGTCACACCACCTACCAGA
core_47	GGAGTACTTGGGATAAGTATGCAGAGTAAATTGAT
core_48	TAGTCTAAATGTGTTGATCGACTTTACCTTAACCA
core_49	TACGTAATCGGTAGAATCAAAAGGGTTACTCTCCTACTCCTA
core_50	AAGAACTACAAGACCGTGAACGTTGAATACCAACA
core_51	GGTAAGAAACATATTCGGTAACAGTTGAAGGTGGATCCCTCT
core_52	ACCAAGTCAGAAAATTTGTGGTCAATAGGTATATA
core_53	ATCAGTAGGTCCTGTGTCTAGCGGACCACTTCCTTTGACACT
core_54	TGAGAAAAGGGAATTGGGAAAGGGATCAACCTATGTCTGACCGATGGAC
core_55	TTGGACCAGAACGGCTTCTCAAGTGATAGTACCCT
core_56	TTCCCATGACCGACAACTCACTCTCCTCAATCATA
core_57	TCAACATGTACCCACCCACCGGATAACACATACTT
core_58	AAAACCTGGTTTGACTCTTCAGGTTATGACAGTGA
core_59	AGAAGTATAACACCTGCAATTATTTGTAATCAACATCTGTAT
core_60	TCAATACCTCTTGTGAGTTGAGTTCAGAAGGGGAA
core_61	GATATATTGATCAGGATGAACTCTTATACCGAAAAAAGGTGATTGGAGC
core_62	TCTAGTTCAGTTAAAATTCTTCAGTGTTCATTCCT
core_63	CTATAGTCATGGGTACAGAAACTAGAACTGGGGTGGGGAGCT
core_64	TACACAACCAACTCAAATCCAGAGAGAAGTAACCATGTGATG
core_65	TTCCCAATGTAACTAGGCTGATAGATGAAACTTGA
core_66	CTGTCATTCAATGGCGACTCTTACAGTGTTGAGTCATGTAGA
core_67	CTGCCAGAGTTTGTTTCAGAAAGATCCAACCAATC
core_68	CCTCTAAGAGAGTGCCTATGACACAACTGTACATA
core_69	CGAGGGTGGACTATTGAGGAACCCAACC
core_70	CAGTTATTGGTGTAACACGTCATTAGTACTCAGAATGTACAA
core_71	CATTACCAGGAAAACAATCAGAAATGGA
core_72	GTTTTAAGTGTGATCATGGAGGGCCCTGTAAACTCGACCATAACAGACG
core_73	ATCAGGGAACCAACTATCTATTAGGTAAAGTATTG
core_74	GTGTACAAGGGCTGTCCAGTATATCACACCTATAT
core_75	TCATCCTCCCACCCCTAGGTTAACACCACTAGAAA
core_76	TCAATTAAGAATCTCTGGTTTAGTTCACGGTCCAATTCTTTG

core_77	CTTTCATTCTGATCTTTCCCCACCTACAGGAAAACATGAGGACTTTCAT
core_78	AGGTTTGATTCAGTCATGGTCTAACCAAGAAAAGA
core_79	TAGAACCCCTTTCAGTTTTAATCGGACG
core_80	GATAGAGAACGAAGTGACAAGTAAGGCAGGTCCCTAATGGAA
core_81	ATAATTGGGGAGTAGGGCATCCTATTTTGAGGGGTGATTCTATGATGTA
core_82	CTGTACGTGAATCCGAGATCAGAATCCCCAACTTACCCTGTG
core_83	GAGAAGGCCTCAAATAACCTGTCCAGAATACTACC
core_84	TTAAGTAAAGTTTCCCCTTATCTGTAGGTTCATGG
core_85	GGGAAGAACACTATCACTGACATGATGTCTTTCTACTTAACC
core_86	CACCTTCATGGGTGGACACAGCCAAGGGACCCTTC
core_87	GGGTAGAGGTCACAGTTCCCAACTAGTGACAGAACCTAACCA
core_88	ATCAATACATGGAACTCCCAGTCTTGAAAGGTCATTAAGGGTATGTACT
core_89	GAACAACTTCAAGTTCACCCTCTTACCCTACAGTC
core_90	TGTTCCTAACAGATCTTAGATCGGTTACCCTTTTGATGAAGT
core_91	TGGTCATCAGAACGCTGATAACTTGGACGGAATGA
core_92	ACCGGCCACCGGATAATCTCTTAGGTCGGGTTTCCTCTTAAGTTTCCTT
core_93	CTAGTAAAAGATACGTGTACATCACTCTCTGATTCAACCATC
core_94	TCCAGCTACTAAATAAGGTGTTCATGGGACTGAACCTCAGACAGGATAC
core_95	AAAACACCCTGTGATCTGATTGAGTCATACCACTTTTTCTAA
core_96	GGAGTGTCTGAAAACCGGTGTTCCACCATCCCCAATCGCTCA
core_97	ACTGTTTACTTTTAATTGTGATCTATCATCACTTT
core_98	GATTGTCGGTCATATGAAACATAAGTCAGATCCAG
core_99	TCCTGAGTTGACATTGAAGTGGATTAGTCCAGTGG
core_100	ACCATCACACTGACGAACCCCTCAATAC
core_101	TGGTTAGAGTGTCTGCCAAGTCAATACC
core_102	TGGGGCTCTCCCATGTAATCGGTGAACAAGGGCCC
core_103	TTCCTGTAAGAGTCAAGAGGTATTTAAATACAGATCTCCCAT
core_104	CATTCCTACTGACTCAGTCAAAGTCTAACTACACA
core_105	TTGTGGATGTACCCCTTGGTAAATTCTACTCTCGG
core_106	TTTCGATCCAGTGACAGAAGACCTTGGTTAGACTGTCTACTAGCCCTGT
core_107	AAATAAACTCAGGGAACA
core_108	GAGTTCCACAGGTCTCCATTTGCATAAA
core_109	GGACTGCCCACAGGTCAAAAGTCTGCACACTAGGTTCAGACTGAAGAGATTGGGAA
core_110	TTCAAGTCCCCACTCGTCCCACCCAGGATGGTACA
core_111	TCTGGTTGACTTATTTATGAATACACCAGTTCCTGGTTTTCCGGGTAAT
top_01	GGAATGATGGAGGGTAGGACATTTGTTTT

top_02	TTTCTTATCCACGACTAGGATAATGTGTA
top_03	TTTCACAAGAGGGGATACACTTTTTTTGCCCTAAGTCCTAGGG
top_04	ATATGAGTTTAGGTCATTATGTGACTAACATTT
top_05	TTTAAAGTGGCAATCATTACCTTTTTTTACCTCAGCTTTCTCTCATTT
top_06	TTTGTCCGGACTTGTTTCCAGGACCATGTTAGAGATTT
top_07	TTTGTAGGGGTTCTATTATTACCCCATCA
top_08	CTCCACTTCATAGACCTGAAGTGTGAAAGTAATACCAAGGATTCTTT
top_09	TTTCAACCCAGCCTGAACCCTTCCGAGTA
top_10	TTTTCTTTAGGAAGGGCCTGAAGAAAATTT
top_11	TTTGATTTGAACAGATCCTTGGAATTTAGCCTAGTTTT
top_12	TTTTGTCGAGTCTGGGTCGGTGGATTATTT
top_13	TTTATTCTATTCACAGTCCTCTTGGGAAC
top_14	TTTGAGGGACATAAACCTGTTACGTGTTTT
top_15	TTTTTGGGAGTAGCTTACTCCCTCTAACTAACAGAC
top_16	TTTGCATTGTAGATCATATGGAGGGACAA
top_17	AGAAACCCAGTTCAATAGCCTTGATTTCCTCTGTTCTAGGTTT
top_18	TTTTTCCCAAGTATCTACGCCCCTGTACTGTAGACTTGGG
top_19	TAAACTAAATTTTTTGGCTAAAACCTACCTCGATTT
top_20	CTCGGAGGGTCTGGCTGTGGATTCCCCTATTTTTTTAAGTGATGA
top_21	TTTAAACTAAGGACACCGACACAGAATACACTTCAAGAAAATGTCC AACTTT
top_22	TGAGATGAAGTACCTCAAACTATCTCACAGTTTTTT
top_23	ACATGTGGAACCACCTGTATGTTCATCTAGTTT
top_24	TTTAGATTGGATGTCTATGATTTTTTTTCCAGGAAATATCTGT
top_25	TGTTATGCTCAAACTAACTACAAGGACCTATAAACCCCACCACCAG TTTT
top_26	TTTTTCTTTCATTACAGAGTTCCTTTCTTT
top_27	TTTTGGACCAATTTACTAAGTTTTTTTGAGAGTAAAATTTGTT
top_28	TTTAGGAGATCTAGGGGGGTAACAAACTTT
top_29	TGTTTCCTCCTCCATGAGTCAGATGATTT
top_30	TTTTGAAAGGAGGTTTTTTCTCCAAGAAATA
top_31	TTTTAAAGTTATCACTCTCACTCTTTT
top_32	AAGGATGAGTTACACTGTCTTTTTTACTTAATCCCAT
top_33	TTTCTGCCTTAGGTGGGATGTTCTGTTACAGTG
top_34	TTTCGGTTTAGATCTAGACTGAACTTTTAACCAACTTTGTTGGGGTTT
top_35	TTTTCCCATGGGATGATTAGAAGCATCCCTCACCATTT
top_36	GGTAGATTAACCCCAGCCCCTTGACCAGGCACCTTT
top_37	TTTTCAACGTGTGCCTCTCTACCCACCTTT
top_38	TTTTTTGATGTGGGTGTCTGTAGTCAAGACATCATTT
top_39	CATTCTACTGTTCAGGTTTAATACCATCTTTTT
top_40	TTTTATTTCCATGCAAGGTTGATGTTGATTTCACTTTT
top_41	TTTAGGATACTCTAGGTATATGAATGCATACAC
top_42	GAAGGTAGACCTTTTCAGCATGTTCTGTACTGTTTT

top_43	TTTACTACCCCATTTAGGGAATTGGTCACCAGGGTAA
bottom_01	TTTGTATAGGGAGAATACTATAGGTTT
bottom_02	TTCCAAGATCCCACCTCTCATATATCAGTCTTT
bottom_03	TTTTGGGTTCCAGTAACGACTCATTTT
bottom_04	TTTTCAAGGCATAAGTTACTACACCCTAAGTAGGTTT
bottom_05	TTTAAGTAGTAGGACATCAACCGGTTT
bottom_06	AACTGAGGGGTCCCGGATCCATGACCTTAAGACTTT
bottom_07	TTTCGCATCTGTATGCCACACTGTACCATTGTAGATAGGAATTT
bottom_08	TTTGACATTGTTTGGATTTTCTATTTT
bottom_09	TCGGAGATGGATATATCAAGAGAGACCTAAACTGAGTGACTTT
bottom_10	TTTAACCCTGGTGCAGTCCAGAGCCATTTT
bottom_11	TTTCCAGGGGTGGTGTTCACCAGGAATGTGAGATTTT
bottom_12	TTTGGGTATCTGAGACCTCACAAAATAAGGTAATGG
bottom_13	CATCAAATATGGTAGGGTTCAGTGTTTCATGACACAGGGATTT
bottom_14	TTTTAACCCTTTCGATCCAGACACTTT
bottom_15	CTTCACTAACACATGTCCTGGGAAGTTGGGCCTTTT
bottom_16	CTATGTGAAATTGATTTCCCCTTTCTTGGTTTT
bottom_17	TTTACTAGTATCACCACCCCTTACAGATCGGTTTCTTT
bottom_18	TACCAATGGTGATTTTTTTGTCTTGAGAAGATTGTCC
bottom_19	TTTTGGTATTTATAAACTCCCTTATTT
bottom_20	TTTGTGTTTTCTAATTGGATAAATTTT
bottom_21	TTTAAAGGTTCTGATAAGTTTTGACTATTT
bottom_22	TTTTAGTAATAAGGACCCACTGATGGAGA
bottom_23	GACGACCAAACTCCAGACCCAATACATTT
bottom_24	CATGAAGTCACGGAAAACATGCACAAGTCCTTT
bottom_25	TTTTTAAAGGGTTGAGGCATACTCTTT
bottom_26	TTTGGTCAGACTAAGATGTGGGAATTCCCTAATATGACCCTTA
bottom_27	TTTTGGGTTCTCAGAGATCTGATATTT
bottom_28	TTTTTCCAAGGTTTGGGACTGGGTTATAGGAATGTTTACAGGA
bottom_29	TTTCTGTGGATAAAATAGAGTTCCCCAGA
bottom_30	TTTCCTCCAGTTGTCCCTACTTATTTT
bottom_31	TTTGGTATTATATTAAGATGTCTATTT
bottom_32	AGAGAACTTTGACCGTCATGAATATCATTGAGGTGAGTTGTTT
bottom_33	TTTAAAACCTGACTGTCCAAGATGGAGTACTGTCTTT
bottom_34	TTTCCCAACTTTATAGGTTCCCCTTTT
bottom_35	TTTCCAAACAGTTTACTCCCCAGTGTCTCAGAC
bottom_36	TTTTAAGGTATAAGTTGGACCCAATTT
bottom_37	TTTAGAGTGTCACTGCTTTTCAATTTT
bottom_38	TTTAATCTACCTGTGTGTCTGATATTT
bottom_39	AGAGTAGGTCTCTGACACCGTCCAAAACCAAGGTTT
bottom_40	TTTAACTAGACCTGTTTCCAGGTGGTTTGACAAGTTT
bottom_41	TTTGGAATTTTACAATTTAAAGTAATGGGAGTCCTTTT
bottom_42	TTTTCAGGGTAATGAACCCCACAAACCAA

connection_to_triangle_01	AGGGTCCCCTCCACCAAAGTAGATCTTTTTGACGATCCATCGCCCAGGGCGCTG
connection_to_triangle_02	GCTGGGTTCTAGGTGAGTACCAGAGATTTTGATCGGTCCGCGGTGCGGGGAACGCCACTGGC
connection_to_triangle_03	AAGTTTGAGCCCGCCCACTCGTGCAATTTTGATATTAACCCTCTTGTGTTT
connection_to_triangle_04	TTTCAATCCGGTGAATTTTCGTAGACAGCACCGCGTGCGT
connection_to_triangle_05	GTCCACCACTGGTAGACTAACAGAGGACACTTTCTGAGTCGCTCTCTCGCAACAGAGGTACCTC
connection_to_triangle_06	GTGACGATAGAGGTCATCATATCCTTTTTTAAGGGAGGGGTATATCTTTGCAGG
connection_to_triangle_07	TAACACGAAAGCACCGGTTAAGCAAAATTTTTGTTTGACCTGAAACCCCAAGTATCTCT
connection_to_triangle_08	TTTTCTGGACATGTCTTTTAACATTAGCACTCACGACC
connection_to_triangle_09	CAACACCTATATTTTGGTGTTTTGTTTACAAGCGGTGGC
connection_to_triangle_10	TCACCCTTAGATACGTGCGAAAAATTTTTTGAGTACCCCTTACTGGCAAGGGA
connection_to_triangle_11	TATGCGGCGATTGAGTCAGGTTTTTGGAAGTTGGATGTCTATTT
connection_to_triangle_12	TGAGGGAACCTAGGAAGAAAGTTTTTTCACCGCGCCACATAGCA
for_biotin_anchor_01	ATGCGGCCTTCGTCGTTTGCCTACAAATCTCTGATCCGATTGTGAGGTATTT
for_biotin_anchor_02	ATGCGGCCTTCGTCGTTTGGACCTAGTAGAAACCCCATATACAGTCACATTT
for_biotin_anchor_03	ATGCGGCCTTCGTCGTTTTATGGATTTACTCTCTTGTCATATCATGTTCCC
for_biotin_anchor_04	ATGCGGCCTTCGTCGTTTGTCAGGAGTTCATTTGAACACTCCTTGTGAAGT
for_biotin_anchor_05	ATGCGGCCTTCGTCGTTTCCTGACAACATCTCTCAGATTTTT
for_biotin_anchor_06	ATGCGGCCTTCGTCGTTTTGATATTGGTCTAGTTATGCTTGTGGGATTCATTT
for_biotin_anchor_07	ATGCGGCCTTCGTCGTTTCAAGATCATGACCAGCCAACTTTT
for_biotin_anchor_08	ATGCGGCCTTCGTCGTTTGGGAGATATGTCACATTGTAGTTT
for_biotin_anchor_09	ATGCGGCCTTCGTCGTTTTTGTGGACCCTCCTACCATCCTTT
rotor_arm_dock_01	ACTCCACTATGCAGTGGGATCA
rotor_arm_dock_02	ATGGGACTTGGTCCAGAGTTGAATCCCTGCCTCAT
rotor_arm_dock_03	TCCATAGTTTATAAGCTTCTGACTAGCATAGCCCTTTC
rotor_arm_dock_04	GTGTCCCCTGGTATAAGTCTCAAGAAGGTGAA
rotor_arm_dock_05	CACTGTTGTATACACACAAACTCCTTGTTAAC
rotor_arm_dock_06	TCCTACAATAACTCTTGTGACCTAGGGACCCCT

Table 2 Oligo Sequences used for the triangular platform

side1_01	ATGTTGAATACTCTGGGTGAGCAAAAACAGGACCCAAC
side1_02	TATCATTTCTCCCCAACAAAGAAGTTAGCTG
side1_03	CGCAATAAACAGGAATCGGGGAATAAGGGCGACACGGAA
side1_04	CGGAGTTGAACAGTGTGCGCAAGTAAGAGTTT
side1_05	GGCAAAATGCCGCAAAAAAAAGAACCCGGCAT
side1_06	AAAAGAGGCAGAGCGACCCCGTTCTGGTCATG
side1_07	ACAGGTGATTAGACGTATATTTTGGCCAATAGCACAACCACAAAGCA TCGAT
side1_08	TATTTCGTTGGCACCTATGTGGGGCGCTGTGTGCACGAACC
side1_09	TGTGGATTCCTTATCCAGCAGCCAATTACGCGACAAACCA
side1_10	CTAGACCGAAGTATTGTCTCAGCGAGATTATC
side1_11	CTGTCTTGGCATATCACGAATGATGCGTTGCCTTGAATTAGCTACGAT TCAC
side1_12	TTTCCAGACTCGACTACCATCTTCAGGCCCGTTGTA
side1_13	CCGCTGGTAGCGGTGGAAAAAGAGAGGATTAGCCCTTGAT
side1_14	TAGTTGTCGCACCATTGGCTTGGGGGCCACGCAGCCAAAG
side1_15	CCGATTTAAAGGAGCACCCGGTAAACTCACATCTTGATCCGGGCTTA
side1_16	TCCTGGCGCCTTACAACAGAAAAAAAGGATCTTCTGACGCTCGTCTTG
side1_17	AAAAAGGAGAGGATTCGGCGGTGCTCTGCTGA
side1_18	CCGCCTTTGTGAATATAAGAGATTAGGTATAGAGGACTCG
side1_19	GGTATGTAAACCGTTTACTTATTTTGTTTGCAAGGGATTT
side1_20	GATCATCTGATGTTTGATCAGCACAGTCTCGATGTACCGT
side1_21	GGGGGTGCAACACGGCTCCCCCAAGGCCAATTATGA
side1_22	CCAGTAAGCTGAAGGGGCCTACCATGTCGCGCCGGTTCGGCCCT
side1_23	CGTAGGTAATCCTTTGGCACACCTACCCCATC
side1_24	CTAAACGCGATGTTGCCGTGGAGCTTATC
side1_25	TACAGCGGAGCCAGATCCTGAGAGCTCTCCTTAAGGATCGGACAT
side1_26	TGAAGTGACCGGGATTTGAACGTTACTGCTAGGACAGAGAAACTGTCC
side1_27	GTCGCTGTGCATTTATTAACTAGTGCAGGCTTAGGGCCCAATCTGTC
side1_28	AAATTCCCTGACCGGATACGATCAATCCTGTGCGCGCCATGGATC
side1_29	TCCTAGGTAAAAGACTTCGTTGTTGACCCACCCTACGCCC
side1_30	AGACGGAATGTAGAGCAGTTCAAAGGAAGGGATAACCCAA
side1_31	AGCCAGTTACCTTCGGTTTGTACACACTTTAC
side1_32	ACGAAAACTCACGTTAAGCAGCAGCTGGTAACTTGGTAGCTAGCTGTG
side1_33	AACTATAACAACCCAGCGTCAGCTTGAAGCAA
side1_34	GACTTACAACTTATCGCTATATCAGTCTTATGGCGAGAAACGAGGTTC
side1_35	CGCTGCTTTACTGTTGGAGATACCCATCGAATGTCGGCTA
side1_36	TGGAGACTCCGGGCATCCTAGTATGTATAATCATCTTTTC
side1_37	CCGCATCCACCTATTTTGAGGCCTATTCGGATGATGCTCGTGTCCGTA
side1_38	AGTCCAACTTTGTGTCTACACAGTACGGGGAAAGCCGGCG
side1_39	TCGGCGTATACTCTCGTCAGACGATAGTGGCATCTATAGCAGTGTACA
side1_40	TACGGGGCAAGAAGAGACACGGGATGTAAGCTAATAA

side1_41	TTAATTCTCCGGGCGCGCTGCGTGTGTTTGGATCCAGTGGGCGCTAC CGTCTCA
side1_42	TTCGGATACATATTTGGGAGCCCTGATCTTCCCGCTGTTGAGATCCA GTTCGATG
side1_43	TTTGAGATTCTGGGTAAGTTAGAAATACCATAAGTT
side1_44	TTCTTCCTTTTTCAATATTTTCTGTGACATACTTT
side1_45	TGGCGGCCGTGAATTGCCGGCCGAAACATT
side1_46	AGCCCGACAGTATTACAGGGTTATTGTCTCATGAGTT
side2_01	CCCACGAAAGTTCTGACGACTGACCGTGTCTGCTCGTGCA
side2_02	AAACAAGTGTTCTTATTGACGTAGCAGCCCCGCTGTAACGCGATTGG
side2_03	CAACGGTCTTCCACATTGAGGTATTACTAAAACTGCGACT
side2_04	AAGATGCCTCCGCACTATATTCTCATATAGAC
side2_05	TCTGGGACTGCTAAGGTCGTGGTATCAAT
side2_06	GTCCTTAGGGGGCACGTGAAAACTGTACCGGTCAAGACGCAACCCGC GCGAGA
side2_07	TAGAGGTGTAAGGTTATCACAAGTTCATCGACAGTTCACACCAGTGCC
side2_08	AAGTGCCCTCCCAACGGCCGGGAGACTCGAACATGCATCA
side2_09	GTTTCTCATAGCTATCTAACTATTGCACACGGGGGTTCATTACAGCT
side2_10	GCCGTTCCCTAAATTCGAAATTGGGTCGGCATGTGCGCCT
side2_11	GCGGGCAGTCACCCCGTTTCCTCTAACCGTG
side2_12	GCCTGTGGCAGTACGAATGACATACAGTTACCCTTACGGTGATCAGAG
side2_13	CTAACGACTATGCTTGAAGTGTCATCCATGGTGGGCTTACCGTCCCAG
side2_14	CATTTCGCGGGCCAGAATAAAACCATGCGGCGTTAACTGATGGAGCGA
side2_15	GCAACGAAACTTGACAGACCTCGGAGTGAGTGGGGTGCAG
side2_16	TCCGAGTTGTTATTACCACGATAAGAGTCTAGCCATTAGG
side2_17	CGTATGGTTCCAATGCGGCAGTCGCCTGAGTGTACGATCGCTCGCCG
side2_18	GATAGTGACTCGATATGCATATGTCGATCATTCCTTATTGCGTACTTTT ATCCTGC
side2_19	CGTTACCGGAGGAGGGGATGCGACCATCCATTCACAACTTAGGTTTGG
side2_20	AAGTCAGAAACCGAGCGGATGGAGTGTTATTTGTCGCCGA
side2_21	GGCCTGGACAACTGGCCGAGGGCTGTGTCCAGGTGCT
side2_22	AGAAGGCGACGGGTTAATACATGTGTTATGCCTGCTCACCATGGGCT GATGTACAA
side2_23	TTAGTTAGGACCTCCTTATCGACATCCGGGAGGGGTTACGCCTCC
side2_24	AAACACTAAAAGTTCGCACAGCCTGTGGCCTAAATAGAAG
side2_25	ACTGACGTTACGGAGTTTGACACTCTTGATCCCTCAGA
side2_26	TATAAGGTGAAGACCCTCATGACGATAGGCACTAGC
side2_27	AGGCTGGCTGACACCTTATGCTCACCATAACA
side2_28	GGAAAGTTAGATCGGCCTGTCACGTTTTTGGCGGAAT
side2_29	TAAGGGGTCCTCACCGTCACGTGCTGATTCTA
side2_30	GAGTGGAGCGGAATCTGGTAGCTGCTGCCCAA
side2_31	GAGAGCGCGTTGGTTGTTAGGCCTGAAACCGCCGCGCTGAAATGTTTC
side2_32	ACGAGTCAGCACAGTAAGTCCGACCGGAAGGTACCCCTAG
side2_33	CTCCGCGAGTTTTCTCCCTAACTCTGGATAACGGAAAACA

side2_34	AATAGCTTATAAATGTTACCTGTTAACACCGCGCAAAGGT
side2_35	GGACGTACCCTGCCAGGTTAGGCTAAGTTCAGACACGGCT
side2_36	TGAACGGATCGGATAAACGCTCGATCCTCTAGGCTGTTTATTACGTGC TGACGAT
side2_37	TCGCGACGTGTATACGTACTCATC
side2_38	GGGAGAGATATCGTCTCCACCCAACTGGGTCGAGTGAT
side2_39	CTCCTGCATCGCAAATACGTTGACACTGCTTT
side2_40	TTGGTTCGCACTAGTGGAAGTCACTGCATATATACTT
side2_41	TTCCGATGAAGCGTACCTGGGATCGAAAGTCTCCTTGCCGAAGACTT
side2_42	AAAAGTTTTAAAATCTCAAGCGGTGCGCAAACGGCGTTT
side2_43	TTGCTTCTTACGTATGCCTCTAACCGTCAT
side2_44	CGAGCGGTGTAGCGAACAAGCCAGAGCAGATGCTCGGGGATCCAAA TATTT
side2_45	ATCCCGCGAAAAGATACTGCAGGACTTAAATGCCCACTTT
side2_46	TTTAACATGTCTAGTGTCTGTTCGTGCTATAC
side2_47	ACCTAACAATTTGAGTTGGATGTCCTTCGGGGTGATCCGTAGGCACCG GTT
side3_01	TTGGAAAACATTCTGTGGCAGCTTTCACTAGGGTCAAAGGCAGGCGTT
side3_02	ATGGCACCCGCCGCGCTTAATGTTTGGAACAA
side3_03	AACTACGATACGGGAGAGTATATAATAACTCGTCGGTGTA
side3_04	GCTAAGGTCGTGAGGCGTCAATAGAAGCTCAG
side3_05	TTTGTTAACCTAATCAAGTTTTTTGGGATAATGGGGCGA
side3_06	GGGTCTTACAAATGTAAATAAACGACT
side3_07	CCGTCTATACTCCAACGTTAAATTGGGCGGAGACAAGGT
side3_08	TACATGCCCGCATTGGATTGTCCAAGCCGTATGTGAGTCC
side3_09	GACCGAGTTGCTCTTGGAATAGTG
side3_10	ATTGCGCCCATATGCTGAACCTACCTAGGCGG
side3_11	AGCATCGGTAAGTACGTTGCAGCCTGCAAGATCACCGCCTTT
side3_12	AACCAATACTTACCGGCTGTAGGTAGTAGTAGTCGCCGTACGTGTTCC
side3_13	GTATGTGGGGCCAGCAAAAGGCCAGGAATAT
side3_14	AAGTCTGTGGAGTCTCCTTTCCGTAGATGAATCTAGAAGTGCTCATCA
side3_15	CTTGCACAGTATGCTCACGCGGGCCTCACTG
side3_16	CCCGAAAAGTGCCACTTCGGTCAGCGGAACGTATT
side3_17	TGGCCGGTTTAACGTATTCCATAGCCCCCTAAGAGCAAAAGGAATAGG
side3_18	TTGTAAGCAGCTCACGATACCTGTTGAACTCTTGCGCATA
side3_19	TCGGCCACCATTAGATGGCTCTCTTCGGAAAA
side3_20	GGCTTACCGGTCTGACAGTCCCGTTAGCGCGACCGGCGTC
side3_21	ACTAATGCATAGGGCTACTCGAGGCAAGTCGACGC
side3_22	CCATTGCACGCAGTGCTATCTGGCCCCAGTGCTG
side3_23	GTAACCACATTTTTTGAGTCCACTCTCCTGTTCCGACCACCTTGAC
side3_24	CTCGTGCGCTATTAAAGAACGTGGCAGGGCG
side3_25	TCCCCCTGGGTAGAGGTGGCGAAACCCGACAGGACCCGTAAAAAGGC
side3_26	CGGCTCTTCTTCTCAGTCTTTTCAAGTGACCTAAAAGTATCTGCGCC
side3_27	TCAGGAGGCCGGACCACTGCTACACTAGAAGA

side3_28	TTGTCAACTTCGACACGAAGCTCCCTCCCTTCTTTGTTAA
side3_29	AAATAGGGCTGTCATACAAACGATCATACAGGC
side3_30	AGTAAATTATGGTCCTGGGCACAGCAATTTTT
side3_31	GGAAGCGCGCACGGCGGCTCCGCCCGCGTTGCTGGCGTTTATATAACA
side3_32	TTTCTCATGTTAATATATCAGCTCCACCCACTACGTGAACCGTTC
side3_33	GGTCGTTCGAAGTGGTGAAGATTACACCAGGC
side3_34	GGTACGGATTTCCTAGACAAAAATATTCCTAAAAAGATACGGTCAATG
side3_35	AACATTCTGACCCGGCGTCAACACCAGCGTT
side3_36	CTCCCACATTTCCCATCAGTCAGCATCTCAGTCAGGAAAGACTAACCG
side3_37	CGACGCTCAAGTCGGTGACCAGGGTATCACCACGTGGCGC
side3_38	ATTTTGTAGGATCCACCGACCTTCACCTTCGTCAGTATTT
side3_39	ATTTCACAATTCGCCTAATCGAGAATAAAGACGGAAGGAAAGTTG
side3_40	CGGACGCAGCCCAGCAGGAGCTGGAGTAGTCA
side3_41	TGAGTAAATTTTAAATCAATCTAAAGGATCCGGGCCTAAC
side3_42	AACCATACCGCTACGTAAAAAGT
side3_43	TTTAACGACCGGGCAATAGAACTTTAGTAATTT
side3_44	AGGAGTTAGCGTGAAGCACATCGTACTAGCCTGCGACCCTTT
side3_45	TAAGTAGATAACTTTGCACCAGCCCTGAAAGGACTGTCGTTAATT
side3_46	TTGTATGAGCTCTGGGTCTGAGGTCTCAAGGTTGAC
side3_47	TTTTATCAGAATTCAGCTCTTCTAGCCTGGGGGTTAA
side3_48	ATCTTGGTCATTGCTAAGGAAGCATAACACATGGATT
corner_3.1_01	AAACTCTCAAGGATTTTTCTTAAGCATTTTTCTTTTACTTTTACGGGGTCGA
corner_3.1_02	CCAGGCAGCGGAACCCCAGACATTTTTGGGCTGGAAGCCGAGGCCCAA
corner_3.1_03	GTTGAGTGTTGTTCCAGTTCGCTAGCTTTTTTTAGAGCGCAAAATAG
corner_3.1_04	GCAAGTGTTTTTAGCGGTCACGCTGCGCGGTGCCGTATGCTAAA
corner_3.1_05	TACGGCCGGGCCGAAACGTATTGAAAAGCACTTTTTTAATCGGA
corner_3.1_06	TCTGGTGAGTTTTTACTCAACCAAGTTTCTCTTAGTTCCGCGCA
corner_3.1_07	GTTCTTGCTCCATTTTTGCTGGCAACTA
corner_3.1_08	ACGCTGATGACTCGGCAAAATCTTTTTCTTATAAATCACCGAGATAGG
corner_3.1_09	CACTTTGCCTCGGGATGGGATTGCCTTTTTTTGTGCCA
corner_3.1_10	CATTGCGACCCTAAAGAATGTATTTAGAATTTTTAATAAAC
corner_3.1_11	ACAGTATTTGGTATTTTTTTGCGCTACAGA
corner_3.1_12	AAATGAAGCTTGGTCTGACAGTTACTTTTTAATGCTTA
corner_3.1_13	AGTATAGGGACCAGTGACGGTAATTTTTTGAATGAGCG
corner_3.1_14	TCAGGCGGTCTTCACCTAGATCCTTTTTTTTAAATTAA
corner_3.1_PAINT_pos_01	TAGCACATGCCATCCGTTTTTAAGATGCTTATTGAAGCCGATGGATT
corner_3.1_PAINT_pos_02	TCCATAAGACACTTTTTGGAGGGAACCTGC
corner_3.1_PAINT_pos_03	ATCAGTGACATCCATAGTTGCCTGTTTTTCTCCCCGTCGTGTAGAT
corner_1.2_01	TTATACAGAATAACGAGTCATTTTCGGTCCAACTCAAGCAGGTTCTGG
corner_1.2_02	ATAAATAAGTTGCCTGACATTTTTTTCGAATAACTTGCAGACCTTGGA

corner_1.2_03	AAGAATTACGTTTTTGTAAATGCCATTTTTGATGGGCAGC
corner_1.2_04	GTTTGTGCGTTTTTTATCATTAAGCTTTTTGGCGTCCAAA
corner_1.2_05	CTGGCGTTTTTGGTAATTGCGGGCTGATCTTTTTTGATTGCTAGTCATGGTCTTGG
corner_1.2_06	ACTGTTCCTACGCAGTTTTTTGCAATCTGACTGCTGGCCGTTTTTCCGGAGT
corner_1.2_07	AGCAATACGGGAGGCACACACGCTTTTTTACGACGGATAGGCGAAGGC
corner_1.2_08	GGCGTTTCAGACGAATTTTTCAGGGTCCTTTTTCTGCTGTAGTAACTT
corner_1.2_09	GGTCCTATCGCTTCGAACGTTTTTTATTACGCA
corner_1.2_10	TACATAGTTCATTGTCGGGCAGTTAATCCTTTTTGCAAATAGAGTTACGTCGTTTTTAATTATCCTTTTGCAATGCAGGGATTCTT
corner_1.2_11	TACTCAAGATTTTTCTCATAGGTTTTTGATTCGGTG
corner_1.2_12	AGTGTCCTGTTTTTCGGAACCTTTTTTATTGTCTAT
corner_1.2_PAINT_pos_01	ATCCGCGTCCATGTCCACGAATATTTTTCCCTATCGTTTTTGTGACTAATCCGGGGCCGGGCCGCTCTGCGTGTACG
corner_1.2_PAINT_pos_02	GACGCCCGAATTTTTACTGGGCTAGAGCCTCTTTGGTCTTCATA
corner_1.2_PAINT_pos_03	TGTTGGCATAGAGTACATCAAACAATTTTTTAAGAAACCGTAGAAC
corner_2.3_01	CACTTACGCCTGAGAGAGATTTTTCTGTATTCTGTTAGTTGTCATACATAGGTTGT
corner_2.3_02	GCTTTATACAACTCTCGATAAGACGTTAGCATCTTACTCTGA
corner_2.3_03	TCTCAGTCATAACTGCCATAATAACCAAGTGTCGTAC
corner_2.3_04	CTTGAACGCCTGGCTTTTTATCGGGGGTCTTTTTAGCGAATTCTATAC
corner_2.3_05	ACAAACCCCCATTTTTGTTCATAGTTTTTAGGCATTGTTC
corner_2.3_06	TGCGAGCCGCGTTTTAACATTGTAGCAGCCTCTTTTTAGGATAA
corner_2.3_07	CTAGGTGCGAGGTTTTTATTATATCGCACGTTTGAATGGCCTCTTTTTTGTCGTCGTG
corner_2.3_08	CCCGCAGACTTTTTAAGCAGTCTTTTTCCGGGTGCT
corner_2.3_09	TTTTGTAATGTGTGACATAGCGAATCCCACCTCGTTTCG
corner_2.3_10	AATCCGCCTCGGGAGATCTTTTTTAAAGGACTGAAATAATCCTCACAG
corner_2.3_11	TCAAGATTGTTTTTTGATTTGGTTTTTTACGCTAAG
corner_2.3_12	GCGAATAGACCTGATCTTGAGCGCGGAATCTTCGGGCTGTTAA
corner_2.3_13	CTAAGTTTTGGCTGCGGATTGGTTTTTGCAGAAGGTCAAGCGCA
corner_2.3_14	CAATCGTTTTTTGAACGTTCGTTTTTTCAGTGGTTGCCGTTATAAGTCATTCGTCGGGAGCGCG
corner_2.3_18	GAACTTCTACTCGCGATGCAAATTTTTTTAAGTCACGTCGCTAA
corner_2.3_PAINT_pos_01	TCAAAGTATCGTTTTTAAAGAATTTTTTTGCCCAACCAGC
corner_2.3_PAINT_pos_02	GGCTACATAGTACGCATTCCATCCTGCAGACAAGGGACAC
corner_2.3_PAINT_pos_03	TCGGTGTACGAAAGAGCGAGAAGTCGACCTTTTTTGTTTATATGGGG
obstacle1_01	CGAAAACGGTCTCTAAGAATGACATCGGACGA

obstacle1_02	GGTTACCACTAAGGTCAAATACAAGAATGTGCCTACGCGAA
obstacle1_03	GGATCGCTTCCGACCACCCTGGATGAGCTCATGGGACTTTACGGTGA
obstacle1_04	GTGTTTCCAACGACCACTGGAGGGTAGC
obstacle1_05	CAAAATTAATCATCCTCCCCTTGGTGCAT
obstacle1_06	CCTCGCTTATGTTGGCTCCTCCAAGAGGT
obstacle1_07	TAGCCACGTGTAGTATACCAGGATTAA
obstacle1_08	CCCGGACAAGCTGACCAGGGGGTATGATG
obstacle1_09	TGTTAAGACGTATAGATCTAGGTCGCCC
spacer1_01	GGTAACTATCAGTGGACGAATCCACTTGCCTGAAACTAGAATGCCAC CTGACTATTCTATGAGGGGGATA
spacer1_02	AGAAAGCGAGAGCTTGTAGCCCCACAGAACTTTGACTATATCCCTAA CATGCAGCTAAACCGACTCGGCTCAATCACAGG
obstacle2_01	AGAGGACGGGCGAAATTTTAAAT
obstacle2_02	AGCGTAAACCTCCGTGCAGAACAC
obstacle2_03	ATACATTAATAATTGGTCTCTCGTGAGCAA
obstacle2_04	TTTTCTACTCGTACCTCTAATTAAACGTCGTC
obstacle2_05	TGCTATGGTCTCACGGAAGCCGTACTTCCTGAAAATCTCCAGACGA
obstacle2_06	AATATCTTGAGGTAAG
obstacle2_07	GCAGAGAGCCACAGATCCTCGTGACCACTCG
obstacle2_08	ACTAATAGCGTTAATCATCGTTCAGT
obstacle2_09	GCGCCTGCGTTCTGCGTTCTCATCGGTACCAAG
obstacle2_10	GACGTTATGTGATTAGAGTGACTATTC
spacer2_01	AGCGTTGTCGTCCTACATCGAATCCACTTGCCTGAAACTAGAATGCCA CCTGACTATCGTAACTACTTGGCAT
spacer2_02	TTGGTACAACCGAATCAGAACTTTGACTATATCCCTAACATGCAGCTA AACCGAGCGGCCATTC
obstacle3_01	AAAAGCGGTGTTGCCATAGTTTGTGAATCCGCCTCCATC
obstacle3_02	TGGTGTCACGGGCAGCACT
obstacle3_03	CAACGATCAAGGCAGTGTTATCACTCAGAAGTATGTGCAA
obstacle3_04	GTCCTCCGATCGTTGTCATGGTTATCTCGTCGTTTGGTA
obstacle3_05	GATACCGCGCGCAACGTT
obstacle3_06	GCTCACCGGCTCCAGGAAGTGGTAGAGTAAGGAAGG
obstacle3_07	GCCAGTTAGGGAAGCTCCTGCAACTTTCCCAC
obstacle3_08	ACCAGCCAGCCGTAGTTCTGGCTTCACCCATGTAGTTGGCCGCGAGT
obstacle3_09	GTCTATTAATTTAGCTCCTT
spacer3_01	CCCCTGACGAGCATCAGATCCGAATCCACTTGCCTGAAACTAGAATGC CACCTGACTATGCGCAATTTATCAGCA
spacer3_02	ATGGGAGGAAGCTCCTTGTACCAGAACTTTGACTATATCCCTAACATG CAGCTAAACCGAGATCCTTCAGCTCCGG
spacer_complement_01	ATAGTCAGGTGGCATTCTAGTTTCAGGCAAGTGGATTCG
spacer_complement_02	TCGGTTTAGCTGCATGTTAGGGATATAGTCAAAGTTCTG
corner_3.1_forPAINT_01	ATCAGTGACATCCATAGTTGCCTGATTATACATCTA
corner_3.1_forPAINT_02	TAGCACATGCCATCCGTTTTTAAGATGCTTATTGAAGCCGATGGATTT TATACATCTA

corner_3.1_forPAINT_03	TCCATAAGACACTTTTTGGAGGGATTATACATCTA
corner_3.1_forPAINT_04	CTCCCCGTCGTGTAGATAACTACGATACGGGAGAGTATATAATAACTCGTCGGTGTA
corner_1.2_forPAINT_01	TGTTGGCATAGAGTACATCAAACAATTTATACATCTA
corner_1.2_forPAINT_02	TAAGAAACCGTAGAACGACGCCCGAACTTATACATCTA
corner_1.2_forPAINT_03	ATCCGCGTCCATGTCCACGAATATTTTTCCCTATCGTTTATACATCTA
corner_1.2_forPAINT_04	ACTGGGCTAGAGCCTCTTTGGTCTTCATA
corner_1.2_forPAINT_05	GTGACTAATCCGGGGCCGGGCCGCTCTGCGTGTACG
corner_2.3_forPAINT_01	TCGGTGTACGAAAGAGCGAGAAGTCGACCTGTTATACATCTA
corner_2.3_forPAINT_02	GGCTACATAGTACGCATTCCATCCTGCAGACAAGGGACACTTATACATCTA
corner_2.3_forPAINT_03	GCCCAACCAGCTCAAAGTATCGTTTTTAAAGAATTTTTATACATCTA
corner_2.3_forPAINT_04	GTTTATATGGGGGGCCTGGACAACTGGCCGAGGGCTGTGTCCAGGTGCT
PAINT_Atto655_imager	CTAGATGTAT-Atto655

Table 3 Oligo Sequences used for the rotor arm part 1

core_01	CGAAACGGGATAACTCCCAGTCACGACGGTGCTGCCGCAACTTCTGGTG
core_02	GAATTATAAAAGGGCATATGGTTTACCAATACATAGTATGTTAACAAAG
core_03	CACCGATTATTTCACGACTTGCGGGAGCCAAAGACCACCGT
core_04	GAGATAGCTCATTTGCCGCAGCTTTCAGCCCGGAA
core_05	TAGCTCTTAAGTCCTGAACAATTTTTTCGTAAAGTCGCACTC
core_06	TACAGCGAATCGGCTGTCTTTCAGGCTGAAGGCGATGTGAGC
core_07	CCGTGGTGAAGGGACGGATCAACATAAA
core_08	TGAATATGCTTTGATATTCATTTCAATTCATTTAAACAGTACAATTAAT
core_09	TTGCGTATTTCCAGATGAGTGAGCTAACGCTCACATTCGTAAACGCGTG
core_10	GGTTTTTCCCGTTGCGCTCACTGCCCGCTTGGGCGTACTTCT
core_11	CACGGAAAAATAAGAGAATATCGCCTGTTTATCAAAGCCTCCGGCGGCC
core_12	CTACCATTTGAGGAGGAAGCAAACGGCATCAGATG
core_13	TTAAACGAACGGAGATTTGTATAATTGTCAAATCAATACATA
core_14	CGACAAATAGGCAGGGCTTAATTGAGAACAAATTCATCATAACCGACCG
core_15	CACCACCCTATTAGCAAAATTTTAGCGTCAGACTGGTGTCTGGTTTAGCAATCCCC
core_16	CTTTGAGTGCCACTACCAAGCGCGAAACTTGCTTTCGATATATACGAGG
core_17	GAGTTGCGCGAAAAGACTCCAACGTCAACACTACG
core_18	CGCCGCCCCACCCTATAGCCCCCTTATTTGAAACATAATAAGGGATAGC
core_19	ACCACCAACCGTTCCAGTAAGCGTCATAGAGCCGC
core_20	CACCAGATCAGAGCTTTTCATCGGCATTGGCTGAGGATGATAAATCGTC
core_21	GCAAAGACATTTTCATTTGGGGCATAACCTGAAGTTTATATGCA
core_22	GCATTAACATCCCAGAGCAGCGACAGAATCAAGTATAACATTCGCAAACAGTTC
core_23	TTTAGAAATGTGTAGGTAAAGAAATCACAAATTAAACAAAGG
core_24	AGAGCCGAGCATTGGAAAGCGCAGTCTCGTACTGGTGAAAGTTCAGTAC
core_25	TCAGGGACGACAATAAAAAGCCTGTTACCTTGAAAACTGGTT
core_26	ATCCGGACAATTTTAACGCCAGGGTTTCTCACCGGAAGGCTT
core_27	ATTTCTGACTTTCTAATATCCCATCCTATTTTCCGGCAAACGTAGAACG
core_28	GCCAGTAAGAAAGCCGCAGCTCAACAGTAGAGGCATTTTCGA
core_29	GCCAACATGTAATTAGGTAAAATGTTCA
core_30	ATACCAAGTTACAAACTTTAACGTCAGA
core_31	AATAACCAAAAGAAGATGATGAAACAACTGCATTAAAGAAAC

core_32	GTGCCAGGTGTAAAGCCTGGGACGAGCCTCCCCGG
core_33	GACTTTTTCATGAGGAAGTAATATTCATTAGGCTTGCAGGGA
core_34	TTAGGAGCACTAACAACTATGAAAACCGTCTAAAGCATCACC
core_35	CACGTAAAATATATAACAAAATGGTGTGTTCAGCAGACTACCATT TATC
core_36	TATCTAAACATTTGTTAAAAGTTTGAGTAACATCG
core_37	GAATAATGCTCGAAATTCCACACCTGCAGCCAGCGGCAGTGTTTT CACG
core_38	AATTGCGAATGGAACAATTTCCGTCATAAACATCC
core_39	TGACAAGAAAGCTGTTAAAAGGAGCCTTTCATCGCAGGGAACCG TTCCA
core_40	AACCGGACTGACCAACTTTGAAATCATACTGATAA
core_41	GCGGAACTGCGCGATCAGGGCGAAGAACTCAAACTTGAGGAGG CC
core_42	CCCTCAGATGACAACTTGATAAAAGGAACAACTAA
core_43	AGCATGTAGAAACCAGGGCGAGGCGAAACGGATTC
core_44	AATCAATCCATGTTTACCAGTAGGTGGAACGACGG
core_45	GAACGGGAATAAGACTGAATCCCAGCTTTCATCAA
core_46	TTAAAGCGGGGTCAGGAACCTCCGTCGAGAGGGTTCCAAAGC
core_47	CAGAATGACAGGAGGTTGAGGGAACCGC
core_48	TGGCATCGATACATGTTGATTCTGTAGCTCAACATTTTGCGGCTAT TAT
core_49	ACTAATATGCAATGCTCAGATTTAGTTTGACCATAAATGGAAGAT
core_50	TTATTTAGGGAAGGTTTTATTTCAACGAGGGTGAGAATAAGT
core_51	ATCAATATAAAAGAATTAAGACCGAGGAAACGCAA
core_52	GAAAATTCGACATTCAACCGATATTCATGCAAAAT
core_53	CCAGCAGAGCAAGCCCACCAGAAGGAGCGTGGCGATGAACCAA TAACAT
core_54	GTTGCTATTTTGCACGCTAACTTTATCCGTCAAAA
core_55	CCAGTGCGCCAGCTTCGGTGCAGCCAGCTTTCCGGTCAGGAAAC CGTAA
core_56	GGTTGTGTGCTGATTGCCGCGCGGGGTC
core_57	AAAATCCATGAAGGGTCTCGTTAACGGAACGTGCCATGCCAA
core_58	ATTTATCGCGCAGATTTAGTATCATATGATGGGTA
core_59	ACCTGAGGGATTCGCCTGATTACAGTAAATAAAGA
core_60	AATTGCGAGATAGGGGCTGTGTGAAATTTGCCCCCTGAGAGTC
core_61	TATTAAAAATCAAGGAAAGCCGCGCTGGCAAGTGT
core_62	ATCAATACCAGTGAGACGGGCCCTTATAGTTGTTCCAGTTTG
core_63	GATGATGGCAATTCGGGAGCCTAAAGCATCACGCA
core_64	CATCGAGAATCATTACCGCGCGAGGCGT
core_65	AAGCCGTGTCAGTTACAAAATCTTTACAGAGAGAATAACACGAGC
core_66	CACCAGTTGGCATGAACGCAATCGCATTAAATTTTGAGCAAG
core_67	ACGGAAAGCACCCAAAAGAAC
core_68	TTTCCAGAAGATTATTTAGCGAACCTCCTCGTAGGAACAAGC

core_69	AGCACCAATATTGACGGAAATTTGAGGGTGTCACA
core_70	TTTAGTGCGTAAAAAGACGCAGAAACAG
core_71	GCTAATGCACCGGATTACCAGACTGTTGCCCTGCGTCCAATCTCTTCTG
core_72	AAGAAGTGAGTGAATAAAGATCAAAAAATCGGCCAACGCG
core_73	TCATCATATTCCTGTGACGGGTTTTTTGGCAATAC
core_74	GGTGCCGCCCGATTACGCTGCGCGTAACAGGGCGCAGTGAGG
core_75	GGGTCGAGAACAAGAAATCGGCAAAATCAACAGCT
core_76	TCACCCAGAACGTGTCCTGTTTGATGGTGCCTGGC
core_77	AAATCTAACTGATAATGGCTATTAGTCTCGGGAGCTAAACGCAGATTCA
core_78	CAGCAGCTTGCTGGTAATATCTCTGAAA
core_79	AATATCTTTGCTGAACCTCAATGAGAGCCCATTAATGGCACA
core_80	GCCGCTTGCATAACCGAGGTGGCGAATAATAATTTAACGATCCTACAAC
core_81	TTGAGATCGAAATCAGGCGCAGACGGTCAAGAGGAGTAATCT
core_82	AATTTCTCGATTATACGAAGGCACCAACAACGGCTATCGTCA
core_83	TAGCAAGGAGGGTAGGCCGGAACGTTAATATTTTGCACAATCAT
core_84	TAATCCTGATTGTTCATAGCTGTTTCAACCCGCCG
core_85	CATTCCATTTGCCTAAGCAATAAAGCCTAATAAATGAAAAGG
core_86	AAACTTCTTGAGCCATTTGGGAATTTTACGCAAAGGTGGTCAGCTC
core_87	ATAAATCAACAGAACAGTACCTTTTACATCGGGTGTTATTTG
core_88	TAGAGCTATTATCAGATTGCCCTTCACCGGTTCCGAGTCCAC
core_89	GAAAGGAAGGGAAAAAAGAAAGGTCCACGCATAGATTAGAGCTCATTTT
core_90	CACCGTACTGCGAACGAGTAACCTGAGAGTCTGAATA
core_91	TTAAATCAGCCTAATTTGCAGAGGAACG
core_92	TCTCCAATACAGCCCTCATAGTTAGCGTTTTCACGTATCAGC
core_93	TCACCGTACCAACTTTCAACAGTTTCAGCGGAGTGAATTTTCGAACCCA
core_94	CAGGCGGGCAAACTAAATATCGCGTTTTTTAGACTTTTTAAC
core_95	TTAGGATTAGCGGGTTAGAGAAGAGGAA
core_96	ACTAAAGTCCTTTTCGGATTGCATCAAA
core_97	TAATGCCCAATTATCAGTATAAAGCTAAATCGGAAAGTAGTA
core_98	CTATCAGAATCGATCAGAAAAGCCCCAAAAACACAGGAGCAA
core_99	GAGATCTTGCCGGAGCCGGAATACTTTTGCGGGAGAAGCCAATTACCAT
core_100	GGAATTATTTTTGAAAACTAGCATGTATAGCTATCTAATAAC
core_101	TTACCAGAAAGTAAAAGCCCAATAATAATGTTAAACAACATA
core_102	ATGAAAAGAGAATTTTGAGCGCTAATATAAATAAT
core_103	CCGGAAAGGGACGATTGGTGTAGATGGGCATTAAATTAAGTT
core_104	TCAGCGTGGTGCCCGGGCCTCATTTACGTTTGTGA
core_105	TGTGATAAATTTCAGCAAGACAAAGAACCCGGGTCTATAAAG
core_106	TGAAATATTACTAGAAACAACGTAATTCTGTCCAGAAACAAC

core_107	TAGAATCCTTTTTTTAGATTT
core_108	TTTCCCTTTAAGACTGGGTTATATAACTCTTTGCTATTTGAAGGCGAAT
core_109	CCGTGAGCCTCCTCTAGCTGCGGCCAGA
core_110	CCTGTTCGGGCCGTCACTGCGCGCCTGTCCGGGTT
core_111	CGGTTTCTGCCAGCTCATGGTTGGATTACCAGGGT
core_112	GAGCGGGCTTTCCTCAGGAACGGTACGCATTAGTA
core_113	TTTTTGAGCCCTAAAACATTACGTCAATAGATAAT
core_114	GACAATACCAGTCATGGATTATTTACATATCGGCCAAATGAA
core_115	GCCATCTTTTCATACTATTTCGTGCCTTTAATAGT
core_116	TATTAAACAAGCAAATCAGATATAGAAAATTCCAA
core_117	AAAAATCAAAAAAGGCTCCTCAACTTTAAGGACGTTAACGGA
core_118	CTTTCCAGAGTTTCGTCACCAAGAGCAACGCCCACTTGCGGGACAGAGG
core_119	TTTGCTAAATCCAGGGATAGCAAGCAAACCAAAATAGCG
core_120	TCTTTGACCCCCAGTAAACAGCAACCATCACTATC
core_121	GAACCAGACCGGAAATAAGTGATTATTCAGCGTTTCTCCCTC
core_122	AATTGCTAAAATCAGGTCTTTGAGAATGACCATTAAATTCT
core_123	ACAAGAGGTCATTGCCGTTCTACCATCGAAAAACATTATGAC
core_124	TTACCGAAAACAATGAAATAGTTAAAATAGACACC
core_125	AGCCCTTTTTAAGAAAGGAAACTCCTTAAGAGCCATAAAGGT
core_126	TGCCAGTTTGAGCCAGGCAAAGCGCGATTTTTTAAGTGCATC
core_127	GGTCAGTCCAGCATCAACGGCGGATTGGATCGCACTTGGTCT
core_128	TTTAGTTAATAAGGCGTTACCAGAGCACGTTTTCAAATATAT
core_129	ACCTAAATTTAAATAATGCTGATGCAAAGCTGGTACGTTATATCGCCAT
core_130	AAAATCAACCCTTGCTTCTGTAAATCGTATAGTGA
core_131	ATGCGGCTTCGCGTGTACCGAGGAAGGGAGGCGGT
core_132	GTCATACCGGGGCTTCAGACGATCCAGCGTGCCGGGTTATCCTCACATT
core_133	TTGACGACTGAGAAGTGTTTTCGTTGTA
core_134	CACGACCAGTAATAGAATACGAAATACCATTAATTAGGATTT
core_135	CCAACCAGGAATAAGAATAAACAGAACGAAAGTAC
core_136	ACAACATTTAGGAAAGTGAATATCAAGACAGATGA
core_137	GCCTGTAAAGGAATTTCGGTCGCTGACCATCGGTTTTGAAAA
core_138	TGTACCGTCGTTTACCAGACGACGATAACCAATAGTGTATGGGAT
core_139	ACCACCCTTTGCAAAAGAAGTTTTGCCACCACCCTCTCAGAA
core_140	GCCCGAAACTGCGGCAGGAGTTGAATTTCCAGAGC
core_141	AGTCAGAGCTTTAAATGGTCAGCGAGCTCATACAGGCAAG
core_142	ATGTACCCCGGTATTGTAAGACAGTCATTCAAACAAGGAT
core_143	ACAAGAAAACCAATAAAATACGCGGTTTTGAAGCC
core_144	CGCATCGTAACCCAAAGTCAGCCTGTAGTTACCAACCCAGCT
core_145	TGGGATAAACCCGTGGGGGATTTGTAAAGCCGCCACGGGAAC
core_146	ACCAGCTTACGGCTGCGCTTTTAAACGATACATCGAACTTAA

core_147	CTTAGGTGCTGAGAAGAGTCACGCTATT
core_148	TTTTTAACCTCCGGCTTACACTTAATTA
core_149	GCACTCTGTGGTGGTCTGAGAAATCGTTTAAAACATC
core_150	CCACCGAGTAAACATAATGCGCCGCTACCACCACACCCTAAA
core_151	GATTAAAGGGACTGAGTAGATGGCCAGGGCGAGTTTGCC
core_152	TCAATCGCAGAACAGCCACGCATATCAAGGAAGGT
core_153	AAAGTACGGTAAAATACGTAAGACTAAAGTTAAAG
core_154	ACAACATGTGCCTATCGGGAAACCTGTCCGGGGAGTTAGAAC
core_155	ACGCCAAGCATTCCACAGACGTTAATAAAATGCAGACGTAAC
core_156	CATAGTAGTACAAATAAAGTTTTGTCGTAGGAATT
core_157	ATTAAGATTCGGTCCAGAACCGCCACCC
core_158	AGAGGCTTCATTTTAGGAGGTTTAGTACAGGTGTA
core_159	AAAATGTAATTCGAGCTTCTCAGAACCGGAGGGGG
core_160	GTCCAATAGACTTCCCAACAGGTCAGGAGTTTTGC
core_161	ATAAATAAAGATTAGTACCTTTAATTGCTAAAGGA
core_162	TTCATTGTATACATGGCTTTTACTCCTCAAGAGCGTAGCGCG
core_163	CTCAAATAGCAAAGGATAAGAGGTCATTGTTTTAA
core_164	AGAAAACACCCTGAATGGCTTAGAGCTT
core_165	TGTATATGATATCTGAGTACCCTCATATATTTTTTGTACCATAGCAG
core_166	TATTTAATGATAATGAACGGTAATCGTA
core_167	ATTTTTTTTGAGTTGCAGATAGCTAAAAACAGGGAATAACCC
core_168	CCATCAACAGAGAGAGCGCATTAGACGGTAGCAGCAAACAGC
core_169	TGGCCTTAGGGTAAAACTGAACACCCTGGTTTAACCAATCCA
core_170	GAGTAACGGTCACGCGACAGTATCGGCCCACCGCTGTTGGGA
core_171	TCCGTGGGAACAAGCTATTACCACAGCAGTTGGGC
core_172	ATTGCAGGGAGGTGCAGCAACCGCAAGAGGACTTGCGGTCCGG AAAAAT
core_173	AATCCGCCCACGCACGGCAGCACCGTCGATCCTCACGCTGGCCAA TAGA
core_174	GGTTGCGGTATGAGGCGAGAAAACTTGGTGCCATCCGGGCGC
core_175	AAGGTTTATATGTAAGCGATAGCTTAGA
core_176	TGTCCACTAAATCGTTGAGTAATCAAAAGAATAGCTTTTCA
core_177	AATTAACTATAATCGTACTATGGTTGCTAGCGGTC
core_178	TTCTTTGCAGAATCGCACGTATAACGTGCGCTAGGGGCGAACGG AATTACCTGAGA
core_179	CACTTGCTTTTAGACGTTAGAATCAGAGTTAGAAAGCGAAAG
core_180	AAAAATTCCTGTAAACGTCACCAATGAAAGCTGATCATCAATAAG CAAA
core_181	TATTCTAAGAACGCCCAATAGCCAAGTACCGCACTCATATTAGAG CGTCTCGCGTC
core_182	ATTGTGTGGTTTAACTCATTCTACCACATTCAACTAACGAACTGGG AAG
core_183	GGGTATCAACCATTCGCCATTCCTTATCCAATCGG
core_184	ATACCATTGCAATTAACATCTG

core_185	CCACCAGACCTGAAAGCGTAAAAAGGGATTGACGC
core_186	AGAAGTAGAATTGAACCCTCAATCAATACCGCCTG
core_187	TCTGCAGAAGATAAATCCTTAC
core_188	CAGTTGAAAGTTAGACTTTTGCCCGAACGTTGAACGAACAACAGT ATATTAC
core_189	CGCCAGCCTACATTCATTCTGGCC
end_01	TTTTTACTTAGCACAGACCAGGCGTTT
end_02	ATTGGGCTGAATTACCTTATGCGATTTTT
end_03	ACGGTGTCGGAACGCGCGACCTGCTCCATGTTT
end_04	TTTCCAGAACGAGTAGTAAGCCCTGACGAGAAACATTT
end_05	TTTCATAGGCTGGCTGACCTTCAAGGCTT
end_06	TTTATTCATCAGTTGAGATTATTACATCATTATACCAGTCATCATTG
end_07	TTTTTAAGAACTGGCGGTAGAAAGTTT
connection_to_pedestal_01	ATAACCCTAACACTGACGTTAGTAAATGAGAATAGCCGATAGTTT TT
connection_to_pedestal_02	CCAGTAACAGTTAATGCGTATAGCCCGGAATCGCCACCCAGAGCC
connection_to_pedestal_03	TTTGCGCCGACACAGCGAAAGACATTGAG
connection_to_pedestal_04	GAGTAACCAAATAAGACGATTGGCCTTGATAGAAG
connection_to_pedestal_05	CATTATACACTAAAACACTCAGAAAGAGGCAAAAGAATACCC
connection_to_pedestal_06	TCCCCGGAACCAGAGCCACCACCGCAGGTCAATCCTCA
connection_to_pedestal_07	AACTTGCATCGGAACGAGGGTAGCCTAAAAC
connection_to_pedestal_08	TCAGGATTCACAAAAGTGCCCGTATAACAGA
connection_to_pedestal_09	CCGCCACGATATAACCCCTGCATCAAAATCACCTACAT
connection_to_pedestal_w_ spring_01	CACCGAACAGTTAATGCGTATAGCCCGGAATCGCCACCCAGAGCC
connection_to_pedestal_w_ spring_02	TCCTCGCATCGGAACGAGGGTAGCCTAAAAC
connection_to_pedestal_w_ spring_03	ATAACCCTAACACTGACGTTAGTAAATGAGAATAGCCGATAGTTA ATTT
connection_to_pedestal_w_ spring_04	CCGCCACGATATAACCCCTGCATCAAAATCACCCACCA
connection_to_pedestal_w_ spring_05	CTCTGGCGCCGACACAGCGAAAGACAATTAA
connection_to_pedestal_w_ spring_06	GCCGCGGAACCAGAGCCACCACCGCAGGTCAATCCTCA
connection_to_pedestal_w_ spring_07	GAGTAACCAAATAAGACGATTGGCCTTGATGCCAC
connection_to_pedestal_w_ spring_08	AGGAGTACACTAAAACACTCAGAAAGAGGCAAAAGAAGTTGA
connection_to_pedestal_w_ spring_09	GAACCATTCACAAAAGTGCCCGTATAGAACC

Table 4 Oligo Sequences used for the rotor arm part 2 (extension)

core_01	TCTTACCGGGAGGTGGTATTCTAAGAACCAAGCAAAACCAAG
core_02	TATTGGGGTTGCAGCAAGCAAAAGGGAC
core_03	AGAGGCGGGTAACCGTTGTAGTCCAGAA
core_04	GCATTAAACCCTTCTTACATTGGCAGATCAGGAAACCTTGCT
core_05	CTGTCGTACTTGCCATAATCACGCCAGAATCCTGATTATGGTTG
core_06	CTCACTGTATTTTTTAAAAATACCGAAC
core_07	TAATGAGAGGATGCCGGGTTATGGTGCT
core_08	GGGTGCCATGGTCAGGGTACCGAGCTCGCTCAGAATGCGGCGGGCCGTT
core_09	AAGCATAGTGAAATTCCTCACAGTTGAGTTCACGG
core_10	ACATACGCATCAGTAAACATTGGGTAAAGGTTTC
core_11	CAGGGTTGGCTTAGTCCCTTAGAATCCT
core_12	CTTCGCTAACCAGGTTCCGGCACCGCTTGGCGCATGGGATAGTGCAGAA
core_13	AAGGGCGCCATTCAAAGATCGCACTCCATGCCAGTAAACGGCAACAATA
core_14	AGCATTAACATCACGGAGACAGGCATTTTTTAACC
core_15	ATCAATTAATTAGCACATTATGACCCTGCGCAAGGGTAGGTA
core_16	TTTAGCTCTGAACAGAAGCGCATTAGACATCCAAAATAAACA
core_17	TCGCAAAAGAGGGTAGAGAATAACATAATTGTTTAGCCTAAT
core_18	AGTTTGAATCAGAGGAAGCCCTTCAGCCTTTACAGAATTGAG
core_19	ACGGTGTAATATAATTGATAAGAGGTCA
core_20	TGCAAATTAAGAATAAATCAACAGTTCAGAAAATAGTAAAAAACGTC
core_21	TCATCAACCCAAATGCTTGAGATGGTTT
core_22	GGCGCATTGCTCATCCAGAACGAGTAGTATGCGAT
core_23	CGGTGTATTGTTGAGGCAGGTTTACCGTTCCAGTTAGTTTGCACCAGAG
core_24	TGACCAATATTCTGTGCCCGTATAAACATTGATGA
core_25	TCAATCATAAGAGGGGTCAGTGCCTTGAGGTAATACATTAAATTTTCGG
core_26	GCCGGAAGAGAAGGGGGTTGATATAAGT
core_27	AAATCCGCGACCTGGGTTTTGCTCAGTAAAACACTTGTGTCGGCGGGAT
core_28	CCAACAGAGATAGATGAATCGTCTTTGATAAAAGAGGATTTT
core_29	TATTAGTTTAATTGCTGGTCAAAGGTTAATAATACATTTG
core_30	CGCTCACAATTCTATGTTCTTCGCGTCCTCTGCCA
core_31	GTGAATTTCAGAGTTTGAGCAAAAGAAAAACAGTACATATG
core_32	AATCATAGGTCTGAAACGACGAAACAAA
core_33	GAGACTAATAGCGATAGCTTAATAACCT
core_34	CAAAGCGCCATTCGATCGGTGAGTAACAACGCCATCGCATTA
core_35	GGCAAAGCTACTAAAATATGAAGAGATCTGGAGCAAACAAGA
core_36	AAGCCTCAGAGCGAGAATTAAATATTTTCAGCTACTCAAGATTCAGATA
core_37	CGCTAATCCATTAGACGCAGTTAAGCGAACCTCCC
core_38	GCTTAATTGCTGCTGGAAGAAAGCGGTACCCTGGAATCCC
core_39	ACAAAGCAGGCTGGTTGAGATATAACGCCCAGACGACGAT
core_40	TTCGGAACCTATCTTTGAATTGGCCTAGAGCCGAGCCGCC
core_41	AGCGATTTTGTATCGCGAAAGGATATATGATAGTT
core_42	GCTGGTTTGCCCATAAATCTTCCAGT

core_43	ACCAGTAAGTTACCGCCAGCCATTGCAATCACCAG
core_44	CAATCGTCTGAAATTACCTACTAGAAGAAGGGCGC
core_45	GAACCCTCGTGGCACAGACAACCCGCTT
core_46	GATCCCCTAGCTGTTTCCTGTAAGTGTA
core_47	GACGCAGAAACACCCAGCGCCATGTTTAAACAATCAGTTAAATCCTCAT
core_48	GAAGGGATAGCTTTTGCCGAAAAAATTTTGGATCGGTGGT
core_49	GAAGAGTGTAATCAATATATGTGAGTGAGATTAAG
core_50	ATTTTAGTTAATTTGTTGGGTTATATAAAAGTTGGAAAAGCCCAACAG
core_51	CGAGAAATTTGAAATACCGGTGTAGATGCTAGACAAAGAACG
core_52	CTCAGGGGCTGCGCACAATAAATCATATTTGCGGGAATCGGC
core_53	TAAATCGGTTGTAACCCTCATATATTTATCCCAGGATAAAGC
core_54	AATAGCAATAGCTAAGATAGCCAAAAGACCGACTTAAATATT
core_55	ACAATGATTAAGCCCAATAATACAGTTGGGCAACA
core_56	TTTAATTCAACAGGGCCCGAAAGGCCGGATGTTTAGACTGGA
core_57	AATTTGACCGGATATTCATTAGAGTAATACAGGTAAGGCAT
core_58	ATAGCGGCTGAGACTCCTCAACGAGGCGTAGGAACGCATTC
core_59	GGCGGATAAGTGCCGGAGGTTGAACCGCGAACAACAATGAATTTTCTGT
core_60	AGGCAAAAGAATACTCGACTTTT
core_61	GGCACCAACCTAAATTCCATTCGGCTACAAATCTCGTATCGG
core_62	GAAGAAATCTAATTATTAATCGTATTAAATCCTGAAACTAAT
core_63	ACACCGCAAAACTGATAGCCCTAAAACAGGCGGTC
core_64	GCGGATCAAACTCGCACAGTCATCAACCAGCTT
core_65	CTGCTCACTCACGGCGGAATTTGTGAGAGGAACGGGTTCCGG
core_66	CCAGCAGTTGGGCGCACCGTGGT
core_67	GTAAATCTGAAAACCCTTTTTAACCTCCTTCCCAGAATTAAT
core_68	GTCGCTATTTAACAAAAACAGAAATA
core_69	TCTGAAAGAATAAATTCTGATAAAGTACCGACATTTTAATTG
core_70	TTAATGGACTTTTTTAAATGCTGATGCAGGGATGTTGCGTTA
core_71	GACGACGACAGTGATGAGAAAAAGAAAACAGAAAA
core_72	TTTTTAGACCAAAAAAAATTAAGCAATAGCTGAAAAGCTGAT
core_73	CGATTTTAAACAGGCCCTGAACAAAGTCTGGTCAA
core_74	AGTAAGCTCTTACCAGATAACCCACAAGGAACGAGAGAAAAT
core_75	TTCAAAGCGTTTTTGCGGATGGAGCAAGAAAGAAGGAAACCG
core_76	AACCAGAGCGTTTTGTAGCACCAATACTGCGGAATCGTCACACAGCAAA
core_77	CAAACTCGCTCCTTTGCTGTAGCTCAACATGTTAAGAGTACC
core_78	AATCATAATAAAAAACCATGCCAGAGGGGGTAACGAGCAACACTATC
core_79	ACTGGCTAGAAACATCAGTGAATAAGGC
core_80	CATACATGGCTTGTTAATGCCCCCGCCCTGACGCATAAGCGT
core_81	GTGTACTGTAACAGAAACATGAAAGTATTAAGGGAAAATAAA
core_82	TAGGTGCCACCCTCAGCGGATTTGCTAAACAACTCCCAGCCCT
core_83	GTACTCAGTCGAGAATTAGGATTAGCGGCTCCATGACTGAGT
core_84	TCATGAGGAAGTACGAAAGGACCCCC
core_85	CAATAATTGCGCGTAGAGCGGGATTTAGA

core_86	CCTTGAGATTTTGACGACCAGCA
core_87	GAGAGCCGTAACATCTTTACAAACAATTTCAATAGTCTAAAA
core_88	GCGGCGCACAAAGACGCCGGGCGAGAAGTATTAGATATCATT
core_89	GCCTGTGGCGGAATCAGGCGCTTTCGCACGGGTCACAAATCG
core_90	TCCAGCGTGATTATCCAGCATCAGCGGGCTGGTAACCCTTAC
core_91	ACCTTTTATATCAAAACGTCAGATGAATATACAAAGGTTAGA
core_92	TCCTCTAGGTGAATTATCACCGTCAACTGGCACAAACGTTAGATTT
core_93	GTTGGGATAATCAGGGCTTTTGCAAAAGTCGTTTACAAAAGG
core_94	TCTGAATCAGACGAAGAGGACAGATGAATTTGCCTAT
core_95	CGCAGTCTGTAGCGTTCATAATCAAAATCCCTCAGCCGCCAG
core_96	CTAAAAGATAATAATTCGAGGTGCAGACGTTAGTA
core_97	TCAAATCCAGTCGGGAAACAAGAATA
core_98	TATCAAAAGGAGCACTAACAGTGTTTTTTGCTGAACC
core_99	CCAGCGGTGTTCAGCTGTTGCCCTGCGGGTCATTG
core_100	TACAAGCTGACCGTGTGATAAATAAACATCGAGCC
core_101	CAGCTTTCAGCTGGCGAAAGGAATCCAATCGCAGGTGCCGGAATTACGC
core_102	CATCAACGTCTGGCCGTTAATATTTTGT
core_103	AAATTTTTATTTTAAATGCAACCAATCACGTAAAA
core_104	ACGGCTGTTAACGTGCCGGACTTGTAGACACGCAATATAATC
core_105	TATAAGTGCAGGAAACGCAATTTAGAGC
core_106	TTCGTGCACCCTCATAGTACCGCCACCCACTACCA
core_107	ACTACGTAAACCGTAGTGTTGAAAAGAATAGCCCGATGGTCCAC
core_108	CATCACGCAAATCGTAAACAGGAGGCCGTAGAATCAACCACC
core_109	CACCGAGTTAGTAACTATCGGAACGCTCATGGAAAGGATTATTGACCTGA AAGCGT
core_110	TATCTTTCCCTCAAAATGAAATAAAACAGAGGTGATCGCCATGAATGGC
core_111	TTAACGGCATCATTGTTGCGGTATGAGCCTCAATC
core_112	ACTGGTGTGCCGGTACTGCGCTCATACCGGGGGTTGTGAGCCTGTTATC
core_113	AACGGTGCTCGTCAACGATGAAGGGTAAGGCGAAACGCACACA
core_114	GCAGCCTCGTGGTGCTGGTCTGCACCGTTATACTT
core_115	TCGTCTCGTCGCTGCAAACGCCGACATA
core_116	GAGGCGATTTACATCGGGAGACAGGTTTAATTATT
core_117	ACCAAGTACGGATTCGCCTGAAAAAGAAATTGCGT
core_118	TAGGGCGCTTTGAATTAATAATTACTAGAGTAACGC
core_119	AATTCGCATTAAATCCGTAATCGTAACCGTGCATCGCCAGCT
core_120	AATAGGAACCCGTCGGATTCCTGTTGGG
core_121	GCTATCAGTCAAATAAAAGGGAGCCTTTATTTCAATAATACTCAGGCAA
core_122	TTTGAAGCCTTAAAAATTTTATTACAAATAAGAAA
core_123	GACTTGCAACGCTAACGAGTTATACATT
core_124	AGTTTATTACCAGCGCCAAAGGGAAGGTGAGCCAT
core_125	CGGAATAACATACAGAATACCCGAACAAAGTTACC
core_126	CGCAAAGGCGACATTTAAATATTCATTACTATTATAAAGAAA
core_127	AGTAAGAGAATGACCGGAACAACATTATTCTTGACAAGTTAAATA

core_128	AATTACGGAAAGATTCTACGTTTGTGAATTACCTTAAATTGGCAACGTA
core_129	CATTGACAGGAGCACACCGGA
core_130	CACCACCTGATATTTGGAAAGTACAGGA
core_131	GCCGCCACACCCTCAGAGCCATTGCCCC
core_132	CACAGAACCCTCAGATCTAGCAAGCCCAACAGACGG
core_133	TACAAATAAAGTTTTGTCGTCTTTCAAGAGTTAAAGCACCAG
core_134	TTGCAGGTTTCTTAAACAGCTGCTTGCTTTTTTTC
core_135	AGACAGGCGTATAATAATGCGCCGCTACACTCAAATAACATCGCCAGCT
core_136	AGATTAGAGCCGCGACAACTTTTAAA
core_137	AGGATTTCGGAAAGGAATTGAGGGTTGGCACCACGCTAGTATTA
core_138	GTACCTATTATTCATTTCAATTACCTTAGCAACCGCGTAACA
core_139	AGAATCGCCATAAGAGAATTCCAGAC
core_140	AATTTAGAACTTCCTGTAATTCTTACCAGTACGCCAACATGT
core_141	AATTTTTAAACAGGGATAAGTCCTGAACGGCTCCGTGGGAACTTGAGGG
core_142	AGCAAATAGTTGCTAAATGCCGGAGAGTGAACGGTAATAGCA
core_143	TAGAAGGCATCGTATCATTCCAAGAACGTTGCCAGTCCTGAATAACCTG
core_144	CCTCAAATAGCGTCCATTACCATTAGCAAGACTTCCAAAAAGCTAAAGT
core_145	TGCTTTAAAAAATCAGGTCTTATTGCATAAATATCCCGGAAG
core_146	ATAACCCAAGTTTTCGATAGCAGCACCGAGAAAAATCATCAGCTGACCT
core_147	AAAAACCACACTAATGCAGATACTTAGGAATCAGGACTTTAAGA
core_148	ACCGCCTCACCGGACTTTAGCGTCAGAC
core_149	ACCCTCACCATCTTCGTTTTCATCGGCAGCCAGAACACAAACACCGAAC
core_150	CATAGTTAGCGTATGGGAT
core_151	AACGATCCTACAACGCCTGTACCATGTAAGAGCCATATCACC
core_152	GCTTGATGTAGCGGTCACGCAGGGTTGCTATCAGGTTTGCG
core_153	CTTTGACGAGCAAACGGTAGTGAGGC
core_154	GAGGTGTCAGATGATGGCAATGCGGCCTTTAGTGAGCACGCGTG
core_155	ATGGAAGGAATGCCAACGGCAGGTCAGC
core_156	GCCATCCACGTCAGCCGGCCAGAGCACACGATGCTAAAAAGCTAAATTT
core_157	AGATTTTAACAATATACAAAATCGCGCACATCAAGTTGAATTTGCTTCT
core_158	AGTAATATTTAACAATAAAGCCAACGCTTGTTTAGGTTAAATCCTAAAT
core_159	TAAATTGTAGCAGAGGCATTTACATGTTCAGCTAAGTCACGT
core_160	AAATATTCGCGCCTGTTTATCGGATTGAGTGAGCGCGGGCCT
core_161	AAGATTGTATAAGCTAAAATTCAAAAAT
core_162	GCCCCAAGTTAAATCAGCTTCATTGCCTTGATAAT
core_163	GTCAATCCGAGCATGTAGAAATGCCTGACCGTTCTAGGTGGC
core_164	CTAGCATGAATCGAGGTAGCTATTTTTGTATTCAAGTAATGTATAAAAA
core_165	CATTAACATCGAGAAGCGAGGCGTTTTGAAAATTCAATTATT
core_166	GACGGAATATGGTTTTTGTCACAATCAAATGTTAGTGATTAATAAGAAA
core_167	ACCGATTGAGGGAGACAAAAGGACACCA
core_168	CCAAAATAGCGAGATAGCGACAGAATCAATACCAGTACCACACAGACCA
core_169	CTTATTAGCGTTTGGAACCGCCCAGAAC
core_170	TTTATCATGATACCTCGGTCGCTGAGGCCGTCACCTTGAGGA

core_171	TGGCAAGCGGGGAAAGCCGGCATCGGAACACCCAA
core_172	ACACCCGGGTAATACAATACTGCCAACGCGCGGGGATTCTGGTCACACG
core_173	CCGCGCTCGTGCTTTCCTCGTATTAAAGGTCTGTC
core_174	GTACTGCCCGAACGAGCATCA
core_175	AGTTTGAAGCAGCATCAATATCGTTGCG
core_176	TTGCGGAAACAGTGAATCAACTGAGCTAACTCACACTTTAATGCGCGTTC GTAATC
core_177	AACCACCAGAAGGACACTCTGCCTGCAGAAGCCTG
core_178	TATCATCATATTCCCAGTGTCGCCCCCTGAGCCGG
core_179	CTGATTGCCCGTAAGATTGCCATAACCTCACCGGACCAGTCCAAAAAGA
core_180	CTGAATATGTACATGGTCCGTGTGGAGCCGCCACGGATAGACTTTCTATA
core_181	ACCAATGACGAACTAACGGAATCAGGATTACAACTTT
core_182	ACCTACCTTAATGGGATGATGGCCAGTGCCAAGCTTTATCAAACGCTGA
core_183	TGCACGTATTTCATAAAACAATCACGACGTTGTAA
core_184	AGGTAAAGTAACACCGGAATCCATTAATTAATTTCATCT
core_185	GACGACAATAAGGCTATCATAGCTGCAAGGCGATTCTATATGCAAATAT
core_186	ATAATATCCCATCCAAGATTCCACCATCTAGTAGT
core_187	TAATTTAATATGTACCCCGGTGAGAGTCTACAAAG
core_188	GCTGTCTGCCATATTTGCACCCATTTGGGGCGCGA
core_189	TTCCTTAGGAATCATTACCGCGCCCAATATAATCG
core_190	GGTATTAGCCGTTTTTATTTTCTTATCC
core_191	TACCGCATACGTCTTTCCAGAACGTCAAAAATGAAAATAGTTGAC
core_192	TTGGGAAAATAACGTAAAGGTATTCCCAATTCTGCAATTGAG
core_193	ATCACCAAATTCGACAGAAGCTTTCATTCCATATAAAGCTTAGA
core_194	GTGAGAATAGAAAGCACCCTCCCGTAACTTACTTA
core_195	TCATACAACAGTTTCATTTTCAGGGACTAGTTTTAACCCGGAA
core_196	ACGTTGAAGAGGCTCTCAGCAATCGCCTGATAAATCATCTTT
core_197	TAAAGGAATTGCGAAACCGCCCGCTTTT
tip_dye_01	Cy5-GGGCGAAGAACCATCCCTAAAGGGAGCCCCCGAGCATGGCCC
tip_dye_02	Cy5-CAGCAGGCTGGCCCTGAGAGACGCCAGG
tip_dye_03	Cy5-ATCAAGTACTCCAAAAGAGTCCGGCAAAATCCCTT
tip_dye_04	Cy5-GGGCGCTAGGGCGCTTGGAACCGTCAAAGTGGTTT
tip_dye_05	Cy5-TTTAGGTGCCGTAAAGCACTAAGAACGTG
tip_dye_06	Cy5-TTTAGAAAGCGAAAGGAGCGCGAGAAAGGAAGGGATTT
tip_dye_07	Cy5-TTTAACAGCTGATTGCCCTCACCAGTGAGACGGGCTTT
tip_dye_08	Cy5-TTTTGGTTCCGAAATCACTATTAATTT
tip_dye_09	Cy5-TTTAGAACGTGGTTTTTGGGGTCGTTT
tip_dye_10	Cy5-TTCTTTTTCACCGCCGAAAATCCTGTTTGATGGTTT
connection_betw._ rotor_arms_01	ACAGGAAAAAAAGTACAACGGAGATATACCAA
connection_betw._ rotor_arms_02	GTCAGTTGCCATCGCCCACGCATAACCACAGCATCGGAAGAGGCGGTCA GT
connection_betw._ rotor_arms_03	AACAGAGATTGCCACTACGAAGCGCGAAACACGCTCATGGAA

connection_betw._ rotor_arms_04	TAAAACAGAGGTCGAGGGTAGCAAAAACGGGTAAAATACGTAAAGAACCCT
connection_betw. _rotor_arms_05	GCGCCGATTTAATTCAAAAAAAAGGCTCCAAACTCGTAT
connection_betw._ rotor_arms_06	AAACAATTCGACAAAGGAGCCCAATGACAACAAGCAAATCAA

Table 5 Oligo Sequences used for the pedestal with torsional spring

core_01	CAAAAGAATCATCGTCCATGTGGTGCTGCGCTGGC
core_02	TCATATATTAGCAATAAATCATAGATAC
core_03	CGTTCTACGGATAAAACCAAAATAGCGCTGCGGGATACCCCG
core_04	AACGCAAAACTAGCATGTCAATATCAGGATTCAAC
core_05	AATTAAGCAATAAAAATTTTTAACCCTAAACGTACAGCGGA
core_06	AGTAGTACAATAACACAGTTGATGCTGTTCATTTT
core_07	AACGGTATCTGGAGTCAAATCACCATCAATCGGCGCGTTTACCCAGAGG
core_08	GAAGCCTTCGGTTGGGTGGCATTTCATTCTGGAAG
core_09	AATTGCTGAATATAATTCCCATTCATTGCTATTACATAAACACGGGGTC
core_10	TAAATATTTAAATTGTGAGCGAAACTCCAACAGGTGTTTTAAGATGGGC
core_11	CTCGTATGCAAGACTGTAATT
core_12	GGCCTTCCTGTAGCTTAACCATCGGCCTTACCCTGAGGCTTG
core_13	CAGCTTTAGAGAGTACCTTTACCGAAAGCATCTGCTGGTGCT
core_14	CACTGCCAATAGGATAGCATTCCTGTGTGAAATTGTACCGAG
core_15	ATATCGCCAGGATTCATCAACATTAAATTTTGTTATCCAGCC
core_16	AAGTTTGGTATTAGCCAAGTTGCAGAGGCGA
core_17	CCTCAGAGTGAATTACAATGACAACAACAGTTTCCTTAATGC
core_18	ATCGCACAATCAGCAGCTCAACATGTTTTTTCATT
core_19	AACCGCCGTTTATCCACGCATAACCGATCTAAAGA
core_20	ACTATTAATAAATAATTCTGCATTTCGCAAATGGTGCATTAA
core_21	AAAAATCCTCAAATCCATATACTGTTTAGCTATATTCAATTCTAACGCC
core_22	ATAAATCCCGCTTTGCATCGGACTCCTCCGCCTGG
core_23	AAACGAGAATGACCAGCTTTCTGGTGTATTCGAGC
core_24	ACCCTCAGATATAAGTATAGCCTATTTCCTTTTTCGTAATGG
core_25	GCTTTAACGATCGGGTGTTCAGCTGGAGTCCACGC
core_26	TTTGAAAAAGGAATCAAACCCTCAATCA
core_27	CTGCAAGGGGTAATCAAAGTAGAGGCGCGGACTTG
core_28	GCGATTACTCTTCGAATCCCCAGGTCTTCAGGAAGAACCGTGACTTCAA
core_29	AGTTGGGTACTAATAGCATAAAGCTAAATTATTTCCGATAAA
core_30	TAAAATAACCTTGCTGAACCTACGCTCAACAGTGC
core_31	GTGAGAGGCTTTTGAGGGTTTGGAAGGGACAGTTC
core_32	TCAGTACGGCTGAGAACGAGGCACTGGTTGCGGGC
core_33	GGAAACAATATGATTCATTGCCTGAGAGATCGTAAGGATAAAGCCTCAG
core_34	CCTCACCGAGAGATATTCATCTAAGGCTAATTGGGCTTGAGA
core_35	ACGGGAAGCTGATAAGATCTACAAAGGCTCATATG
core_36	TCATCGGAAACAGGCCTCGTTGTCAGAGGGTAATTAAAAGGT
core_37	AATACCACAAAGCTATTTCAACTTTAATTGGACATA
core_38	TAAATAAATTTGGCAAATCAACAGTTGATACCGACGCGAGAAAAACAAT
core_39	CAATTTGCCCGAACTCGACAATTTTTTACCTGCAATTTTT
core_40	AAACAGGTAGAAAGAGACTTTGACCTGCCCTGATAGCTTACG
core_41	GTTATTAATTTTAATTATCAGGAGGTGATCGCCATGCCCAATGCGCCGC
core_42	TCATAGGAGAACGGGTCCTGATCCAAATACGGTACCAAGTTT

core_43	TACCGACGAGCGCTGTATAACGTGCTTTAGGCCGACGTCAAA
core_44	AAAAAATCCCGTATGGTTTAGCTCATTGGCCAGATAGAACG
core_45	AAAAAAGCCAGCAGTTGGGCGGTTAATAATTTAGGGTCCCGG
core_46	GGCGGCCAGTAGTATGCCCTGGTCTCGTGTCTGGTGAAGGTATGGTTTG
core_47	AACGTAACATTCAAGAAGAAAAATCTAC
core_48	CAGTGAAAGTTGAGAAACGAACTAACGGTTTGCCGCCGCACA
core_49	TATACTGAATTACCGAAACAGTACATAATTAAGAC
core_50	TTATACTCCTTAGAATCCTTGACCTTGCTAGATTTAAACAATTGCTTTGAA TGATG
core_51	ACCGAACGAACCATAACCCACTATGGTTATAGAAA
core_52	GCACATCAGTCCACATATAAAGAATACCCAAAAGA
core_53	CTTCATCCCGAACTGACCAACATCTTTGCGGGCGCGCGGCTG
core_54	ATCTTGATCACCGTGATAGCAGCCTTTAATTATTC
core_55	TAACCAGCAGAAGAGCCAACAAAAGAACCGTGTGA
core_56	CCGGAACCAACGGAGCATCAGTCCCTTAGTAGCAACAGGGAG
core_57	AACACTCTTTGAAAATAGGCTGGCTGACATTTGGG
core_58	GGTTGCGGGCCGGAAATTAGAGCCAGCACAGGCGCGAGGACA
core_59	AATCCGCACCCCCAGCGATTAAAGGGAAAAGAGTA
core_60	GCGCTTTTTTCTTTTTGAGGAATATTCGTGTTAATTGTATCG
core_61	TTAATTGAGTATTACATCATA
core_62	GTAAAGGCGCACTCAACCATCCACCGACTTGAGCC
core_63	GCAAATCAAAGACATGCGGGAGCCCCCTGTTTTCA
core_64	ATGAGGACATCGCCAGCTTGCTTTCGAGACCGCCAAGGTGTA
core_65	ATTCTTATAACAACTAAAACAATGATGG
core_66	CGGCTACTGAAAGTACAGAATGCCAGAACGCGTAC
core_67	TGTCACTCTGCATTATAAGTGTTATCCTATCCCAAACAAGAA
core_68	AGCGCAGGCGGCCAATAATTTGCTAAACCTCGAAT
core_69	GCGGGCCGCATCAGCGCGCGGGTTTTGCCTAACGA
core_70	AACGCGCCTGTTTATGTTTAATTAAAGGACTGTAGCCAATGA
core_71	CAACTAATCAGCGGTGAGGATCCCCGGGTTATCCGAACTACA
core_72	TCCTTATCCAACAGGCAGATTCATCGTAGGAATCATAAGAAC
core_73	AACTTTCAACAGTTAGGAATTTACCGTAAATTGCG
core_74	TCGTAATCATGGTTCTGTATGGGATTTTTTTCACGCAAGCCCCGCTTTC
core_75	AAAACATCCTGATTCTGTCTTATCCCATCAGCCAT
core_76	AATCAGTTTAAAGGATTCATAAGGGTTGTTAGAGCCGGAACCCTAAAGG
core_77	CCACAGACAGCCCTGGGATAGTTGAAAACACTCTG
core_78	ACGCCTGACCCATGGCGAATAGAATGCGGCATCGT
core_79	AAGTGTTCCCTGAGCAAGCGGGTGTCCAGATTTGTAGTTTTGCAGACGA
core_80	AAGGGACCCTGAGTTACAATTCCGTCGATGAAACA
core_81	ACACTGAGTTTCGTAAAGGAACGGTCATGTCACGT
core_82	ATGTATTATTTGCCGGCCACCTGATTGCCCCCAGCGCAACCAAATTGTG
core_83	CACACGACCAGTACCAAGCAAGCCGTTTCGAACCT
core_84	TTTATCGCACAAATTTTTTCCTGATAACGGAT

core_85	AATATTGCAAAGACATCAAAACGGCGAAAGGTGCC
core_86	TCCAGAGTCAGGATTAGCGGGGGAGAGGTCACATT
core_87	TCACCGTTAAACAGATTAAACGTCACTGTTGCCCT
core_88	GAGGGTTGACTGTCGTGCCAGGCGCGCCGTCGCTG
core_89	TAGAAGGTTACCGCAGCTAACCGGTTTGTGCCGGTTCGTCAC
core_90	GGTATTCTTACCGCGATTATTTACATTGAGATAGAGATTAGTACCAACG
core_91	TATCCAGCTTCTTTACCCTTCTAATCGGGTTTGGATTCCTGA
core_92	CTGGTAATTGCGCTCGGCCAAACGATCCTTAAAGG
core_93	AACTATCTCACTTGATTCTGGCATTCCATCCATCAATATAATAGCGATA
core_94	CCTTCACAAGAGAAGGATTTTAGCAGCGGTTAACGACTGTTGTCCCAGT
core_95	AATAACAGGCCTTGGCGAGGCGTTTTAGTTATTTTCACCAGT
core_96	AACGTCACGCGTTTCCCCTGCCCGGAATCCCTCAG
core_97	TGAGGAAGGTTATCCCTAAATTATTTTATTTAGAA
core_98	GCACCGTATTGCAGCGCGAAAAGTAAAAACTATCAAGAACCCATCGATG
core_99	ATTATTCATTTAGCAAAAAAACAAATTTTACA
core_100	TGAATTAACGTGCCAGACGGTAGCCATGTTTACCA
core_101	TTATTCAAGTGCAGAGAGTCGATTAAGACAGGCGGAATGAAT
core_102	CCAGCGCACGGAAATCAGCGTTACTTAGAATTTGT
core_103	GTAAAGCACTGGCAATAAAGGTGGCAACTATTAAA
core_104	ACTAAATTTGACGGGCTATCTGAGTTAATAAAAATAAATAAT
core_105	CCGAGATTGGTTTATACAGGGTCCTGAGATTATTTGAATCTT
core_106	AGTTACAGCGTCTTAGCAATAAACAATACTTATCC
core_107	GACTCCATACATACTGATTAAGACTCCTGGGGTCG
core_108	ACGTCAACTTATAAAAAAGGGCCGCCGCTCAGTGA
core_109	AAATAAACCTAATTTAAAACAGGCGGTCAGAATCGTCAAATATTAATGG
core_110	TCGGGAGTCAGGTTTAACGTCTATGTGAGTGAATA
core_111	GAAGATAACTTTACAACTTTTCCATATTCCAGTAT
core_112	TTTATAAGCTTAATAATAAGAGCAAGCTATAGCCCTACGAGC
core_113	AATGAAAAATGCAGAAAGTAATTCTGTC
core_114	CGTGGCGCGAAACAATGAAATAAAGCCACAAATAT
core_115	AGCAATAGGAAAGCGAATAGCGAACGTGAGCCTCC
core_116	GAGCGGGAGAAAGGAAAATCCAGGGCGATTTTTTCACGAGAA
top_01	TTTTTAGTAAATAAAAAAGGCTTTTTTTCCAAAAGGAGCCTTG
top_02	TTTAACATAAAAACAGGGCCCTTTACAGAGAGAATTTT
top_03	GAACAAAAGAATCAGAGAAGCGCATTAGACGGGTTT
top_04	TTTGATAGCCGAACAAAGCTTAAGAAGAGAGAT
top_05	AAGAAACACCCAGCAGATCAAGATTAGTTGTTT
top_06	ACACCCTCAGACGACGACATTTTTTATAAACAACATG
top_07	TTTGGAATAAGTTTGGAACAAGCTCATAA
top_08	TTTCTATTTTGCGATTTTTTCAACAATATAAAG
top_09	TTTTTGTCACAATCAGCTTTGAGACAGGA
top_10	TTTCATAACGCCAAACAATCATTACCAAG
top_11	TTTACCGAGGAAACGCAATGAGTTACCAGAAGGAATTT

top_12	GAACTCACAGTCGGGAAACGCCACCATTT
top_13	TTTAGTAAGAGCAACTGTTTAGACTGGTTT
top_14	GGAACCTGCGTCAGGATTTTACGAGCACAATATCAAAGTAAGCATTT
top_15	TTTACAGTGCCCGTAACTCAGGAGGTTTTT
top_16	AAGAATTTACCGAATATACAAATATCTGGTCAGTACTAGAAAAAGCCTTT
top_17	TTTTGCGGAATCGTCTAGTCAGAAGCATTT
top_18	TTTGAGAATCATCTTTTTTGTACCGCAATAA
top_19	TTTAAGCGGAACGACGACAGTAATAGGAA
top_20	TTTAAAAAGATTAAGAGGAAGCATTGCTC
top_21	TTTCCCTCATTTTCACATAGTTAGCGTTTT
top_22	AGAGGCTGCTCGTCGCCAGCTGGCGACCAATACTTT
top_23	TTTGCCACTACGAAGGCGGGGGTAAATGCGCCGTCTTAAACAGCTTGAT<br>TTT
top_24	GATGAACAAAGAATCCACCAACCTAAAACGTTTTTTAAAGAGGCA
top_25	GAACCGGATTTTTTTATTCATTACAATTACCTTTTT
top_26	TTTATGCGATTTTAAGAACCATTGTGCCAAATCCGGACAA
top_27	TTTTCAGGACGTTGGCTAATGCAGATATTT
top_28	TTTGGATGGCTTTAGATTTAGTTTTTTTTTGACCATTACAGGCAATTT
top_29	TTTTAGGTTGGGTTATATAAAATCCTTTGAGAGACT
top_30	TATGCGTGCCCTTTCCCCGATAGTGTTGTTCCAGTTTATTTTT
top_31	TTTATTTTAATTCGCGTCTCTTTTGATAAGAGGTCTTT
top_32	TTTAGTGAATTTTAACCTCCGGCTTTT
top_33	TTTAAAAGGGTGAGAAAGGCCGATTACGAGGCATTTT
top_34	AAGAGAATAGATAAGTATTAAACCAATTT
top_35	GCTTAGAATCAATAAGATGAACAGTACCCATCAAGAAAACAAAATTAAT<br>T<br>ACATTTT
top_36	TTTTGTTTAGTATCATAGGCAGAGGCATTT
top_37	TTTTTTTCGAGCCAGACCTTTTATCAAAAGCTGAGAAGAGTCAATTTT
top_38	TCTCCAAGAATTTTGTCGTCTTTCCAGACGTTT
top_39	GAGACAGCAAACAAGATGTGTAGGTAAAGATTCTTT
top_40	TTTTGATGCAAAAATAAGAATTTTTTTAAACACCGGAATCATA
top_41	GAACGAGAGAGCTTCGCCATCAAAAATTGCTTT
top_42	TTTGGCAAAGAATTTTAAATGTTTTTTCAATGCCTGAGTAAGA
top_43	TTTTTTTGAAGCCTTAAAACCCGACTTGCGGGAGGTTT
top_44	TTTGCACCATTACCAGGTCATAGCCCCCTTATTAGCG
bottom_01	CTGCTCAAACAACATTATTGAGACGCAGAAACATTT
bottom_02	TTTGTAATGGGATAGACCGGGGGTTTT
bottom_03	TTCAAAGCGGAAGCAGTAACAACCCGTCGGATTCTCCGTGTTT
bottom_04	TTTGGAACAAACGGCGGCAGACCGAACATTGACCTTT
bottom_05	TTTTTTGTTAAAGCTTCTGGTGCCTTT
bottom_06	TTTATTGTATAAGCAAATATAGCACGACGTTGTATTT
bottom_07	TTTGGTGGAGCCGCCGTGAAGGGATAGTTT
bottom_08	TTTGTAAACGGTGCCATTCGCCATTTT

bottom_09	CGGCACCATTCGCAGCAACTAAATTAATATTTT
bottom_10	TTTGCGGATCAAACTATGAAGGGTAAAGTTAAACTTT
bottom_11	TTTGATGCTGATTGCCGTTCACCAGAACGTTTAGTGTAAATTT
bottom_12	AGAAAGCACGGTGTTGGGGCGCGTTAAATTTTT
bottom_13	CTGAAAATACCAAAAACATTATGACCCAGGAAGTTT
bottom_14	TTTTCAGGCTGCGCAGCATCAGATTTT
bottom_15	GTTGATATTTTGAGAATTAATGCCGGAGAGTTT
bottom_16	TTTAAACGACGGCCATCGGTGGTGTTT
bottom_17	TTTTTCTGCCAGAAAGCCTGGGGTTTT
bottom_18	TTTTTCGCGTCCGTGAGCCTCCCAACATACGAGCTTT
bottom_19	TTTCGTAAAACAGAAATAAAGATTATCATGAATTATACACCGC
bottom_20	TTTCTCTCACGGAAAACCGGCAAATTT
bottom_21	TTTGCCTAATGAGTGCAGCCATAAATTAAAAGAATACGTGGTTT
bottom_22	TTTGTCTTTAATGCGCTGCAACAGTGCTTT
bottom_23	TTTACGGGCAACAGCGAGTAAAAGTTT
bottom_24	TTTGGTAGCTATATCAGAAAAGCCTTT
bottom_25	TTTTGTTTGAGATTGAGGGAGGCAGCAGCAATCCGCCTCCGTG
bottom_26	TTTTCCGAAATCGGCAAGGGAAGATTT
bottom_27	TTTCAGGGCGTGTTAGCAAACGTAGAAAA
bottom_28	TATTACGCAGTAATGGCCCACTACGTTTT
bottom_29	TTTGAACCATCACCCAAATCAAGTTTTTT
bottom_30	TTTAAGCGAAAGAGCAAATGAAAATTT
bottom_31	TTTACGGCAGCACCGTCGAAACCGCAAGAATGCCATTT
bottom_32	TTTAGTCTGTTAGAATGGCTATTATTT
bottom_33	TGCAACAGGAAATTTTTTAACGCTCATGGAAAAGATA
bottom_34	TGAAATGGCCCAATAGCAAGCAAATCTACCTACTTT
bottom_35	TTTATTTTGACGCTCAATCGTCAAAGCGTCCGTTGT
bottom_36	TTTGCCGGGTTCTTCACCAGTGAGTTT
bottom_37	CGAACTGAGGGCGCACCACACCGACATTCAACCTGGTGGTTTT
bottom_38	TTTTAATGTTTTTTTTTTTTTAACAATTTCATTAGTAA
bottom_39	TTTCACGCTGAGAGCTCTCAATAGTTT
bottom_40	TTTAGCCAGCGGCGTATTGGGCGCTTT
bottom_41	TTTCTAATAGATTAGAGCCGATCTTCTGA
bottom_42	TTTATAATACATTTGAGGAGTTAATTCAAGGGC
bottom_43	AGTAACAAATTGCGTTCTGTAAATCGTCGCTATATTTGCATTT
bottom_44	TTTGCGGAACAAAGAATTTTTTACCACCAGAGGGTTAGAACCTTTT
bottom_45	TTTACCATATCAAAATTTAATTAATTTTCTCTGAATAATGGAAAGGAGCG
bottom_46	TTCCACATCACAGTAGTGAGAATAGCACGCGTGCCTGTTCTTT
connection_to<br>_triangle_01	GTTGTGTACATCGCTCATTATACCAGTTTTGACGATCCATCGCCCAGGGC<br>GCTG
connection_to<br>_triangle_02	AATAACGAGAAACGCAAAGACACCACTTTTGATCGGTCCGCGGTGCGG<br>G<br>GAACGCCACTGGC
connection_to	AAGTTTGAGCCCGCCCACTCGTGCAATTTTACAGACAAATCACCAGTATT

_triangle_03	
connection_to_triangle_04	TTTAGAATTAACTGATTTTCGTAGACAGCACCGCGTGCGT
connection_to_triangle_05	GGCGTTATCCAATCTACTATATGTAAATGCTTTCTGAGTCGCTCTCTCGCA ACAGAGGTACCTC
connection_to_triangle_06	GTGACGATAGAGGTCATCATATCCTTTTAACGATCTAAAGTTTCATAGCTGTTT
connection_to_triangle_07	TAACACGAAAGCACCGGTTAAGCAAAATTTTTCAGCTATAGCAGGGGAGCTCATTTTC
connection_to_triangle_08	TTTTAGTACCGCCACTTTTAACATTAGCACTCACGACC
connection_to_triangle_09	CAGTTTGAGGGGTTGCATCTTTTGTTTACAAGCGGTGGC
connection_to_triangle_10	TCACCCTTAGATACGTGCGAAAAATTTTATAGCGTAAGGGGGATGTGCATCCAA
connection_to_triangle_11	TATGCGGCGATTGAGTCAGGTTTTTCGATAGTATACGTAATTTT
connection_to_triangle_12	TTAGCAAGTATGAGACACTAATTTTTTCACCGCGCCACATAGCA
for_biotin_anchor_01	ATGCGGCCTTCGTCGTTTCAGGGTGGTTTTCCCCATCACTTT
for_biotin_anchor_02	ATGCGGCCTTCGTCGTTTCCATCCCACAGGCGAAAATCCTTT
for_biotin_anchor_03	ATGCGGCCTTCGTCGTTTCCAAAAACTGTAATACTTTCAAGCTTTCAGATTT
for_biotin_anchor_04	ATGCGGCCTTCGTCGTTTCGCGGTCCGAAAACCGTCTATTTT
for_biotin_anchor_05	ATGCGGCCTTCGTCGTTTCACGCTGCGCGTAACCTGGCAAGTGTAGCGGT TTT
for_biotin_anchor_06	ATGCGGCCTTCGTCGTTTCGGAAGCATAAAGTGTCACCAGTACACTCACAA
for_biotin_anchor_07	ATGCGGCCTTCGTCGTTTGGAAACCAGGCACCTACCTGCTTT
for_biotin_anchor_08	ATGCGGCCTTCGTCGTTTATCTAAAGCATCTCTTTAGGAGCACTAACAATTT
for_biotin_anchor_09	ATGCGGCCTTCGTCGTTTCACAGACAATATTTGAAACCAATCAATGACCG
rotor_arm_dock_01	AGCCAGAATCTAAACAGGTCAGACGATTGGCCA
rotor_arm_dock_02	GCGCAGTACCGTTCCAGGCCACATTGACGGCAAATAAA
rotor_arm_dock_03	AGAGCCGCCGCCAGTCTTTTCCGGAACCGCGGAAA
rotor_arm_dock_04	CCCTCAGAGCCGCCCCACCACATAATCAAAAT
rotor_arm_dock_05	GAACCACCACCCCACCCTCAGA
rotor_arm_dock_06	AGAGACCCTCACCTCAGAGCCTTGATATTCAC
